# Bioinspired total synthesis of katsumadain A by organocatalytic enantioselective 1,4-conjugate addition

**DOI:** 10.3762/bjoc.9.182

**Published:** 2013-08-06

**Authors:** Yongguang Wang, Ruiyang Bao, Shengdian Huang, Yefeng Tang

**Affiliations:** 1 The Comprehensive AIDS Research Center, Department of Pharmacology & Pharmaceutical Sciences, School of Medicine, Tsinghua University, Beijing, 100084, China

**Keywords:** bioinspired synthesis, catalysis, katsumadain A, natural product, organocatalytic 1,4-conjugate addition

## Abstract

Katsumadain A, a naturally occurring inﬂuenza virus neuraminidase (NA) inhibitor, was synthesized by using a bioinspired, organocatalytic enantioselective 1,4-conjugate addition of styryl-2-pyranone with cinnamaldehyde, followed by a tandem Horner–Wadsworth–Emmons/oxa Michael addition.

## Introduction

2-Pyranone is a privilege structure that is often present in natural products and pharmaceuticals, many of which exhibit diverse molecular architectures and biological profiles [1–2]. For example, katsumadain A (**1**) and B (**2**), which were isolated from *Alpinia katsumadai Hayata* (Zingiberaceae), a chinese herbal drug used as an anti-emetic and stomachic agent, are two natural products bearing a diarylheptanoid scaffold that is incorporated into the styryl-2-pyranone moiety [3]. Preliminary biological evaluations showed that **1** and **2** feature anti-emetic activities on copper sulfate-induced emesis in young chicks. More recently, Rollinger et al. disclosed that katsumadain A (**1**) exhibited prominent in vitro inhibitory activity against the human inﬂuenza virus A/PR/8/34 of the subtype H1N1 (IC_50_ 1.05–0.42 μM) by targeting the enzyme neuraminidase (NA) [4–5]. Moreover, it also inhibited the NA of four H1N1 swine inﬂuenza viruses with IC_50_ values between 0.59 and 1.64 μM. Therefore, katsumadain A represents an attractive lead structure for the anti-flu drug discovery [6].

We recently reported the biomimetic total synthesis of katsumadain C [7], a natural product isolated from the same resource as katsumadain A and B [8]. As part of our continuous interest in the synthesis of bioactive 2-pyranone-derived natural products, we launched a project aiming to develop a highly efficient route for the synthesis of katasumadain A as well as its analogues, which would pave the way for their application in further biomedical investigations.

**Scheme 1:**
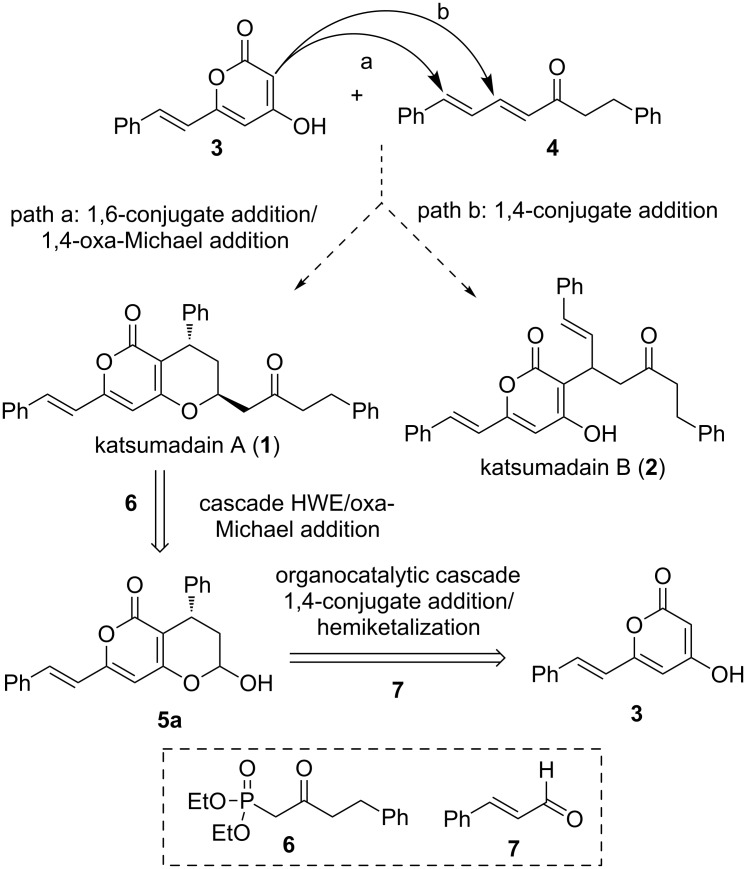
Proposed biosynthetic pathway and strategic analysis for synthesis of katsumadain A.

Biosynthetically, katsumadain A is assumed to be derived from styryl-2-pyranone **3** and alnustone (**4**) [9] through a 1,6-conjugate addition/oxa-Michael addition cascade reaction (path a, Scheme 1). Indeed, both **3** and **4** are known natural substances. Apparently, the biosynthetic pathway represents the most straightforward and convergent approach to synthesize katsumadain A. However, its efficiency might be limited to some extent, given that α,β,γ,δ-unsaturated ketone **4** could undergo a competitive 1,4-conjugate addition to provide the other natural product katsumadain B (path b, Scheme 1). Actually, the regioselectivity of a conjugate addition with α,β,γ,δ-unsaturated Michael acceptors remains a considerable challenge, as it is heavily dependent on the steric and electronic nature of the substrates [10–11]. Moreover, the enantioselective 1,6-conjugate addition to acyclic dienones or dienoates monosubstituted at the β- and δ-position has rarely been investigated [12–15], thus leaving open the question of whether or not a biomimetic approach towards katsumadain A might succeed. Keeping these concerns in mind, an alternative strategy was designed as a fallback, in which katsumadain A could be accessed from the lactol **5a** and phosphonate **6** via a tandem Horner–Wadsworth–Emmons (HWE)/oxa-Michael addition reaction [16]. In turn, **5a** could be derived from **3** and cinnamaldehyde (**7**) by an organocatalytic enantioselective 1,4-conjugate addition followed by the hemiketal formation.

## Results and Discussion

**Scheme 2:**
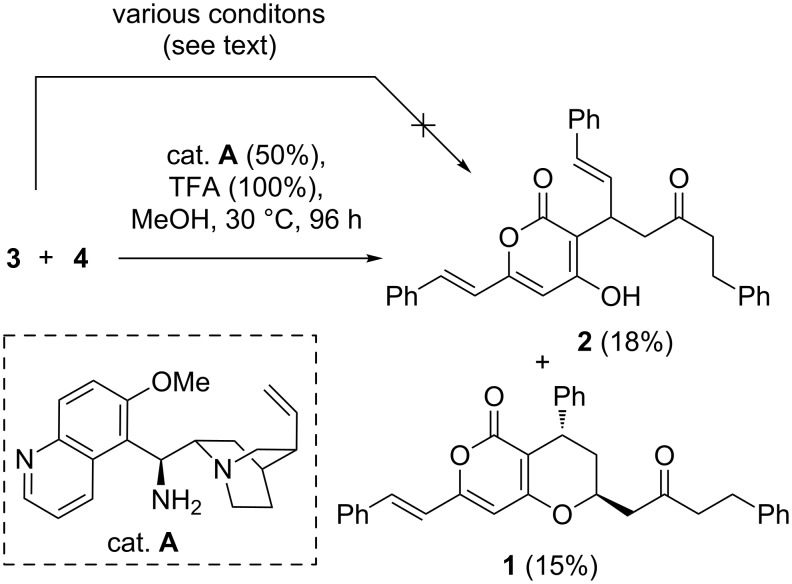
Preliminary results of the biomimetic synthesis of katsumadain A.

Our investigation was initiated by an investigation of the conditions that could effect the proposed biomimetic approach towards katsumadain A and katsumadain B. Both styryl-2-pyranone **3** [17] and alnustone (**4**) [18] were synthesized according to the literature methods. First of all, the 1,6-conjugate addition of **3** towards **4** was attempted by employing various conditions, including different basic conditions (NaH, DBU, KHMDS) by activation of the nucleophile **3** or acidic conditions (AcOH, TMSOTf, Sc(OTf)_3_ and In(OTf)_3_) by activation of the electrophile **4**. However, all of these reactions failed to provide satisfactory results and only lead to the recovery or the substantial decomposition of the starting material. We then turned our attention to the organocatalytic conjugated addition reaction. Among the various documented conditions [19–23], the 9-amino-9-deoxyepicinchona alkaloid-promoted Michael addition is particularly attractive, mainly due to the availability of the catalyst and its superior reactivity towards the activation of the unsaturated ketone substrates through formation of the corresponding iminium intermediate [22]. To our delight, when we tried the standard conditions (30% catalyst **A**, 60% TFA, DCM, 96 h) in our case, we isolated a product in 25% isolated yield, which was proved to be the 1,4-adduct katsumadain B (**2**). Encouraged by this result, we further optimized the reaction by screening different solvents (CH_3_CN, THF, DMSO and MeOH) and additives (HCl, TFA and DMAP), aiming to improve the efficiency and selectivity (1,4- or 1,6-adduct) of the reaction. In most of the cases the 1,4-conjugate addition proceeded dominantly, while no or only trace amounts of the 1,6-adduct katsumadain A (**1**) was observed. The best result was obtained when the reaction was performed with a substoichiometric amount of catalyst **A** with MeOH as a solvent, in which katsumadain A and B were isolated in a 5:6 ratio with a combined yield of 33% (Scheme 2).

With limited success regarding the the biomimetic synthesis of katsumadain A, we then moved towards the alternative approach as described in Scheme 1. We envisioned that in this scenario an organocatalytic 1,4-conjugate addition [24–27] between **3** and **7** would circumvent both the reactivity and selectivity issues, which we have struggled with in the aforementioned studies. To validate this hypothesis, we performed a systematic investigation of the organocatalytic 1,4-conjugate addition by examining various reaction parameters, including organocatalyst, acid additive, solvent temperature, and reaction temperature (Table 1). The first reaction was performed by stirring a mixture of **3** and **7** in DCM at room temperature for 12 h in the presence of Hayashi catalyst **B** [28]. It was found that the desired product **5a** was obtained, albeit in moderate yield and enantioselectivity (Table 1, entry 1). To our delight, the usage of benzoic acid (BA) as an additive could dramatically improve the reaction by affording **5a** in a good yield (78%) and a good ee value (91%, Table 1, entry 2). Besides the catalyst **B**, both Jørgensen catalyst **C** [29] and MacMillan catalyst **D** [30] were also tested in this reaction, but gave inferior results (Table 1, entries 3 and 4). As to the acid additive, *p*-nitrobenzoic acid (PNBA) was found to afford **5a** in an excellent yield, but with a decreased ee value (80%). Furthermore, the solvent effect was also examined. Among the several solvents examined, both MeOH and DMSO proved to be suitable solvent systems (Table 1, entries 6 and 8) by furnishing comparable results with DCM, while MeCN and toluene led to modest results (Table 1, entry 7 and 9). Finally, we found that the reaction temperature has some influence on the outcomes, with a slightly improved enantioselectivity (92% ee) obtained at 0 °C (Table 1, entry 10 and 11). Although the best ee value (93%) was achieved at −20 °C, the reaction became sluggish and the yield dropped to 45% (Table 1, entry 12). It is noteworthy that **5a** was isolated as a mixture of C-5 diastereoisomers (β-isomer/α-isomer = 5:1 to 7:1) in all of the above cases. For convenience, the ee value of **5a** was determined with the corresponding lactone derivative. Furthermore, the absolute stereochemistry of **5a** (β-isomer) was assigned as (7*S*,5*R*) by using the Mosher ester method (see Supporting Information File 1 for details).

**Table 1:** Condition screening of organocatalytic 1,4-conjugate addition/hemiketalization of styryl-2-pyranone with α,β-unsaturated aldehydes.

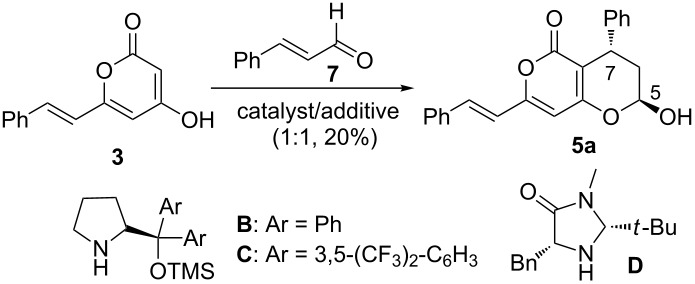						

Entry^a^	Catalyst	Additive	Solvent	*T* (°C)	Yield (%)	ee (%)^b^

1	**B**	none	DCM	23	41	78
2	**B**	BA	DCM	23	78	91
3	**C**	BA	DCM	23	41	−81
4	**D**	BA	DCM	23	10	nd
5	**B**	PNBA	DCM	23	94	80
6	**B**	BA	MeOH	23	80	91
7	**B**	BA	CH_3_CN	23	78	75
8	**B**	BA	DMSO	23	79	93
9	**B**	BA	Toluene	23	66	91
10	**B**	BA	MeOH	0	78	92
11	**B**	BA	DCM	0	82	92
12	**B**	BA	DCM	−20	45	93

^a^Each reaction was run with **3** (0.5 mmol) and **7** (0.6 mmol) in 2.0 mL solvent as shown above. ^b^The ee value was measured with the corresponding lactone product of **5a** using chiral HPLC.

To evaluate the substrate scope of the reaction, we then examined different substituted styryl-2-pyranone and cinnamaldehyde derivatives as Michael addtion donors and acceptors (Table 2). When styryl-2-pyranone **3a** remained unchanged, a variety of cinnamaldehyde derivatives (**7a**–**f**) bearing either electron-withdrawing groups (4-Cl, 4-CF_3_ and 4-NO_2_, Table 2, entries 2-4) or electron-donating groups (4-MeO or 3,5-MeO, Table 2, entries 5 and 6) on the phenyl ring proved to be suitable substrates, affording the corresponding products (**5b**–**f**) in good yields and enantioselectivities. Besides **3a**, the Michael addition donors could also be extended to other substituted styryl-2-pyranone derivatives (e.g., **3b** and **3c**, Table 2, entries 7–11), all of which gave acceptable results. As proof-of-concept cases, the above outcomes indicate that the developed organocatalytic enantioselective 1,4-conjugate addition could be potentially applied to the synthesis of various bicyclic compounds bearing different aromatic moieties (Ar^1^ and Ar^2^), which paves the way to access katsumadain A and its analogues for further biomedical studies.

**Table 2:** Substrate scope of organocatalytic 1,4-conjugate addition/hemiketalization of styryl-2-pyranones with α,β-unsaturated aldehydes.

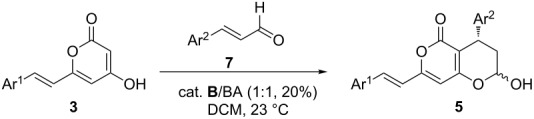			

Entry^a^	Substrate (**3** and **7**)	Yield of **5** (%)^b^	ee value of **5** (%)^c^

1	**3a**: Ar^1^ = Ph; **7a**: Ar^2^ = Ph	82 (**5a**)	92
2	**3a**: Ar^1^ = Ph; **7b**: Ar^2^ = 4-Cl-Ph	59 (**5b**)	83
3	**3a**: Ar^1^ = Ph; **7c**: Ar^2^ = 4-CF_3_-Ph	77 (**5c**)	82
4	**3a**: Ar^1^ = Ph; **7d:** Ar^2^ = 4-NO_2_-Ph	79 (**5d**)	88
5	**3a**: Ar^1^ = Ph; **7e:** Ar^2^ = 4-MeO-Ph	78 (**5e**)	87
6	**3a**: Ar^1^ = Ph; **7f:** Ar^2^ = 3,5-MeO-Ph	70 (**5f**)	92
7	**3b**: Ar^1^ = 4-MeO-Ph; **7a**: Ar^2^ = Ph	84 (**5g**)	82
8	**3c:** Ar^1^ = Furan; **7b**: Ar^2^ = 4-Cl-Ph	88 (**5h**)	84
9	**3c:** Ar^1^ = Furan; **7d**: Ar^2^ = 4-NO_2_-Ph	76 (**5i**)	91
10	**3c:** Ar^1^ = Furan; **7e**: Ar^2^ = 4-MeO-Ph	75 (**5j**)	80
11	**3c:** Ar^1^ = Furan; **7g**: Ar^2^ = Naphthyl	79 (**5k**)	90

^a^Each reaction was run with **3** (0.5 mmol) and **7** (0.6 mmol) in 2.0 mL solvent as shown above. ^b^Each of **5a–k** was obtained as a mixture of C-5 diastereoisomers (the ratio of α-isomer:β-isomer varied from 1:5 to 1:7. ^c^The ee value of **5a–k** was measured with the corresponding lactone product using chiral HPLC.

**Scheme 3:**
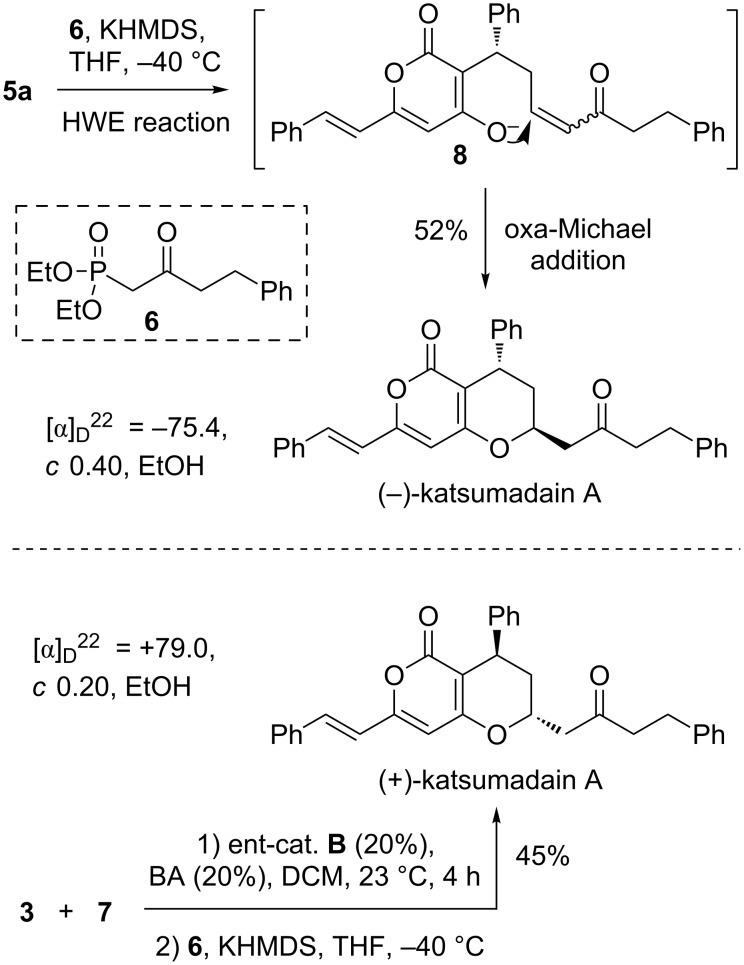
Total synthesis of both enantiomers of katsumadain A.

Having achieved the bicyclic core of katsumadain A in an efficient and enantioselective manner, we then moved towards its total synthesis through the proposed tandem Horner–Wadsworth–Emmons/oxa-Michael addition. As expected, deprotonation of **6** [31] with KHMDS at −40 °C for 0.5 h followed by the addition of the lactol **5a** led to the formation of katsumadain A as the only diastereoisomer in 52% yield, apparently via the in situ generated intermediate **8**. The spectroscopic data of the synthetic katsumadain A were in accordance with those of the natural one [32]. However, we found that its optical rotation ([a]_D_^25^ = −75.4, *c* 0.40, EtOH) was quite different from the reported one ([a]_D_^25^ +3.7, *c* 0.40, EtOH), indicating that the naturally occurring **1** might exist as a racemic substance. Given that the two enantiomers of katsumadain A might show different behaviors in the biological studies from each other as well as from the racemic compounds, we also synthesized (+)-katsumadain A in a similar way by simply replacing the catatalyst **B** with its enantiomer in the organocatalytic 1,4-conjugate addition (Scheme 3).

## Conclusion

We accomplished the first enantioselective total synthesis of katsumadain A, a naturally occurring inﬂuenza virus neuraminidase (NA) inhibitor. The key elements of the synthesis featured a bioinspired, organocatalytic enantioselective 1,4-conjuate addition and a tandem HWE/oxa-Michael addition. Due to the high efficiency and flexibility of the synthetic route it is applicable to the syntheses of both enantiomers of katsumadain A as well as their analogues. Applications of these compounds in relevant biomedical studies are ongoing in this laboratory, and the progress will be reported in due course.

## Experimental

**Representative procedure for the organocatalytic 1,4-conjugate addition**: To a mixture of **3a** (214 mg, 1.0 mmol) and **7a** (163 mg, 1.2 mmol) in dry CH_2_Cl_2_ (5 mL) at 0 °C was added PhCOOH (24 mg, 0.2 mmol) and catatalyst **B** (50 mg, 0.2 equiv). The mixture was stirred at 0 °C for 10 h before being quenched by saturated aqueous NH_4_Cl. The mixture was extracted with DCM (3 × 10 mL), and the organic layers were washed with brine and dried over MgSO_4_. The organic solvent was removed under vacuum, and the residue was purified by column chromatography (CH_2_Cl_2_:ethyl acetate = 20:1) to give **5a** (284 mg, 82% yield) as a light yellow solid.

## Supporting Information

File 1 Experimental procedures and characterization data for synthetic **1**, **3a**–**c**, **5a**–**k** and **9a**–**k**.
