# Supplementary material for: Bioinspired total synthesis of katsumadain A by organocatalytic enantioselective 1,4-conjugate addition
Source: Beilstein J Org Chem. 2013 Aug 6;9:1601–6. doi: 10.3762/bjoc.9.182 (PMC3740603; doi:10.3762/bjoc.9.182)
Supplement: File 1 — Experimental procedures and characterization data for synthetic 1, 3a–c, 5a–k and 9a–k. [file Beilstein_J_Org_Chem-09-1601-s001.pdf]

Supporting Information

for

Bioinspired total synthesis of katsumadain A via organocatalytic enantioselective 1,4-conjugate addition

Yongguang Wang, Ruiyang Bao, Shengdian Huang and Yefeng Tang*

Address: The Comprehensive AIDS Research Center, Department of Pharmacology & Pharmaceutical Sciences, School of Medicine, Tsinghua University, Beijing, 100084, China

Email: YefengTang* - yefengtang@tsinghua.edu.cn

*Corresponding author

Experimental procedures and characterization data for synthetic **1**, **3a**–**c**, **5a**–**k** and **9a**–**k**

Table of Contents

General Information…………………………………………………………… S2

Part 1: Experimental details and characteristic data…………………………. S3–S11

Part 2: NMR Spectrum…………………………………………………......... S12–S38

Part 3: HPLC chart of **9a–k**……………………………………….…................. S39–S48

General Information:

Experimental details

Unless otherwise mentioned, all reactions were carried out under a nitrogen atmosphere and anhydrous conditions and all reagents were purchased from commercial suppliers without further purification. Solvent purification was conducted according to Purification of Laboratory Chemicals [1]. Yields refer to chromatographically and spectroscopically (^{1}H NMR) homogeneous materials.

Reactions were monitored by thin-layer chromatography on plates (GF254) supplied by Yantai Chemicals (China) using UV light as visualizing agent and an ethanolic solution of phosphomolybdic acid and cerium sulfate, and heat as developing agents. If not specially mentioned, flash column chromatography uses silica gel (200–300 mesh) supplied by Tsingtao Haiyang Chemicals (China).

NMR spectra were recorded on Bruker AV400 instrument. TMS was used as internal standard for ^{1}H NMR (0 ppm), and solvent signal was used as reference for ^{13}C NMR ($CDCl_3$, 77.160 ppm). The following abbreviations were used to explain the multiplicities: s = singlet, d = doublet, t = triplet, q = quartet, br = broad, td = triple doublet, qd = quarter doublet, m = multiplet.

Enantiomeric excesses were determined by chiral HPLC analysis on CHIRALPAK-AD (Column NO. AD00CE-AJ123) in comparison with the authentic racemates. High-resolution mass spectra (HRMS) were recorded on a Bruker ESI-Q/TOF MS, low-resolution mass spectral analyses were performed with a Waters AQUITY UPLCTM/MS.

Part 1: Experimental details and characteristic data.

1. Synthesis of 3a–c

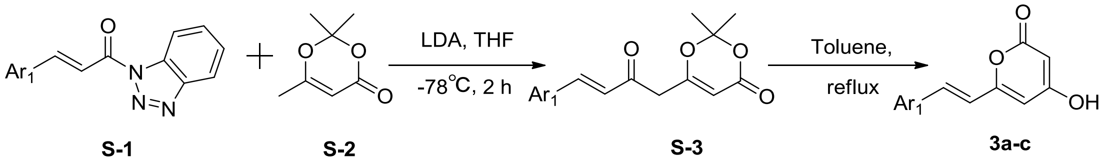

To a solution of diisopropylamine (0.77 g, 7.5 mmol) in THF (20 mL) at –78 °C was added *n*-BuLi (1.6 M, 5.16 mL, 8.25 mmol) dropwise over 30 min. A solution of 2,2,6-trimethyl-1,3-dioxin-4-one **S-2** (0.85 g, 5.68 mmol) in THF (20 mL) was then added dropwise at –78 °C for 10–15 min. After stirring for 1.5 h, a solution of **S-1** (5 mmol) in THF (20 mL) was added at –78 °C. The resulting mixture was stirred at –78 °C and allowed to warm to room temperature overnight. Saturated aqueous ammonium chloride solution (2 mL) was added to quench the reaction, and the reaction mixture was concentrated under reduced pressure. Water (100 mL) was added to the residue, and the solution was extracted with ethyl acetate (3 × 50 mL). The combined organic phase was washed with saturated aqueous sodium carbonate solution (100 mL), and dried over anhydrous $MgSO_4$. After evaporation of the solvent, the residue was purified by column chromatography on silica gel to give **S-3** [2].

A solution of **S-3** in toluene (30 mL) was heated under reflux for 20–30 min. The solvent was then removed under reduced pressure and the residue was purified by column chromatography on silica gel to give **3a–c** (45–52%) as yellow solid.

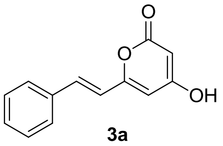

3a

Characteristic data of 3a: ^{1}H NMR (400 MHz, DMSO-d_6) δ 11.8 (s, 1H), 7.78 (d, *J* = 7.2 Hz, 2H), 7.35–7.46 (m, 3H), 7.31 (d, *J* = 16.0 Hz, 1H), 7.05 (d, *J* = 16.0 Hz, 1H), 6.25 (d, *J* = 1.6 Hz, 1H), 5.35 (d, *J* = 1.6 Hz, 1H); ^{13}C NMR (100 MHz, DMSO-d_6) δ 170.4, 163.0, 159.1, 135.1, 133.7, 129.3, 128.7, 127.4, 119.8, 102.0, 90.6; MS (ESI) *m/z* $[M+H]^+$ calcd for $C_{13}H_{10}O_3$ 215.06 , found 215.49.

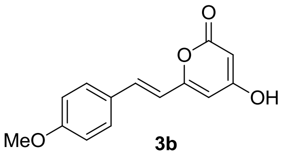

3b

Characteristic data of 3b: ^{1}H NMR (400 MHz, DMSO-d_6) δ 11.66 (s, 1H), 7.60–7.62 (m, 2H), 7.27 (d, *J* = 16.0 Hz, 1H), 6.97 (d, *J* = 16.0 Hz, 2H), 6.86 (d, *J* = 16.0 Hz, 1H), 6.16 (s, 1H), 5.30 (s, 1H), 3.79 (s, 3H); ^{13}C NMR (100 MHz, DMSO-d_6) δ 170.7, 163.4, 160.7, 160.0, 134.2, 129.5, 128.3, 117.9, 114.8, 101.3, 90.0, 55.7; MS (ESI) *m/z* $[M+H]^+$ calcd for $C_{14}H_{12}O_4$ 245.07, found 245.38.

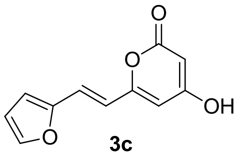

3c

Characteristic data of 3c: ^{1}H NMR (400 MHz, DMSO-d_6) δ 11.69 (s, 1H), 7.78 (d, J = 16.0 Hz, 2H), 7.14 (d, J = 16.0 Hz, 1H), 6.80 (d, J = 16.0 Hz, 2H), 6.68 (d, J = 16.0 Hz, 1H), 6.50 (m, 1H), 6.26 (s, 1H), 5.30 (s, 1H); ^{13}C NMR (100 MHz, DMSO-d_6): 159.2, 151.7, 145.3, 121.7, 117.5, 114.1, 113.1, 102.2, 90.3; MS (ESI) m/z [M+H]$^+$ calcd for $C_{11}H_8O_4$ 205.04, found 205.48.

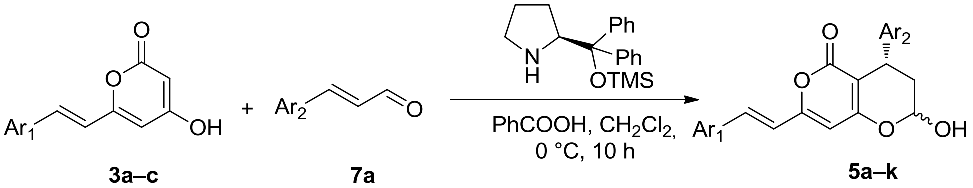

Synthesis of compound 5a–k: To a mixture of **3a** (214 mg, 1.0 mmol) and **7a** (163 mg, 1.2 mmol) in dry CH_2Cl_2 (5 mL) at 0 °C was added PhCOOH (24 mg, 0.2 mmol) and cat. B [3] (50 mg, 0.2 equiv). The mixture was stirred at 0 °C for 10 h before quenched by saturated aqueous NH_4Cl. The mixture was extracted with DCM (3 × 10 mL), and the organic layers were washed with brine and dried over $MgSO_4$. The organic solvent was removed in vacuo and the residue was purified by column chromatography (CH_2Cl_2:ethyl acetate = 20:1) to give **5a** (284 mg, 82% yield) as light yellow solid. **5a–k** was obtained as a mixture of diastereoisomer, and only the major isomer was documented in the following data.

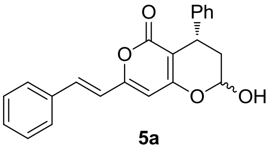

5a

Characteristic data of 5a: ^{1}H NMR (400 MHz, DMSO-d_6) δ 7.04–7.94 (m, 11H), 6.39 (s, 1H), 5.19–5.22 (m, 1H), 4.01–4.04 (m, 1H), 2.02–2.07 (m, 2H); ^{13}C NMR (100 MHz, DMSO-d_6) δ 167.7, 164.8, 162.1, 158.0, 144.2, 135.7, 133.9, 133.3, 129.7, 129.3, 129.0, 128.8, 127.8, 126.8, 120.2, 101.8, 100.9, 93.7, 37.1, 35.5; HRMS (ESI) m/z [M+H]$^+$ calcd for $C_{22}H_{18}O_4$ 347.1278, found 347.1272.

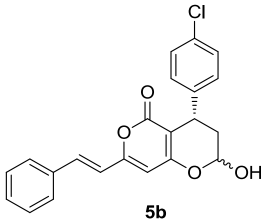

5b

Characteristic data of 5b: ^{1}H NMR (400 MHz, acetone-d_6) δ 7.65–7.74 (m, 2H), 7.23–7.44 (m, 8H), 7.04–7.08 (m, 1H)，6.37 (s, 1H), 5.22 (s, 1H), 4.01–4.04 (m, 1H), 1.99–2.13 (m, 2H). ^{13}C NMR (100 MHz, acetone-d_6) δ 164.1, 158.6, 156.8, 142.9, 133.8, 129.8, 129.3, 128.7, 127.9, 120.1, 101.3, 99.9, 92.3, 49.0, 36.6, 34.7; HRMS (ESI) m/z [M+H]$^+$ calcd for $C_{22}H_{17}ClO_4$ 381.0888, found 381.0879.

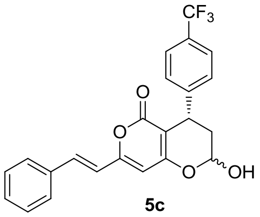

5c

Characteristic data of 5c: ^{1}H NMR (400 MHz, acetone-d_6) δ 7.52–7.68 (m, 4H), 7.36–7.50 (m, 7H), 6.97–7.01 (m, 1H), 6.27 (s, 1H), 5.44–5.46 (m, 1H), 4.21–4.23 (m, 1H), 2.18–2.32 (m, 2H); ^{13}C NMR (100 MHz, acetone-d_6) δ 164.2, 161.9, 158.5, 135.5, 134.1, 129.1, 128.8, 128.3, 127.9, 127.4, 124.8, 119.3, 101.6, 101.3, 100.2, 93.0, 36.3, 35.2; HRMS (ESI) *m/z* $[M+H]^+$ calcd for $C_{23}H_{17}F_3O_4$ 415.1152, found 415.1154.

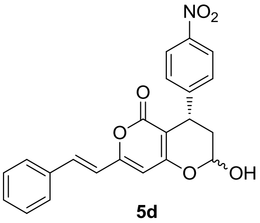

5d

Characteristic data of 5d: ^{1}H NMR (400 MHz, acetone-d_6) δ 8.10–8.17 (m, 2H), 7.66–7.68 (m, 3H), 7.28–7.42 (m, 6H), 7.07–7.149 (m, 1H), 6.40 (s, 1H), 5.29 (s, 1H), 4.13–4.16 (m, 1H), 2.01–2.21 (m, 2H); ^{13}C NMR (100 MHz, acetone-d_6) δ 164.9, 162.0, 158.3, 152.4, 146.58, 135.6, 134.1, 129.3, 129.2, 127.9, 124.0, 120.1, 102.0, 100.3, 93.3, 36.7, 35.0; HRMS (ESI) *m/z* $[M+H]^+$ calcd for $C_{22}H_{17}NO_6$ 392.1129, found 392.1122.

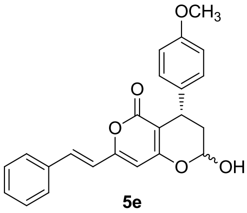

5e

Characteristic data of 5e: ^{1}H NMR (400 MHz, acetone-d_6) δ 7.68–7.86 (m, 2H), 7.36–7.66 (m, 4H), 7.14–7.16 (m, 2H)， 6.87–6.99 (m, 5H), 6.24 (s, 1H), 5.36–5.39 (m, 1H), 4.07–4.09 (m, 1H), 3.77 (s, 1H), 2.06–2.19 (m, 2H); ^{13}C NMR (100 MHz, acetone-d_6) δ 164.2, 161.1, 158.6, 157.6, 136.1, 135.4, 133.7, 129.2, 128.8, 128.3, 127.1, 119.6, 113.4, 101.3, 93.0, 54.2, 37.0, 34.6; HRMS (ESI) *m/z* $[M+H]^+$ calcd for $C_{23}H_{20}O_5$ 377.1384, found 377.1385

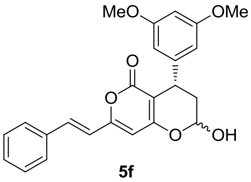

5f

Characteristic data of 5f: ^{1}H NMR (400 MHz, acetone-d_6) δ 7.66–7.68 (m, 2H), 7.37–7.45 (m, 3H), 6.95–6.99 (m, 1H), 6.37–6.40 (m, 3H), 6.14 (s, 1H), 5.39–5.42 (m, 1H), 4.05–4.08 (m, 1H), 3.74 (s, 6H), 2.20–2.22 (m, 2H); ^{13}C NMR (100 MHz, acetone-d_6) δ 164.2, 161.4, 160.5, 157.8, 146.3, 136.2, 133.6, 128.6, 128.0, 127.2, 119.3, 105.1, 101.6, 100.5, 97.2, 93.2, 54.3, 36.3, 35.8; HRMS (ESI) *m/z* $[M+H]^+$ calcd for $C_{24}H_{22}O_6$ 407.1489, found 407.1481.

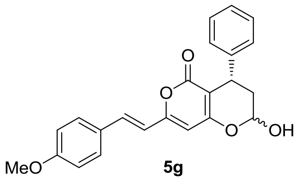

5g

Characteristic data of 5g: ^{1}H NMR (400 MHz, acetone-d_6) δ 7.61–7.63 (m, 2H), 7.23–7.26 (m, 5H), 6.81–6.99 (m, 2H), 6.76–6.80 (m, 1H), 6.18 (s, 1H), 5.32–5.36 (m, 1H), 4.11–4.13 (s, 1H), 3.24 (s, 3H), 2.19–2.22 (m, 2H), ^{13}C NMR (100 MHz, acetone-d_6) δ 165.1, 162.5, 161.4, 158.8, 144.5, 134.1, 128.9, 128.3 (128.3), 127.5, 126.3, 117.3, 114.4, 100.5, 92.9, 54.7, 37.0, 34.9; HRMS (ESI) *m/z* $[M+H]^+$ calcd for $C_{23}H_{20}O_5$ 377.1384, found 377.1385.

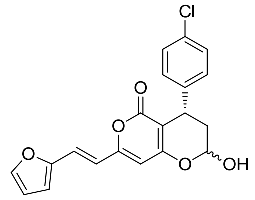

5h

Characteristic data of 5h: ^{1}H NMR (400 MHz, acetone-d_6) δ 7.72–7.80 (m, 2H), 7.20–7.36 (m, 5H), 6.61–6.74 (m, 3H)，6.44 (s, 1H), 5.21 (s, 1H), 4.00 (s, 1H), 1.99–2.11 (m, 2H); ^{13}C NMR (100 MHz, acetone-d_6) δ 165.1, 162.5, 157.0, 151.2, 144.9, 143.1, 131.5, 128.9, 128.3, 121.3, 117.9, 114.4, 113.3, 101.9, 100.2, 92.9, 37.2, 34.7; HRMS (ESI) *m/z* $[M+H]^+$ calcd for $C_{20}H_{15}ClO_5$ 371.0681, found 371.0681.

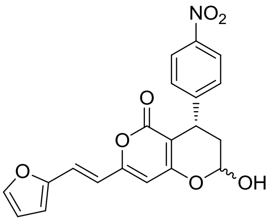

5i

Characteristic data of 5i: ^{1}H NMR (400 MHz, acetone-d_6) δ 8.20 (d, J = 12.0 Hz 2H), 7.68 (s, 1H), 7.58 (d, J = 8.0 Hz, 2H), 7.18 (d, J = 12.0 Hz, 1H), 6.58–6.76 (m, 3H), 6.29 (s, 1H), 5.50 (m, 1H), 4.24 (m, 1H), 2.17–2.33 (m, 3H); ^{13}C NMR (100 MHz, acetone-d_6) δ 164.5, 161.3, 158.0, 149.0, 135.5, 133.9, 129.3, 128.7, 128.2, 127.3, 125.3, 119.5, 101.3, 100.5, 93.0, 36.6, 35.1; HRMS (ESI) *m/z* $[M+H]^+$ calcd for $C_{20}H_{15}NO_7$ 382.0921, found 382.0921.

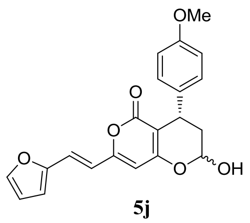

5j

Characteristic data of 5j: ^{1}H NMR (400 MHz, acetone-d_6) δ 7.66–7.68 (m, 1H), 7.14–7.16 (m, 3H), 6.75–6.99 (m, 5H)，6.26 (s, 1H), 5.34 (s, 1H), 4.06–4.07 (s, 1H), 3.78 (s, 3H), 2.16–2.18 (m, 2H); ^{13}C NMR (100 MHz, acetone-d_6); δ 164.3, 161.4, 158.5, 157.0, 151.5, 143.5, 134.9, 128.6, 121.3, 116.4, 113.5, 112.6, 112.0, 100.2, 93.1, 54.3, 37.0, 34.6; HRMS (ESI) *m/z* $[M+H]^+$ calcd for $C_{21}H_{18}O_6$ 367.1176, found 367.1173.

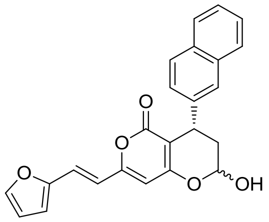

5k

Characteristic data of 5k: ^{1}H NMR (400 MHz, acetone-d_6) δ 7.84–7.90 (m, 5H), 7.45–7.50 (m, 3H), 7.17–7.21 (m, 1H)， 6.59–6.70 (m, 3H), 6.31 (s, 1H), 5.43–5.55 (m, 1H), 4.28–4.30 (s, 1H), 2.28–2.32 (m, 2H); ^{13}C NMR (100 MHz, acetone-d_6) δ 164.3, 161.8, 157.9, 151.9, 144.4, 142.0, 133.4, 132.4, 128.1, 127.6, 127.4, 126.3, 125.7, 125.3, 116.7, 112.6, 111.8, 101.0, 100.5, 92.6, 36.7, 35.2; HRMS (ESI) *m/z* [M+H]$^+$ calcd for $C_{24}H_{18}O_5$ 387.1227, found 387.1227.

Determination of the absolute configuration of 5a [4]

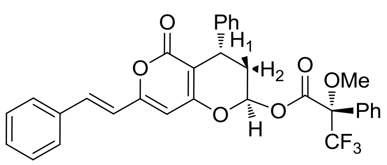

(S)-Mosher ester
S-5

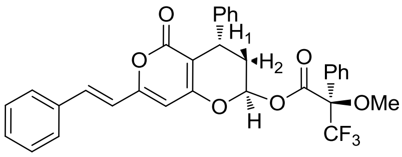

(R)-Mosher ester
S-6

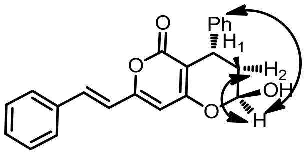

NOE-analysis

H	δ [ppm], (*S*)-Mosher ester	δ [ppm], (*R*)-Mosher ester	Δδ= δS − δR
H_1	2.36 – 2.43 (2.395)	2.31 – 2.38 (2.345)	0.05
H_2	2.23 – 2.28 (2.255)	2.16 – 2.22 (2.19)	0.065

Synthesis of compound S-5 and S-6: Oxalyl chloride (12 mg, 0.057 mmol) was added to a solution of (R)-MTPA (15 mg, 0.043 mmol) and DMF (0.9 mg, 0.012 mmol) in DCM. After 1 h the mixture was filtered and concentrated. A solution of **5a** in DCM (0.5 mL), Et_3N (14 mg, 0.13 mmol), and DMAP (a small crystal, ca. 1 mg) was added to the residue, and then stirred at rt for 1 h. The mixture was purified by column chromatography (CH_2Cl_2:ethyl acetate = 20:1) on silica gel to give **S-5** and **S-6**.

Characteristic data of S-5: ^{1}H NMR (400 MHz, $CDCl_3$) δ 7.20–7.50 (m, 10 H), 6.60 (d, *J* = 16.0 Hz, 1H), 6.48 (d, *J* = 4.0 Hz, 1H), 5.99 (s, 1H), 4.07 (m, 1H), 3.55 (s, 3H), 2.40 (m, 1H), 2.26 (dd, *J* = 12.0, 4.0 Hz, 1H).

Characteristic data of S-6: ^{1}H NMR (400 MHz, $CDCl_3$) δ 7.15–7.50 (m, 10 H), 6.62 (d, *J* = 16.0 Hz, 2H), 6.48 (dd, *J* = 8.0, 4.0 Hz, 1H), 6.04 (s, 1H), 4.04 (m, 1H), 3.55 (s, 2H), 2.33 (m, 1H), 2.20 (dd, *J* = 12.0, 8.0 Hz, 1H).

5a–k was isolated as a mixture of **C-5** diastereoisomers (β-isomer/α-isomer = 5/1 to 7/1) in all of the above cases. For convenience, the ee value of **5a–k** was determined with the corresponding lactone derivative **9a–k**.

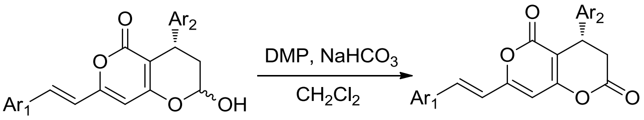

5a–k **9a–k**

Synthesis of compound 9a–k: To a solution (0.02 M) of **5a–k** (1.0 equiv) in DCM was added DMP (1.5 equiv) and $NaHCO_3$. The resulting solution was stirred at room temperature for 4 h and diluted with ether then filtered. The solvent was removed in vacuo and the residue was subjected to column chromatography (CH_2Cl_2) to get the product.

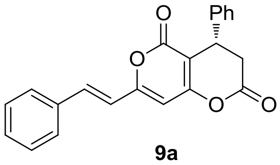

9a

Characteristic data of 9a: ^{1}H NMR (400 MHz, $CDCl_3$) δ 7.56–7.6 (m, 3H), 7.25–7.44(m, 10H), 6.65–6.69 (m, 1H), 6.17 (s, 1H), 4.44–4.46 (m, 1H), 3.12–3.14 (m, 2H); ^{13}C NMR (100 MHz, $CDCl_3$) δ 164.6, 161.6, 161.5, 159.7, 139.5, 136.8, 134.9, 129.8, 129.2, 128.9, 127.9, 127.5, 126.5, 118.1, 104.6, 99.3, 36.0, 35.5; HRMS (ESI) *m*/*z* $[M+H]^+$ calcd for $C_{22}H_{16}O_4$ 367.0941, found 367.0942.

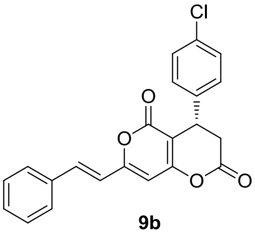

9b

Characteristic data of 9b: ^{1}H NMR (400 MHz, $CDCl_3$) δ 7.53–7.56 (m, 3H), 7.37–7.52 (m, 3H), 7.29–7.31 (m, 2H), 7.17–7.19 (m, 2H), 6.65 (d, J = 16.0 Hz, 1H), 6.15 (s, 1H), 4.39–4.41 (m, 1H), 3.02–3.16 (m, 2H); ^{13}C NMR (100 MHz, $CDCl_3$) δ 164.2, 161.7, 161.4, 159.9, 138.1, 137.13, 134.1, 133.9, 129.9, 129.3, 129.0, 127.9, 127.6, 118.0, 104.1, 99.1, 35.8, 34.9; HRMS (ESI) *m*/*z* $[M+H]^+$ calcd for $C_{22}H_{15}ClO_4$ 379.0732, found 379.0721.

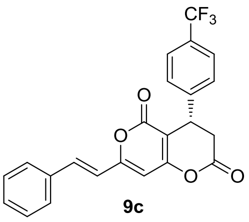

9c

Characteristic data of 9c: ^{1}H NMR (400 MHz, $CDCl_3$) δ 7.50–7.61 (m, 5H), 7.36–7.41 (m, 5H), 6.64–6.67 (m, 3H), 6.16 (s, 1H), 4.48–4.49 (m, 1H), 3.06–3.22 (m, 2H); ^{13}C NMR (100 MHz, $CDCl_3$) δ 164.1, 161.4, 157.1, 137.8, 134.7, 130.0, 129.0, 127.7, 127.1, 117.9, 102.9, 97.8, 35.6, 35.3; HRMS (ESI) *m*/*z* $[M+H]^+$ calcd for $C_{23}H_{15}F_3O_4$ 411.0839, found 411.0994.

S8

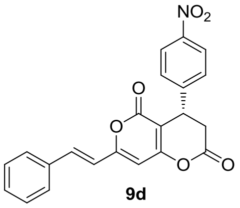

9d

Characteristic data of 9d: ^{1}H NMR (400 MHz, $CDCl_3$) δ 8.19–8.21 (m, 2H), 7.52–7.54 (m, 3H), 7.39–7.43 (m, 5H), 6.66 (d, *J* = 16.0 Hz, 1H), 6.17 (s, 1H), 4.52–4.54 (m, 1H), 3.07–3.23 (m, 2H); ^{13}C NMR (100 MHz, $CDCl_3$) δ 163.7, 162.2, 161.3, 160.4, 147.3, 146.8, 137.6, 134.6, 130.1, 129.0, 127.7, 126.6, 124.4, 117.8, 103.0, 35.3; HRMS (ESI) *m/z* $[M+H]^+$ calcd for $C_{22}H_{15}NO_6$ 390.0972, found 390.0971.

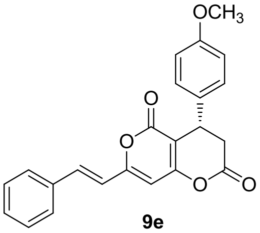

9e

Characteristic data of 9e: ^{1}H NMR (400 MHz, $CDCl_3$) δ 7.51–7.55 (m, 3H), 7.38–7.40 (m, 3H), 6.64 (d, *J* = 16.0 Hz, 2H), 6.35–6.37 (m, 3H), 6.13 (s, 1H), 4.36 (s, 1H), 3.07–3.08 (m, 2H); ^{13}C NMR (100 MHz, $CDCl_3$) δ 164.5, 161.8, 161.0, 161.9, 159.8, 141.9, 136.9, 134.8, 129.0, 129.0, 127.6, 118.0, 104.8, 103.9, 99.4, 99.2, 36.3, 35.2; HRMS (ESI) *m/z* $[M+H]^+$ calcd for $C_{23}H_{18}O_5$ 375.1227, found 375.1220.

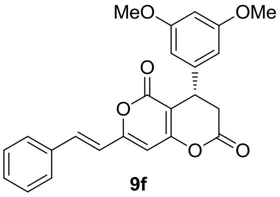

9f

Characteristic data of 9f: ^{1}H NMR (400 MHz, $CDCl_3$) δ 7.51–7.55 (m, 3H), 7.38–7.40 (m, 3H), 6.64 (d, *J* = 16.0 Hz, 2H), 6.35–6.37 (m, 3H), 6.13 (s, 1H), 4.36 (s, 1H), 3.07–3.08 (m, 2H); ^{13}C NMR (100 MHz, $CDCl_3$) δ 164.5, 161.8, 161.0, 161.3, 159.8, 141.9, 136.9, 134.8, 129.0, 129.0, 127.6, 118.0, 104.8, 103.9, 99.4, 99.2, 36.3, 35.2; HRMS (ESI) *m/z* $[M+H]^+$ calcd for $C_{24}H_{20}O_6$ 405.1333, found 405.1334.

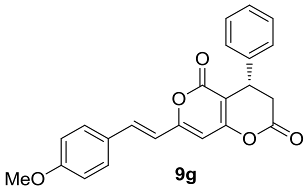

9g

Characteristic data of 9g: ^{1}H NMR (400 MHz, $CDCl_3$) δ 7.47–7.51 (m, 3H), 7.28–7.32 (m, 2H), 7.22–7.26 (m, 2H), 6.90–6.92 (m, 2H), 6.49–6.53 (m, 1H), 6.08 (s, 1H), 4.41–4.42 (m, 1H), 3.84 (s, 3H), 3.05–3.22 (m, 2H); ^{13}C NMR (100 MHz, $CDCl_3$) δ 164.7, 162.2, 161.1, 160.3, 137.4, 135.15, 129.2, 128.7, 128.6,126.60, 117.2, 115.3, 103.9, 98.0, 61.7, 36.1, 35.4; HRMS (ESI) *m/z* $[M+H]^+$ calcd for $C_{23}H_{18}O_5$ 375.1227, found 375.1220.

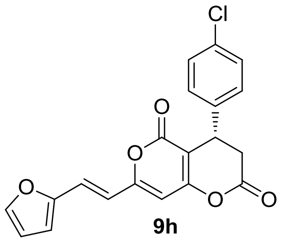

9h

Characteristic data of 9h: ^{1}H NMR (400 MHz, $CDCl_3$) δ 7.48–7.49 (m, 1H), 7.26–7.33 (m, 4H), 7.16–7.18 (m, 2H), 6.57–6.59 (m, 1H), 6.48–6.53 (m, 1H), 6.10 (s, 1H), 4.37–4.39 (m, 1H), 3.10–3.14 (m, 2H); ^{13}C NMR (100 MHz, $CDCl_3$) δ 164.3, 161.8, 161.4, 159.8, 151.3, 144.5, 138.7, 133.8, 129.3, 128.0, 123.5, 115.8, 114.4, 112.5, 103.8, 99.0, 33.8, 34.9; HRMS (ESI) *m/z* $[M+Na]^+$ calcd for $C_{20}H_{13}ClO_5$ 391.0344, found 391.0342.

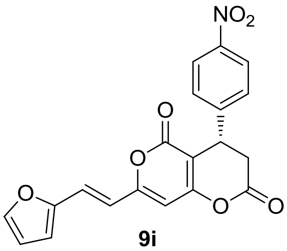

9i

Characteristic data of 9i: ^{1}H NMR (400 MHz, $CDCl_3$) δ 8.20 (d, *J* = 12.0 Hz, 2H), 7.42–7.49 (m, 3H), 7.28–7.31 (m, 1H), 6.49 - 6.59 (m, 3H), 4.52 (d, *J* = 8.0 Hz, 1H), 3.06–3.22 (m, 2H); ^{13}C NMR (100 MHz, $CDCl_3$) δ 163.7, 166.2, 161.3, 160.3, 151.2, 147.5, 146.9, 144.7, 127.7, 124.4, 123.9, 115.6, 114.7, 112.6, 102.6, 98.8, 35.3 (35.3); HRMS (ESI) *m/z* $[M+H]^+$ calcd for $C_{20}H_{13}NO_7$ 380.0765, found 380.0763.

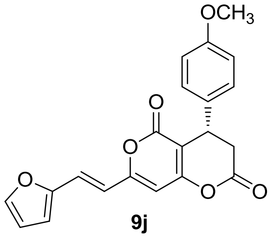

9j

Characteristic data of 9j: ^{1}H NMR (400 MHz, $CDCl_3$) δ 7.47–7.48 (m, 1H), 7.26–7.28 (m, 1H), 7.13–7.16 (m, 2H), 6.83–6.85 (m, 2H), 6.48–6.56 (m, 3H), 6.10 (s, 1H), 4.35–4.37 (m, 1H), 3.06–3.07 (m, 2H); ^{13}C NMR (100 MHz, $CDCl_3$) δ 164.3, 161.8, 161.4, 159.8, 151.3, 144.5, 138.1, 133.8, 129.4, 128.0, 123.5, 115.8, 114.0, 112.5, 103.8, 99.0, 35.8, 34.9; HRMS (ESI) *m/z* $[M+H]^+$ calcd for $C_{21}H_{16}O_6$ 365.1020, found 365.1019.

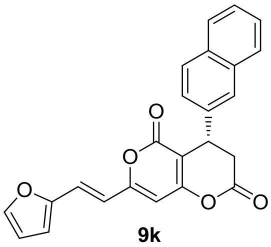

9k

Characteristic data of 9k: ^{1}H NMR (400 MHz, $CDCl_3$) δ 7.78–7.83 (m, 3H), 7.78 (s, 1H), 7.45–7.48 (m, 3H), 7.37 (d, *J* = 8.4 Hz, 1H), 7.25–7.27 (m, 1H), 6.48–6.59 (m, 3H), 6.14 (s, 1H), 4.56–4.58 (m, 1H), 3.17–3.18 (m, 2H); ^{13}C NMR (100 MHz, $CDCl_3$) δ 164.5, 161.8, 161.6, 161.3,

159.7, 141.9, 136.9, 134.9, 129.8, 129.0, 127.6, 118.1, 104.8, 104.3, 99.4, 99.3, 60.2，36.02，35.6; HRMS (ESI) *m*/*z* $[M+H]^+$ calcd for $C_{24}H_{16}O_5$ 385.1071, found 385.1062.

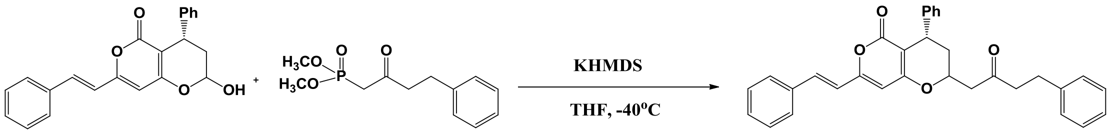

5a **6** **1**

Synthesis of compound (−)-katsumadain A: To a solution of **6** [5] (1.0 mmol) in 20 mL dry THF cooled at under N_2, was added KHMDS (1.2 mL, 1 M solution in THF). The reaction mixture was stirred 1 h at −40 °C, then added the solution of **5a** in 10 mL dry THF and stirred for another 2 h, The reaction was quenched by saturated aqueous NH_4Cl, and extracted with EA. The organic layers were collected, washed with brine, dried over by $MgSO_4$, filtered and purified by column chromatography to give katsumadain A (52%) as yellow solid. The ee value of synthetic (−)-**1** was determined to be 80%.

Characteristic data of (−)-katsumadain A: mp 71–72 °C; $[a]_D^{25}$ = −75.4 (*c* = 0.4, MeOH); IR ν_{max} (film): 1712, 1609, 1565, 1263, 1105, 811, 733 cm^{-1}; ^{1}H NMR (400 MHz, $CDCl_3$) δ 7.16–7.51 (m, 16H), 6.58 (d, *J* = 16.0 Hz, 1H), 5.93 (s, 1H), 4.45–4.52 (m, 1H), 4.13 (t, *J* = 4.0 Hz, 1H), 2.90 (t, *J* = 7.0 Hz, 2H), 2.83 (dd, *J* = 16.0, 8.0 Hz, 2H) 2.78 (t, *J* = 7.0 Hz, 2H), 2.50 (dd, *J* = 16.0, 4.0 Hz, 1H), 1.99–2.02 (m, 2H); ^{13}C NMR (100 MHz, $CDCl_3$) δ 206.4, 165.5, 163.4, 158.1, 143.5, 141.1, 135.8, 135.6, 129.3, 129.1, 128.9, 128.7, 128.1, 127.8, 127.2, 126.6, 119.1, 101.4, 101.3, 69.9, 48.0, 45.6, 35.4 (35.4), 29.8; HRMS (ESI) *m*/*z* $[M+Na]^+$ calcd for $C_{32}H_{28}O_4$ 499.1880, found 499.1887;

References

[1] Peerrin, D. D.; Armarego, W. L. and Perrins, D. R. Purification of laboratory chemicals. **1980**, Pergamon Press: Oxford.

[2] Alan R. Katritzky and et al. Facile syntheses of 2,2-dimethyl-6- (2-oxoalkyl) -1,3-dioxin-4-onesand the corresponding 6-substituted4-hydroxy-2-pyrones. *J. Org. Chem.* **2005**, *70*, 4854–4856.

[3] San N. Khong and Ohyun Kwon. Chiral aminophosphines as catalysts for enantioselective double-Michael indoline syntheses. Molecules. **2012**, *17*, 5626–5650.

[4] Hiroshi Kakisawa and *et. al.* High-field FT NMR application of mosher's method the absolute configurations of marine terpenoids. *J. Am. Chem. Soc.* **1991**, *113*, 4092–4096.

[5] Kevin M. Maloney and John Y. L. Chung. A general procedure for the preparation of β-ketophosphonates. *J. Org. Chem.* **2009**, *74*, 7574–7576.

Part 2: NMR Spectrum

^{1}H NMR Spectrum for **3b** (DMSO-d_6, 400 MHz)

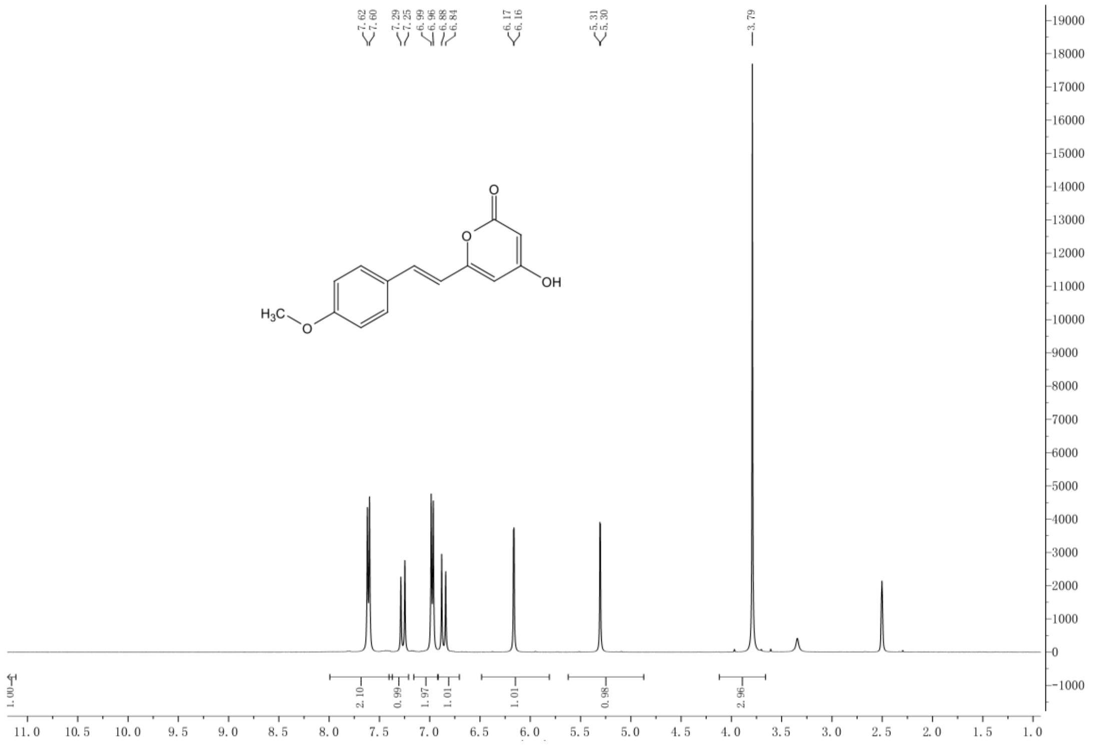

^{13}C NMR Spectrum for **3b** (DMSO-d_6, 101 MHz)

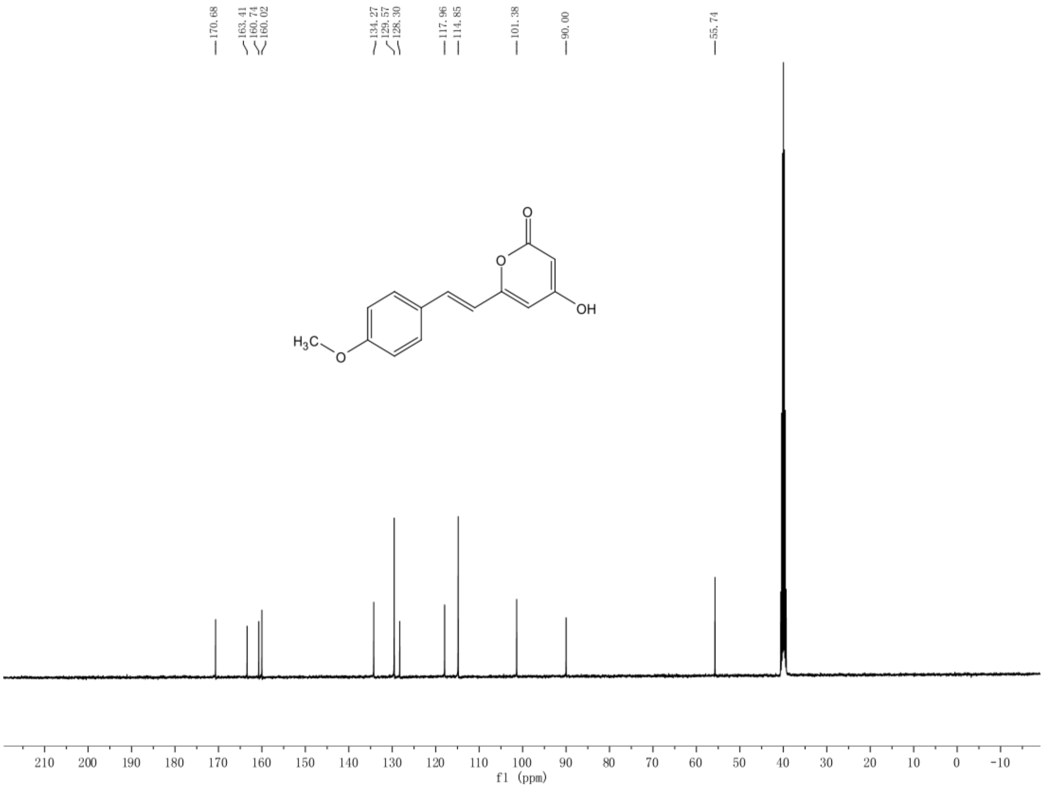

^{1}H NMR Spectrum for **3c** (DMSO-d_6, 400 MHz)

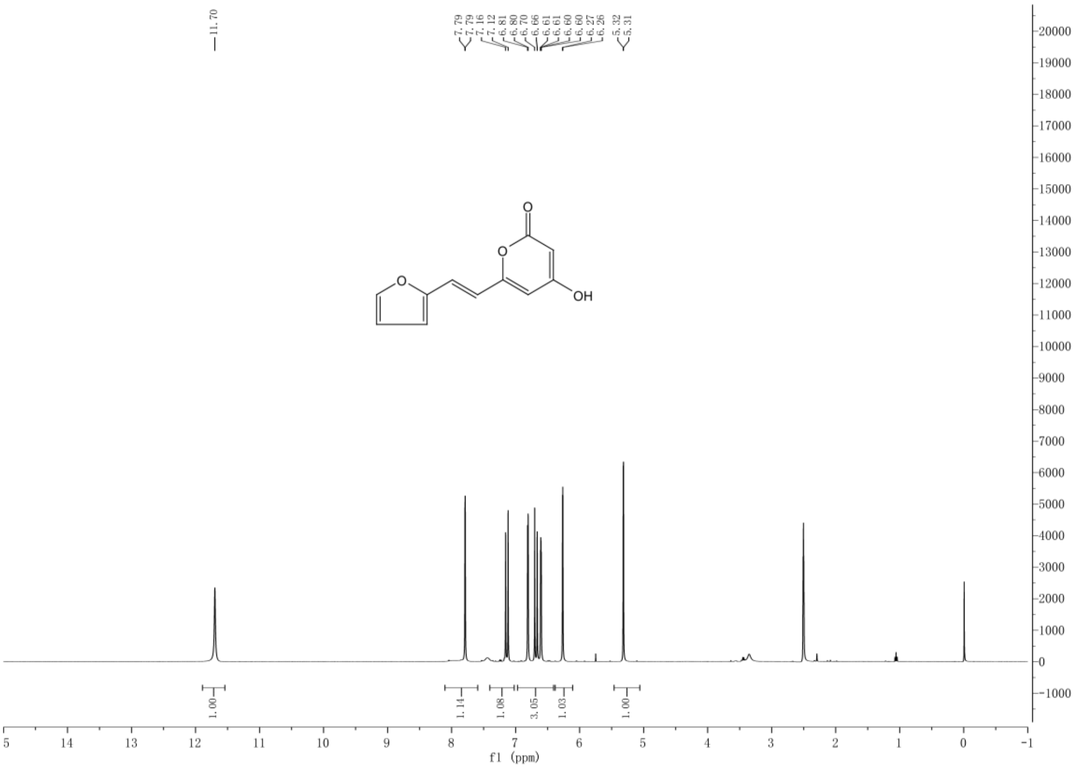

^{13}C NMR Spectrum for **3c** (DMSO-d_6, 101 MHz)

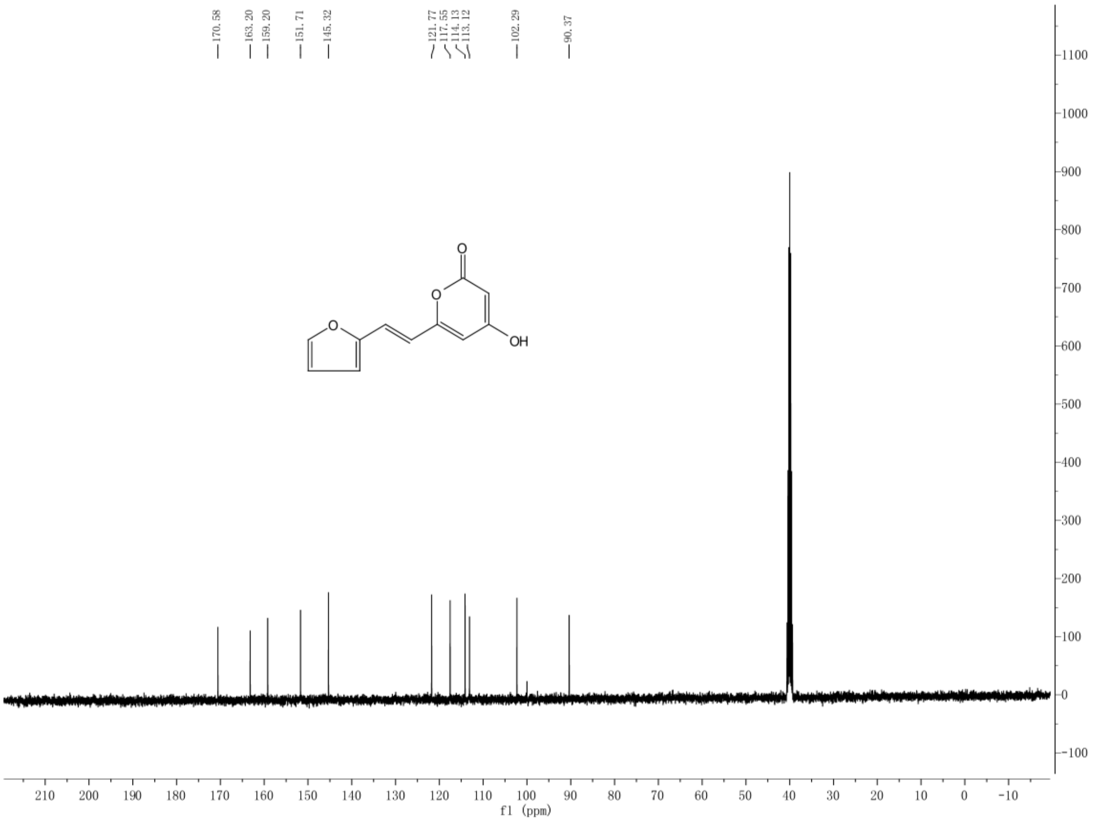

^{1}H NMR Spectrum for **5a** (DMSO-d_6, 400 MHz)

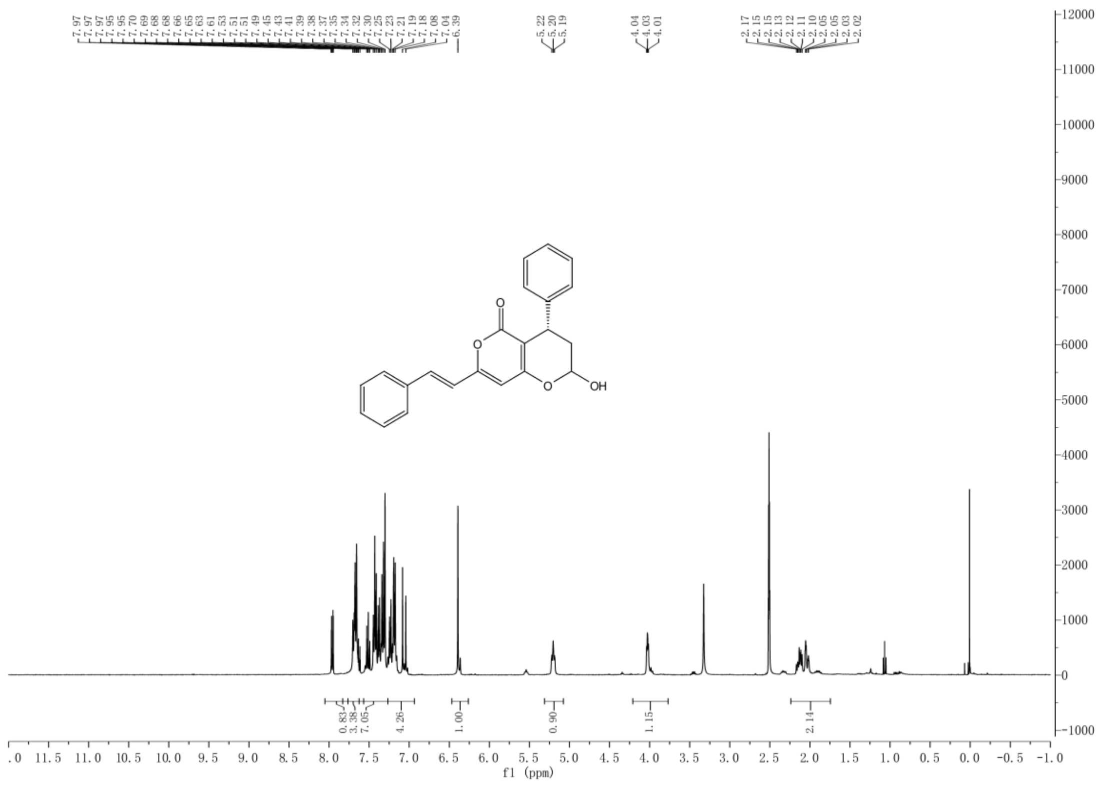

^{13}C NMR Spectrum for **5a** (DMSO-d_6, 101 MHz)

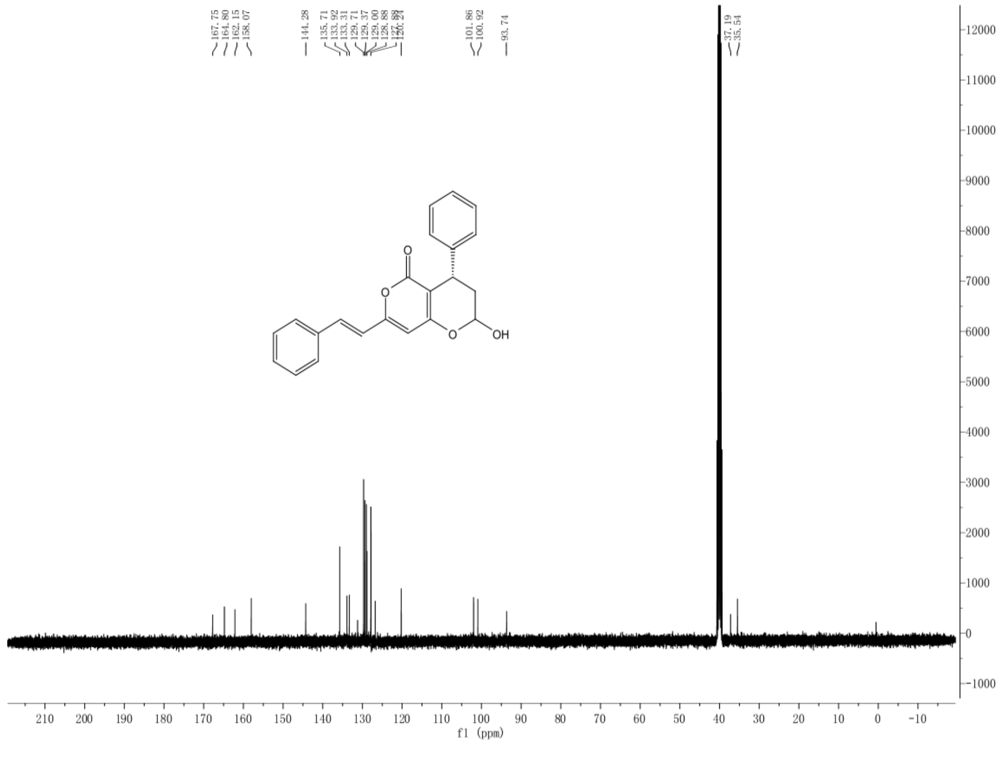

^{1}H NMR Spectrum for **5b** (acetone-d_6, 400 MHz)

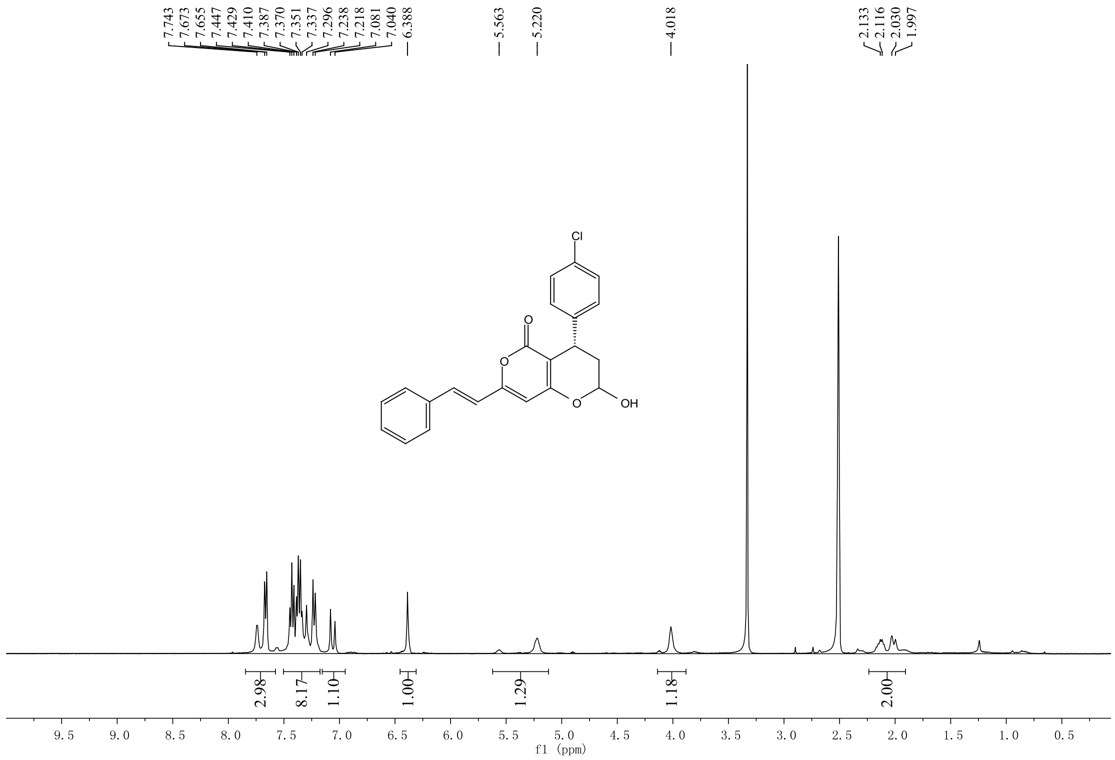

^{13}C NMR Spectrum for **5b** (acetone-d_6, 101 MHz)

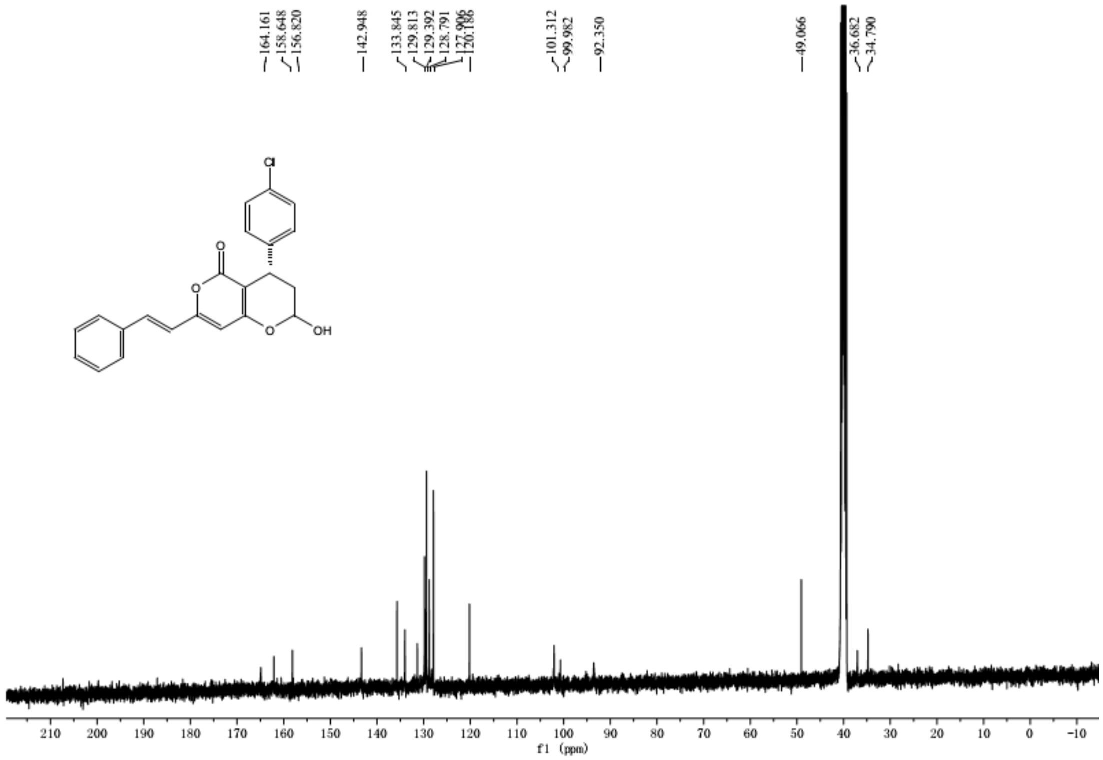

^{1}H NMR Spectrum for **5c** (acetone-d_6, 400 MHz)

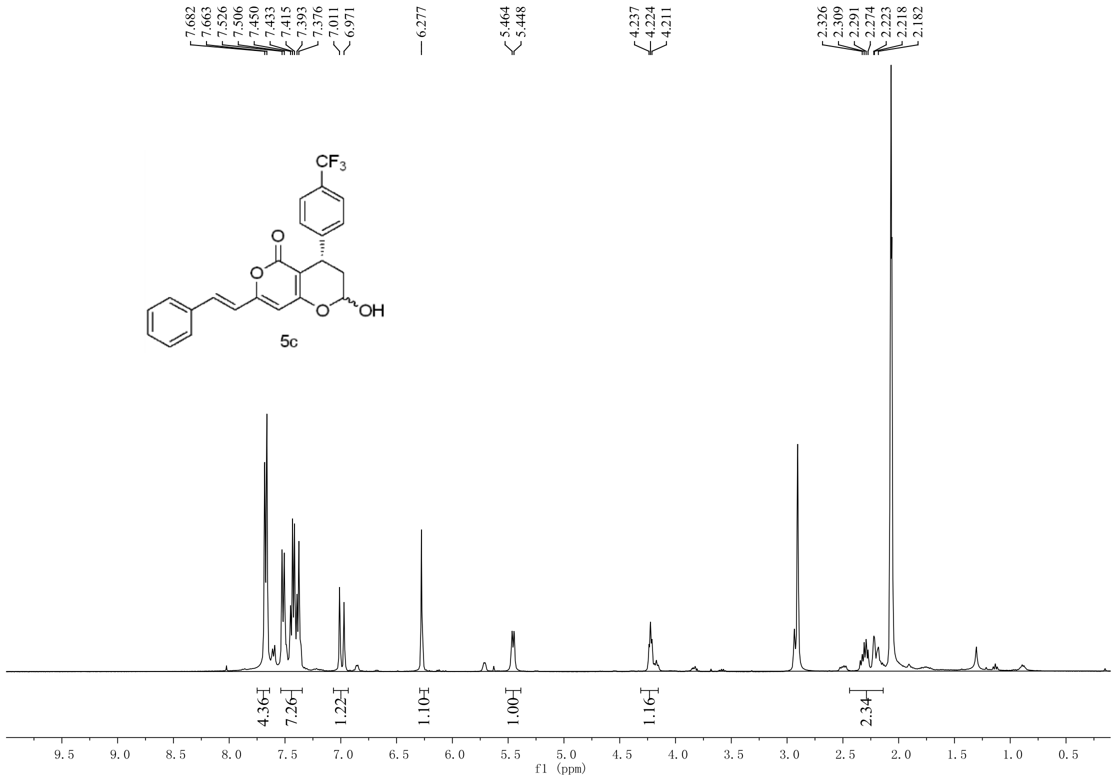

^{13}C NMR Spectrum for **5c** (acetone-d_6, 101 MHz)

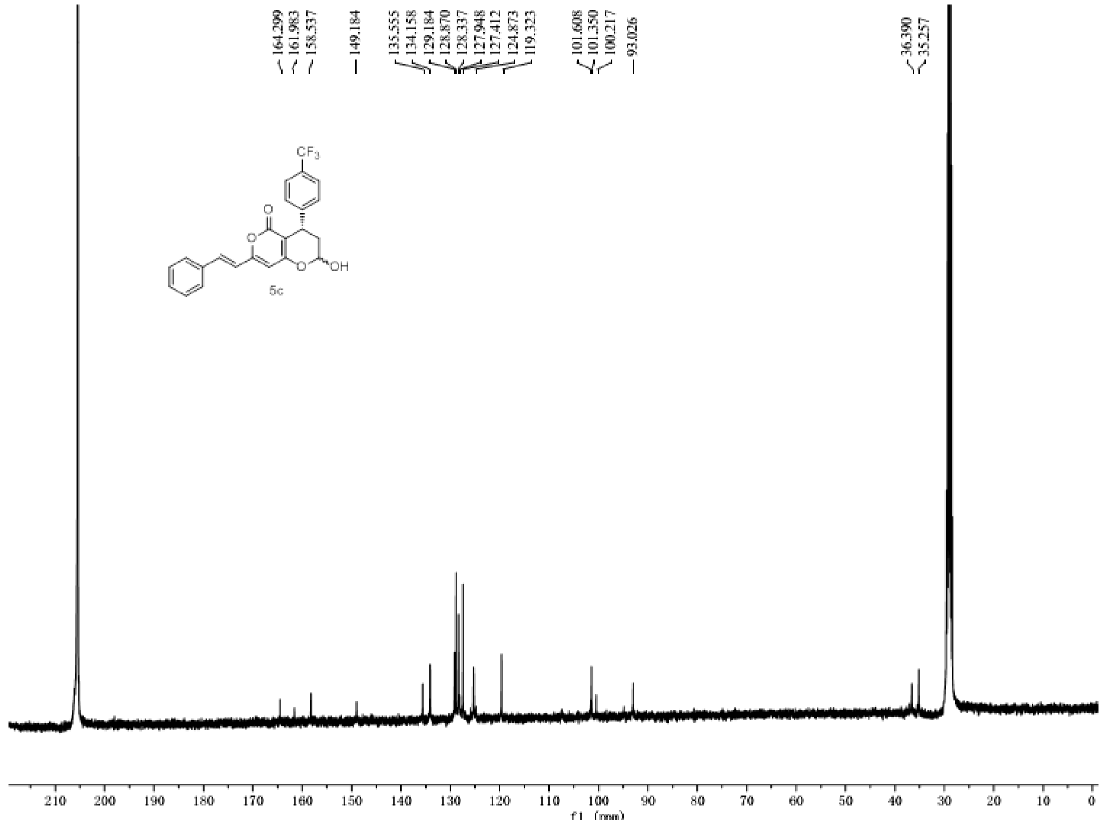

^{1}H NMR Spectrum for **5d** (acetone-d_6, 400 MHz)

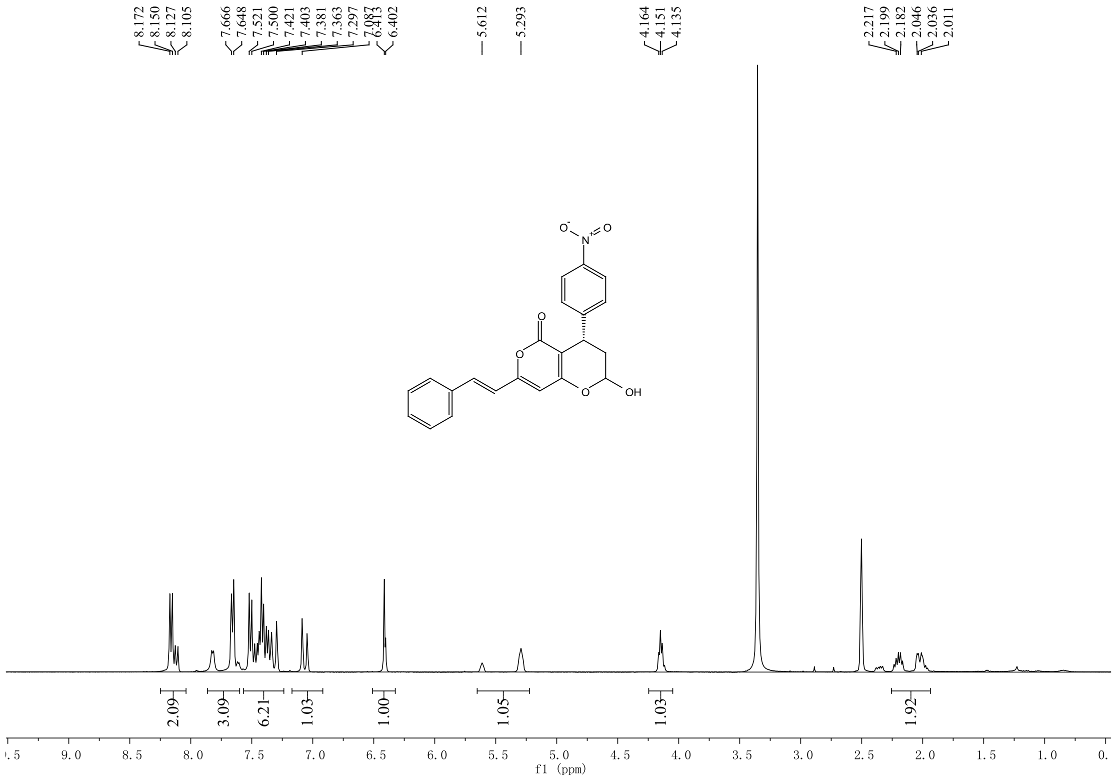

^{13}C NMR Spectrum for **5d** (acetone-d_6, 101 MHz)

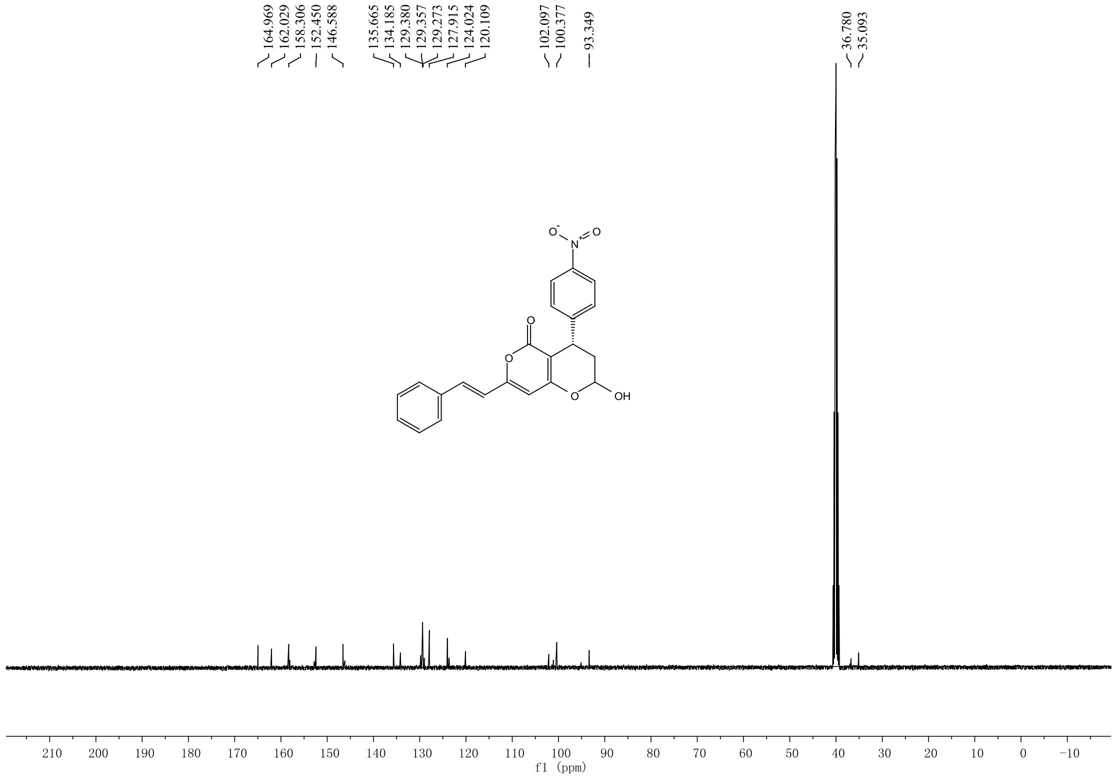

^{1}H NMR Spectrum for **5e** (acetone-d_6, 400 MHz)

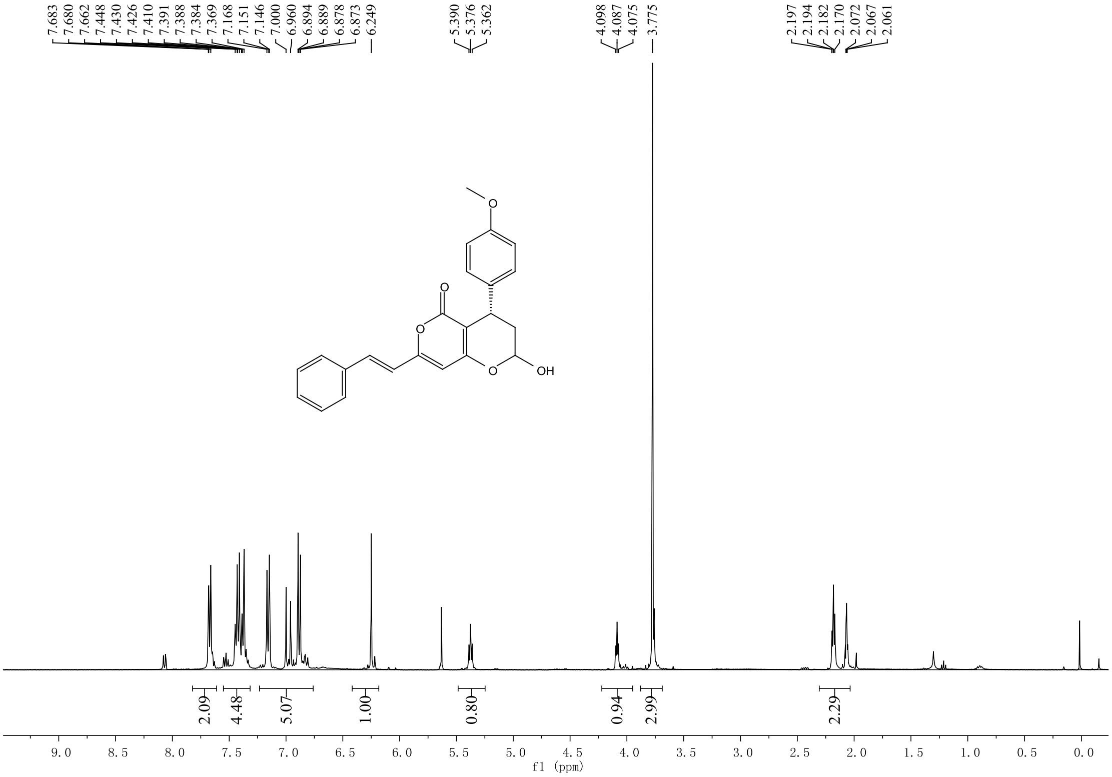

^{13}C NMR Spectrum for **5e** (acetone-d_6, 101 MHz)

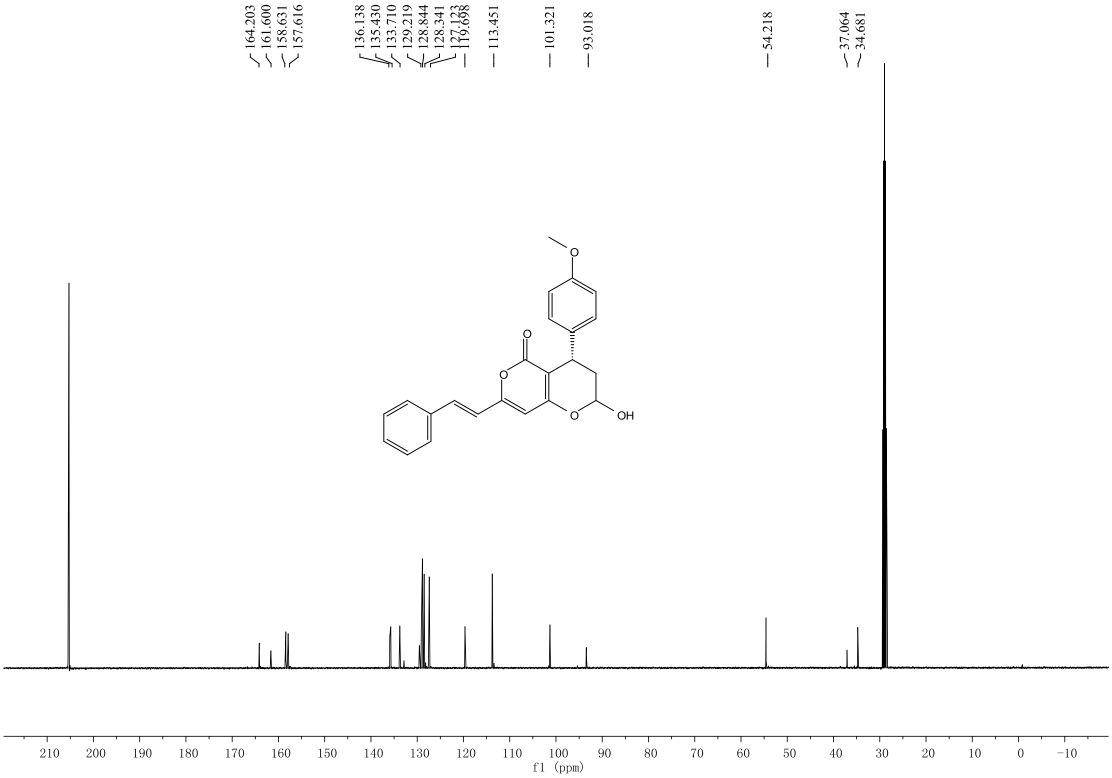

^{1}H NMR Spectrum for **5f** (acetone-d_6, 400 MHz)

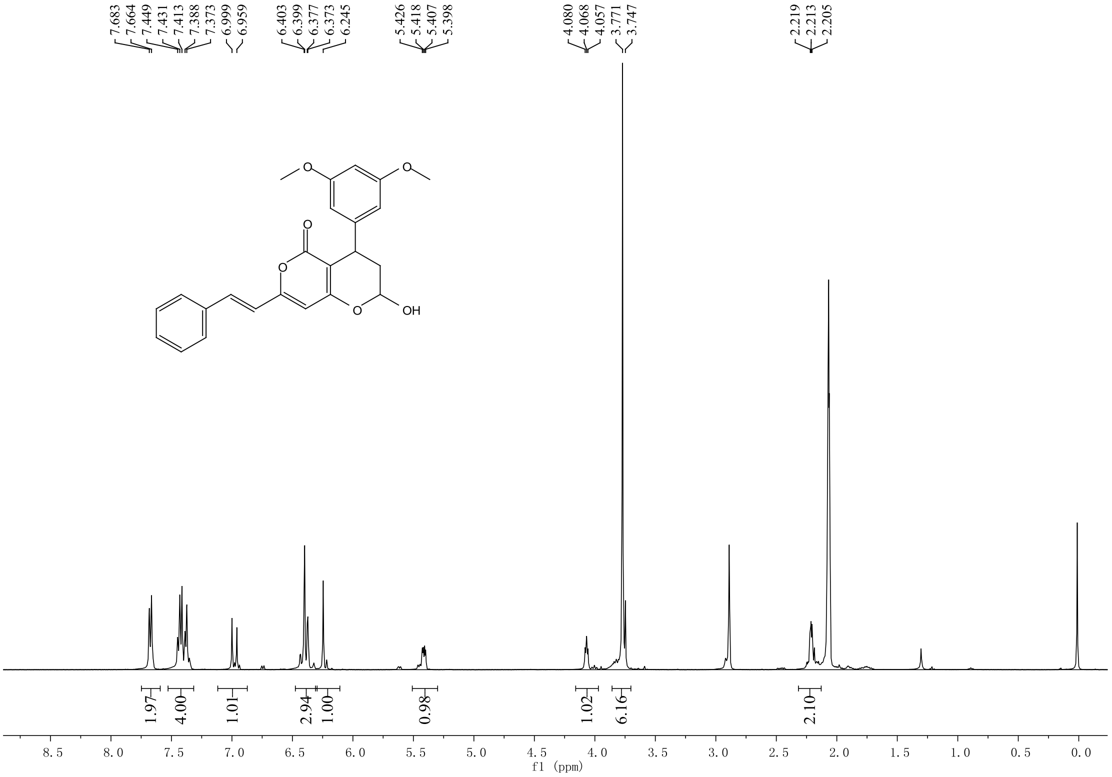

^{13}C NMR Spectrum for **5f** (acetone-d_6, 101 MHz)

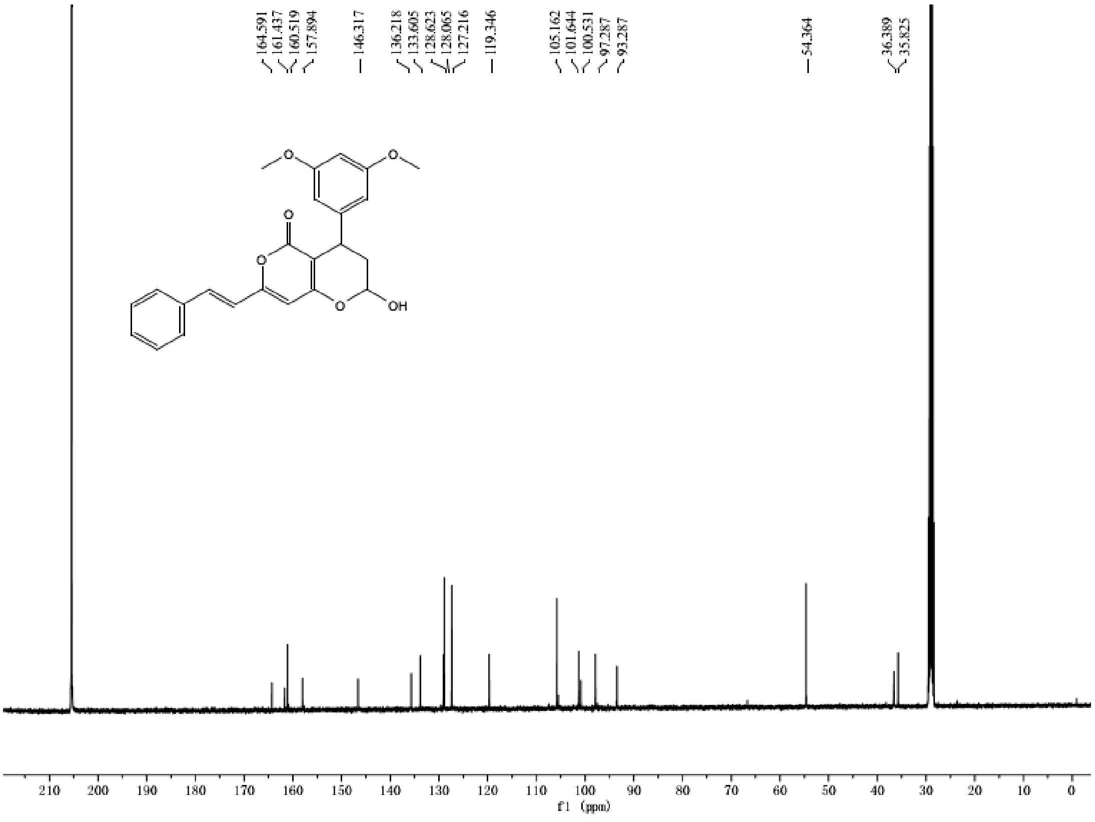

^{1}H NMR Spectrum for **5g** (acetone-d_6, 400 MHz)

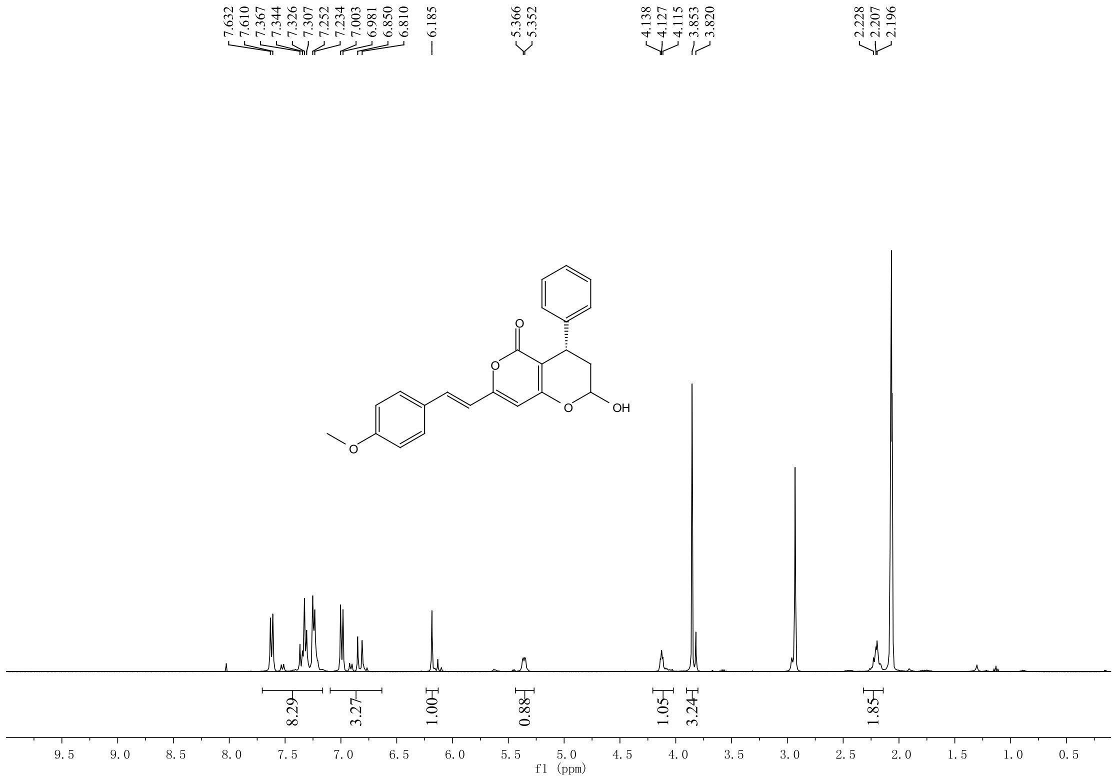

^{13}C NMR Spectrum for **5g** (acetone-d_6, 101 MHz)

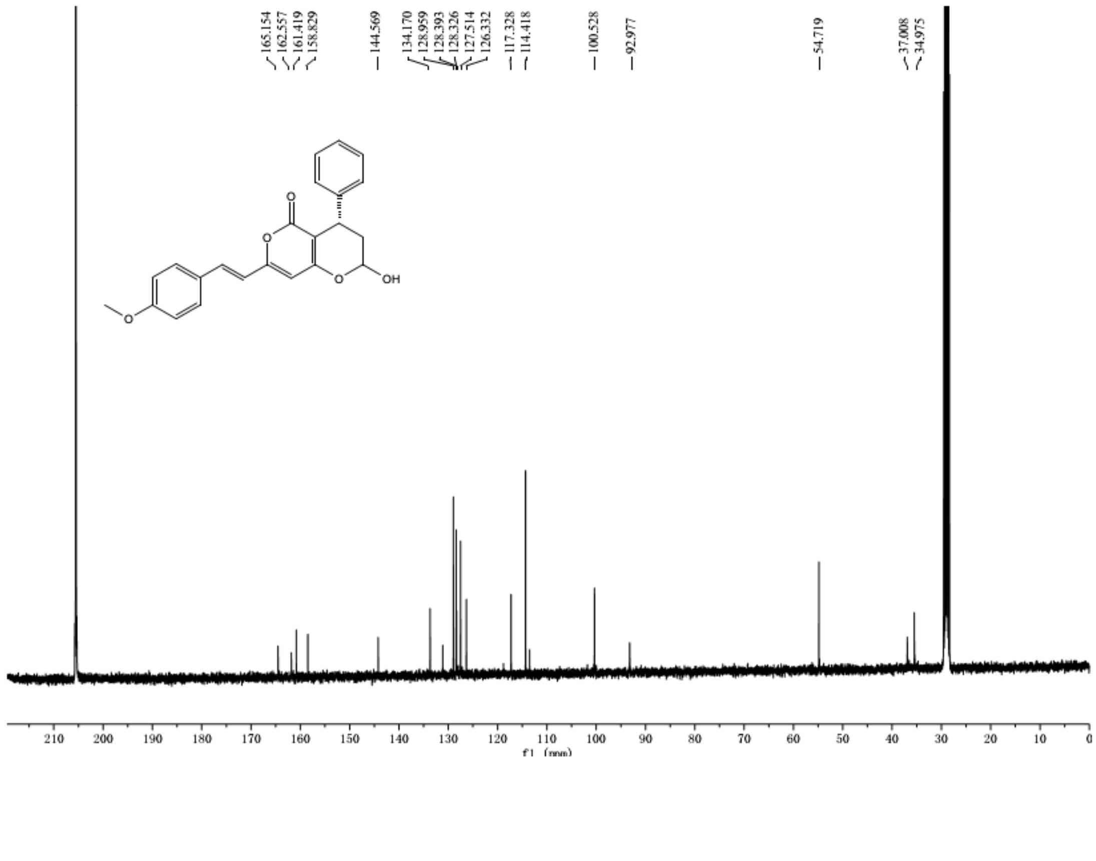

^{1}H NMR Spectrum for **5h** (acetone-d_6, 400 MHz)

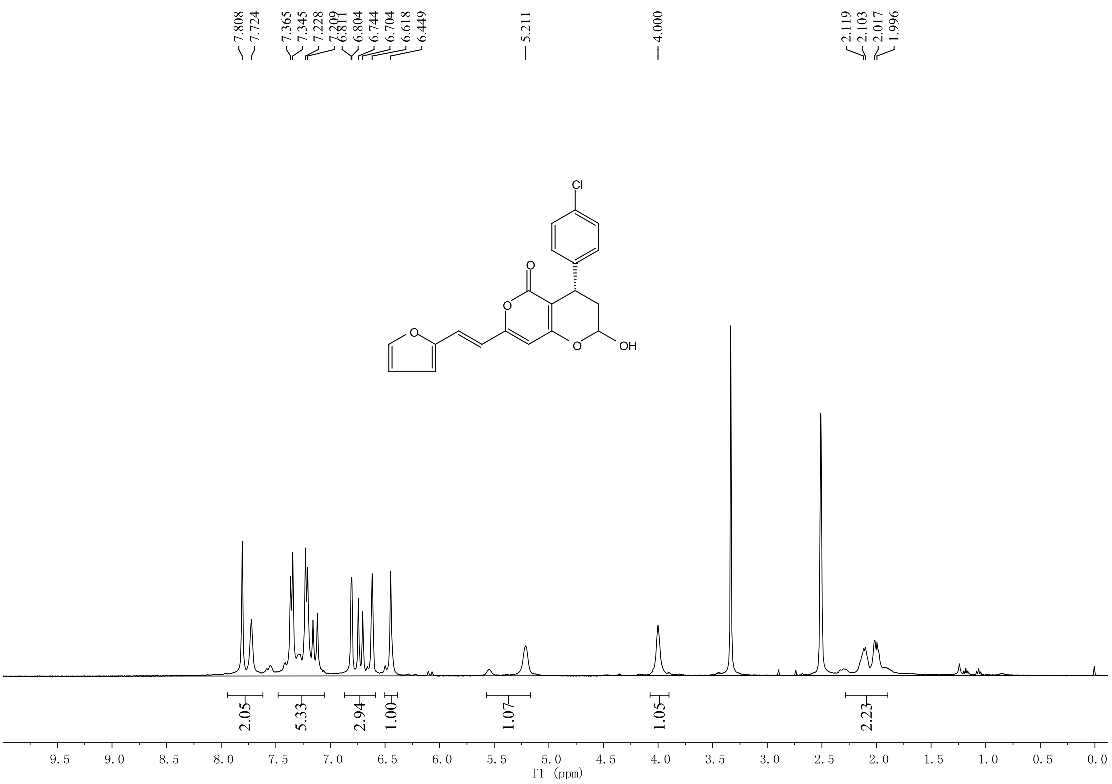

^{13}C NMR Spectrum for **5h** (acetone-d_6, 101 MHz)

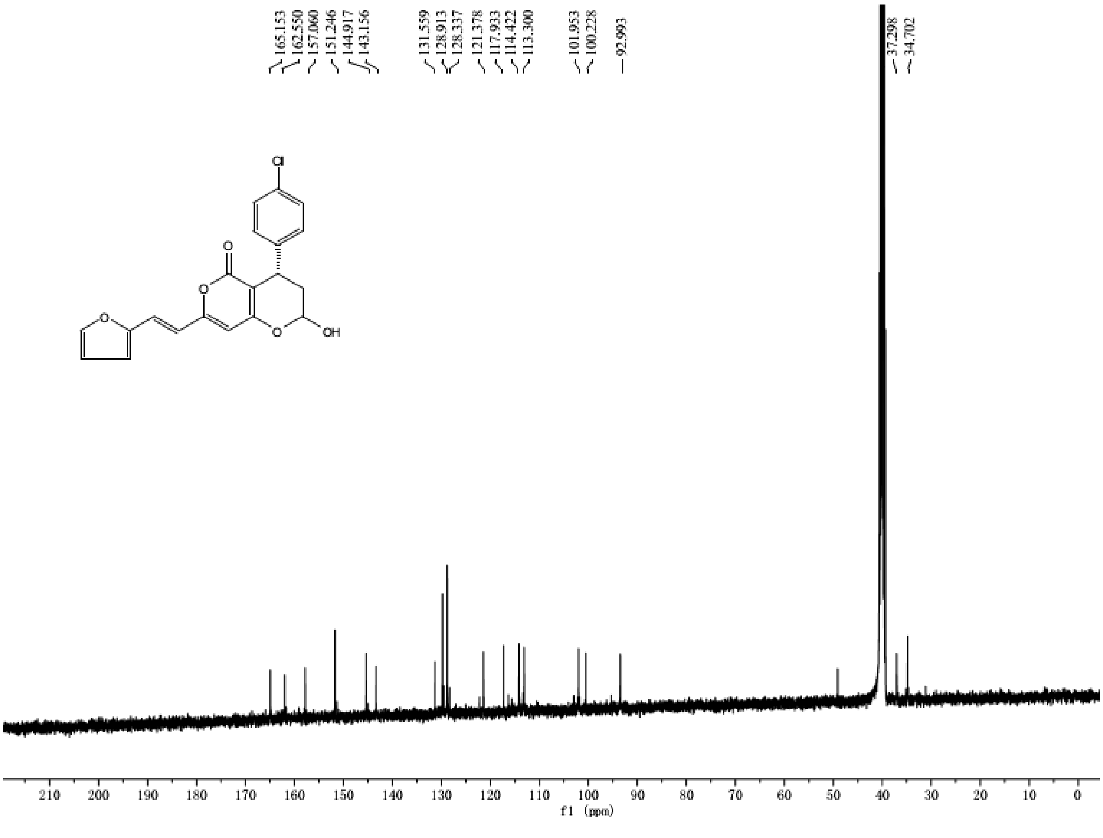

^{1}H NMR Spectrum for **5i** (acetone-d_6, 400 MHz)

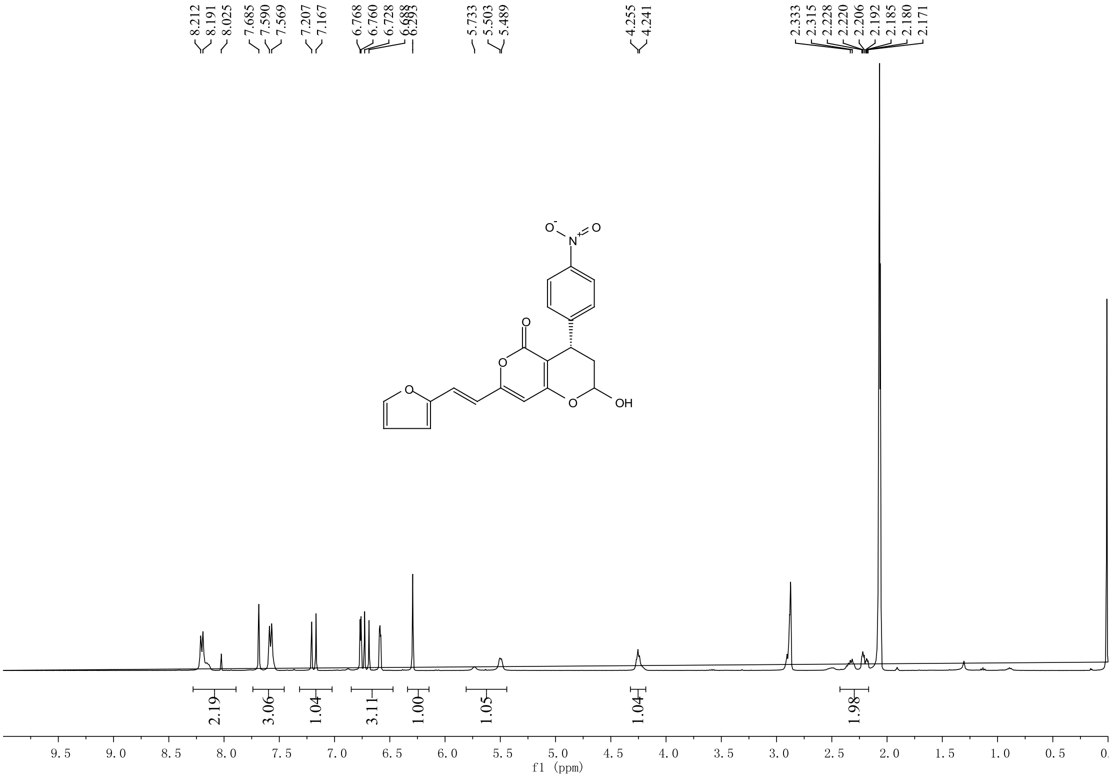

^{13}C NMR Spectrum for **5i** (acetone-d_6, 101 MHz)

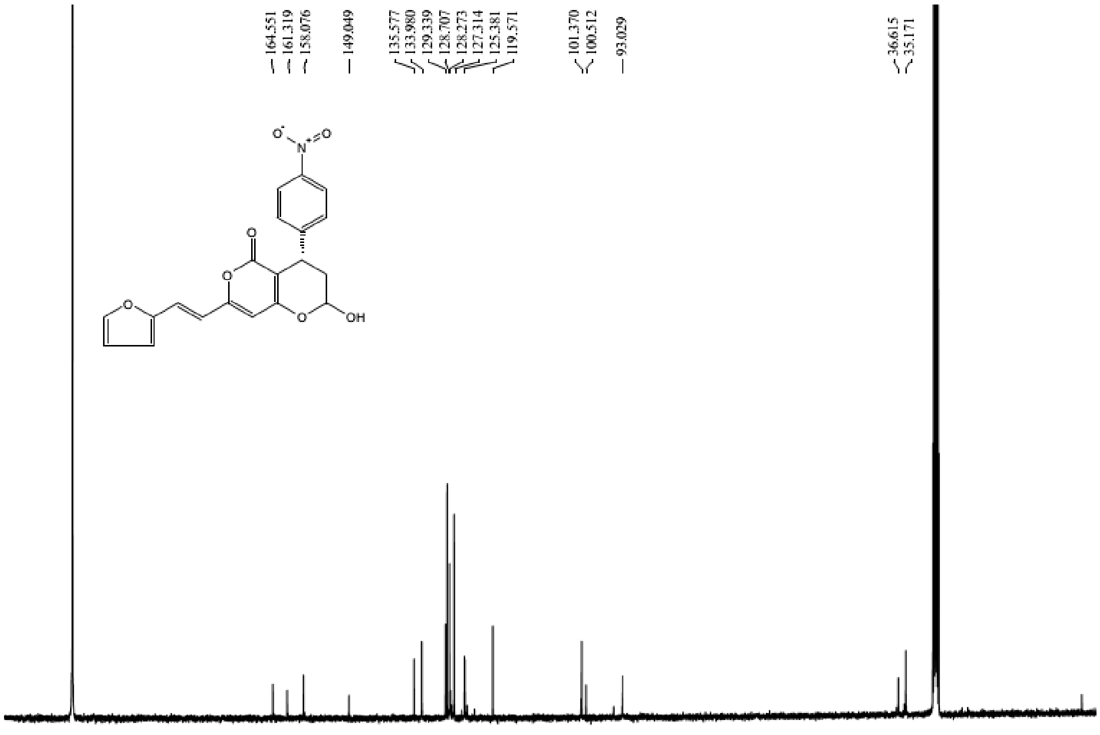

^{1}H NMR Spectrum for **5j** (acetone-d_6, 400 MHz)

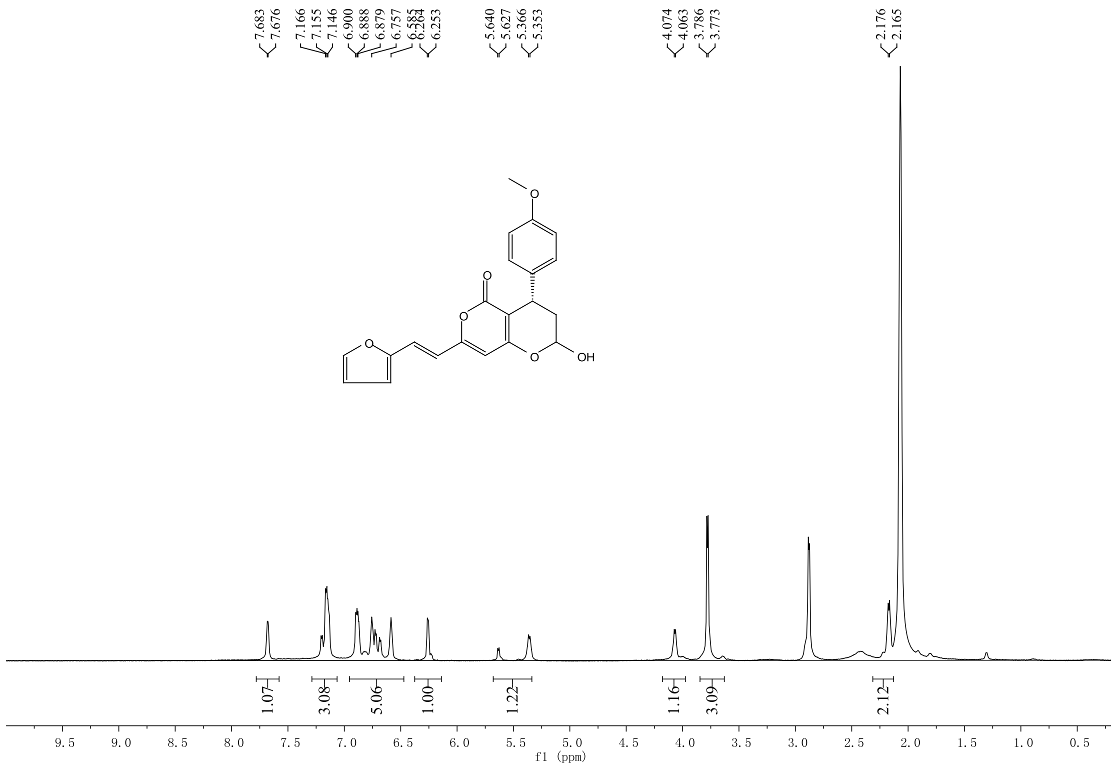

^{13}C NMR Spectrum for **5j** (acetone-d_6, 101 MHz)

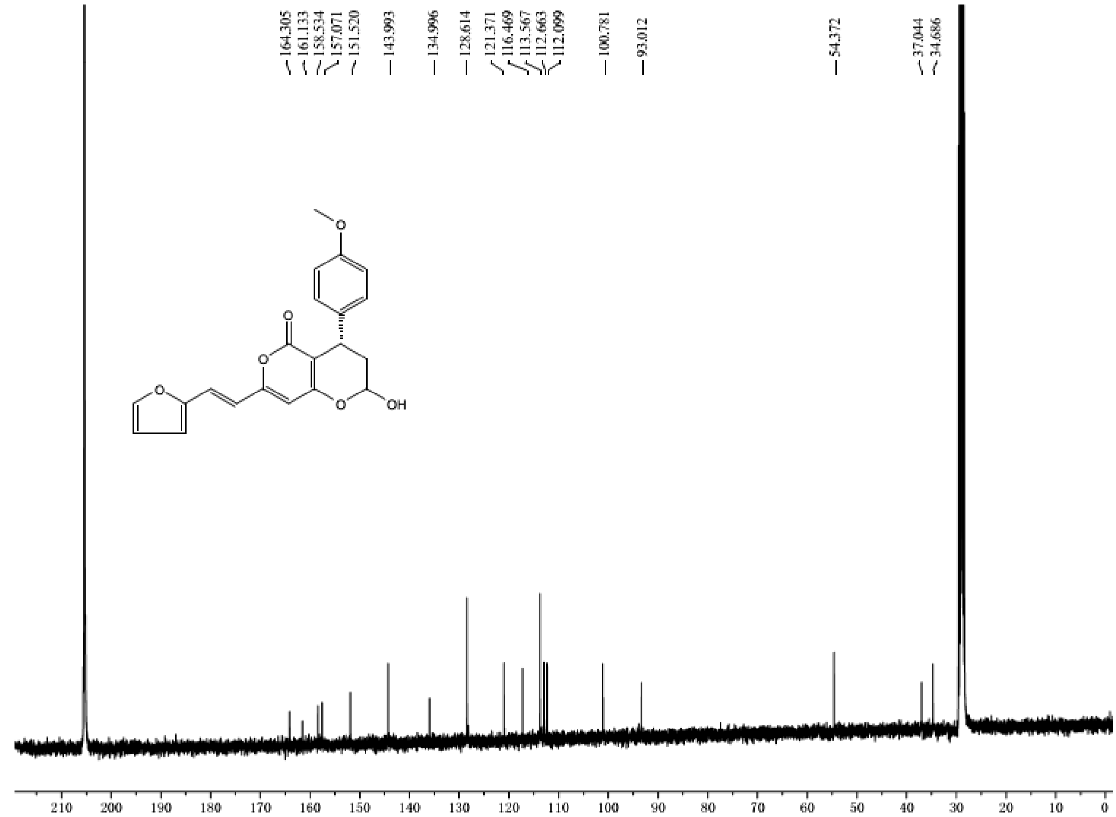

^{1}H NMR Spectrum for **5k** (acetone-d_6, 400 MHz)

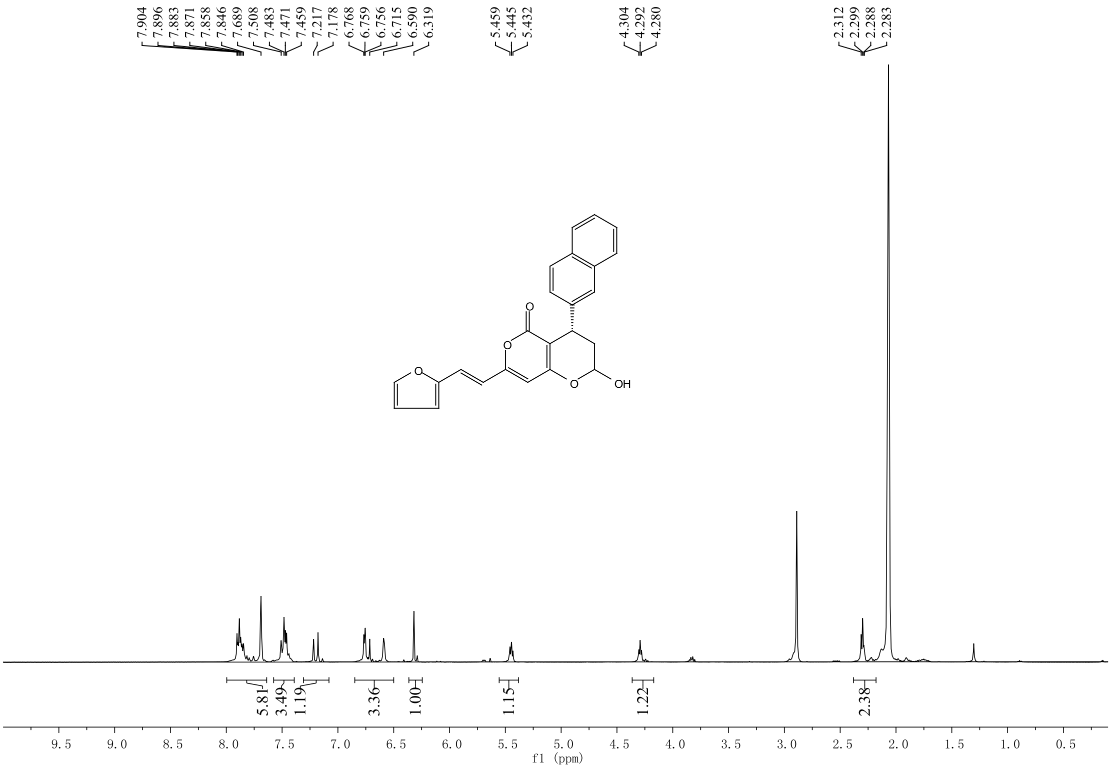

^{13}C NMR Spectrum for **5k** (acetone-d_6, 101 MHz)

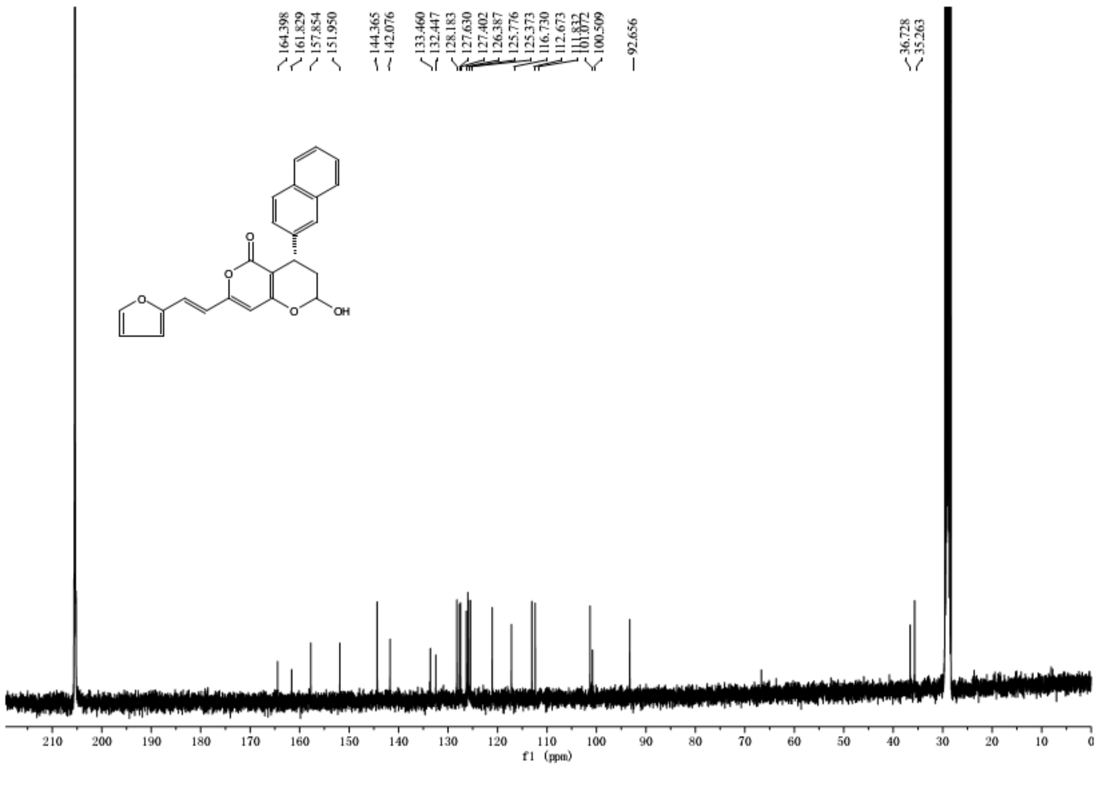

^{1}H NMR Spectrum for **S-5** ($CDCl_3$, 400 MHz)

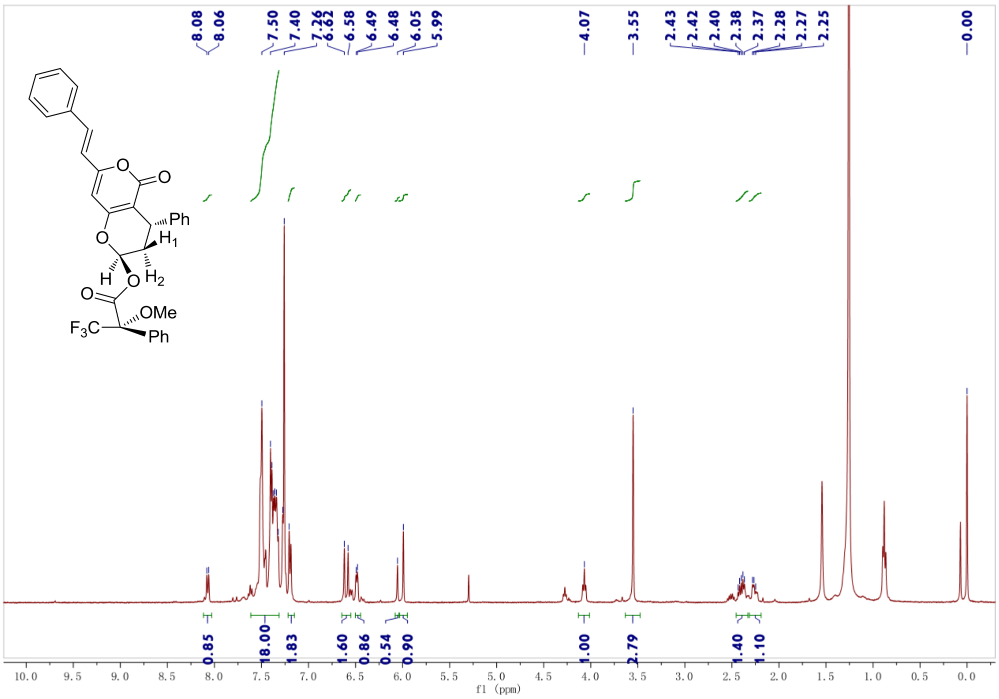

^{1}H NMR Spectrum for **S-6** ($CDCl_3$, 400 MHz)

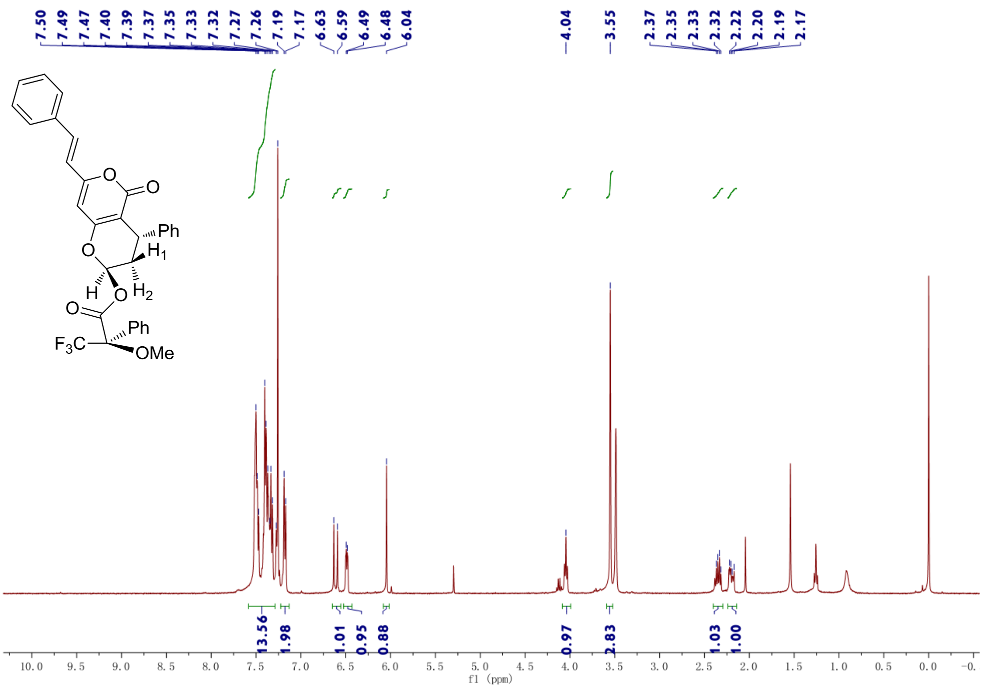

^{1}H NMR Spectrum for **9a** ($CDCl_3$, 400 MHz)

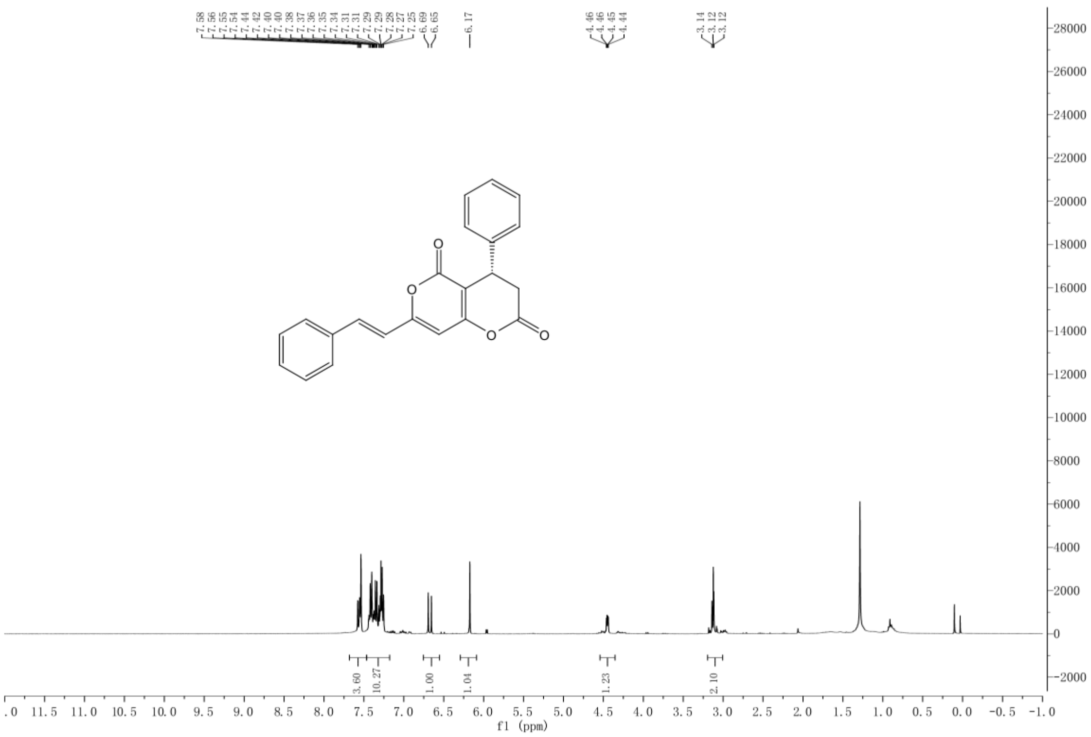

^{13}C NMR Spectrum for **9a** ($CDCl_3$, 101 MHz)

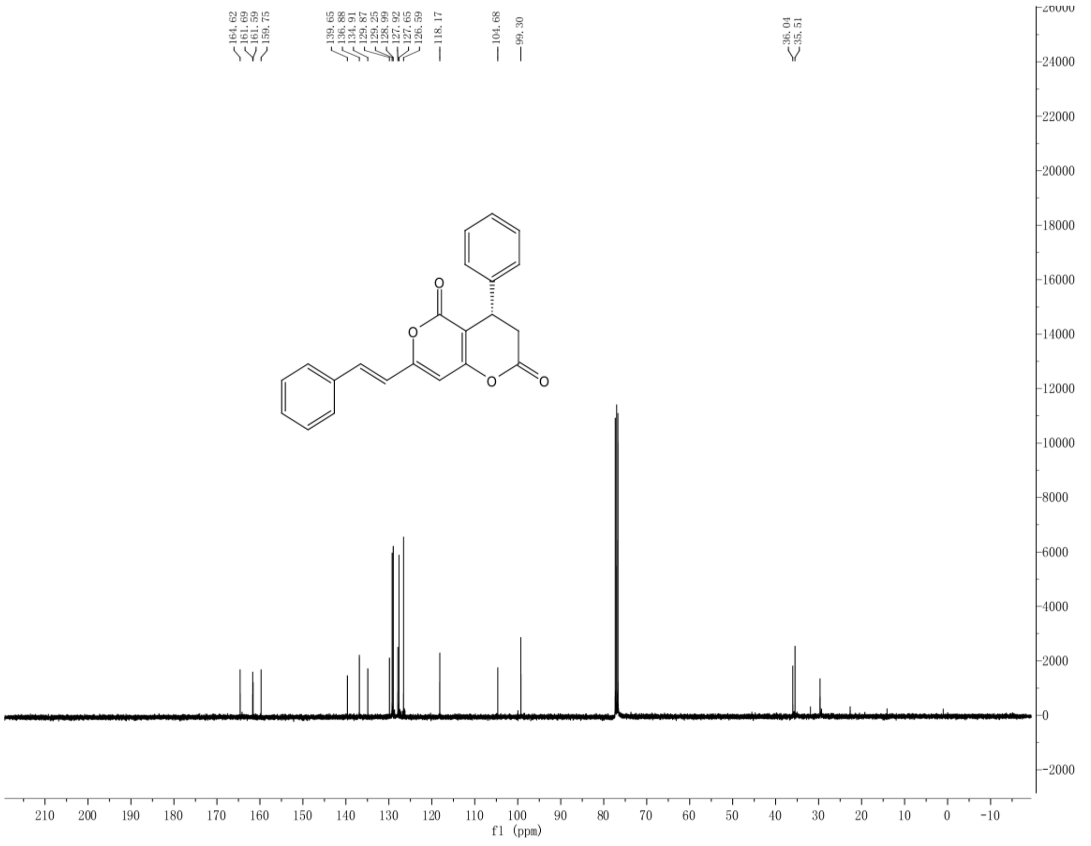

^{1}H NMR Spectrum for **9b** ($CDCl_3$, 400 MHz)

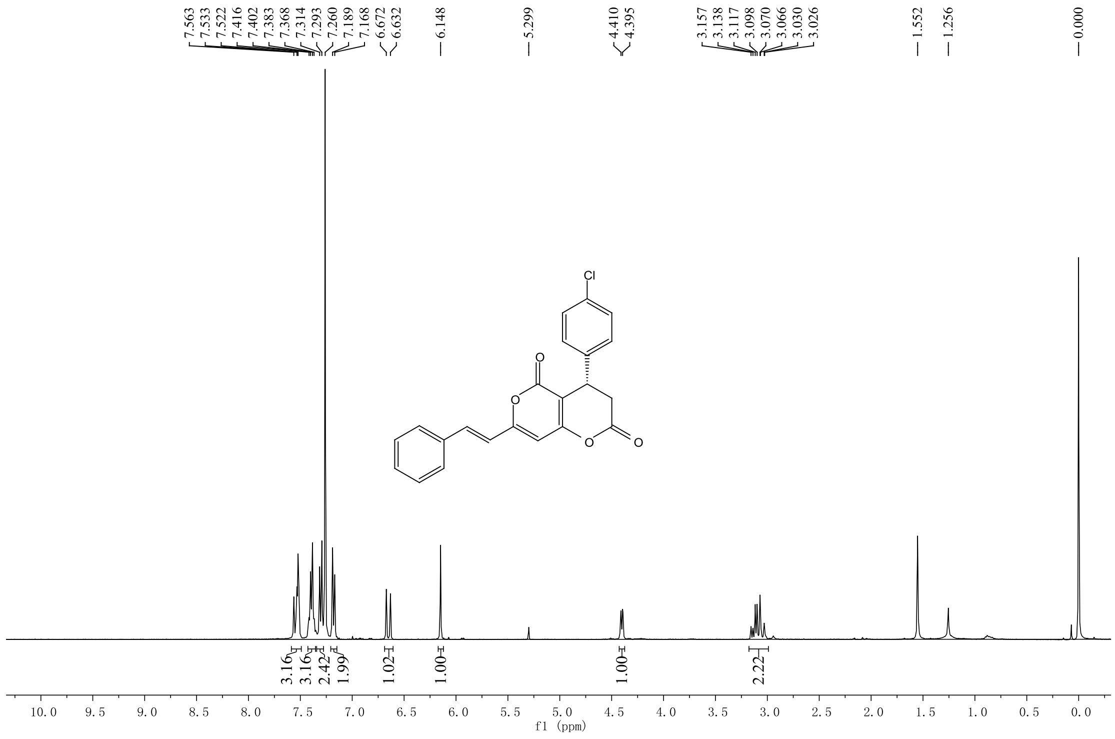

^{13}C NMR Spectrum for **9b** ($CDCl_3$, 101 MHz)

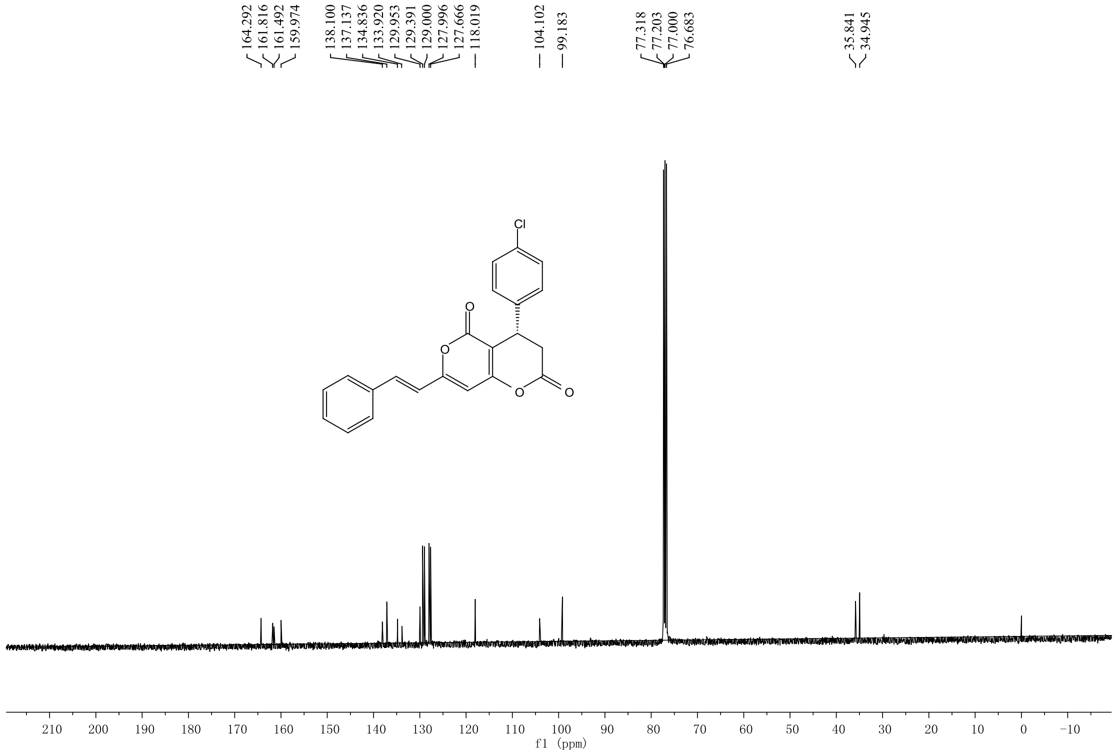

^{1}H NMR Spectrum for **9c** ($CDCl_3$, 400 MHz)

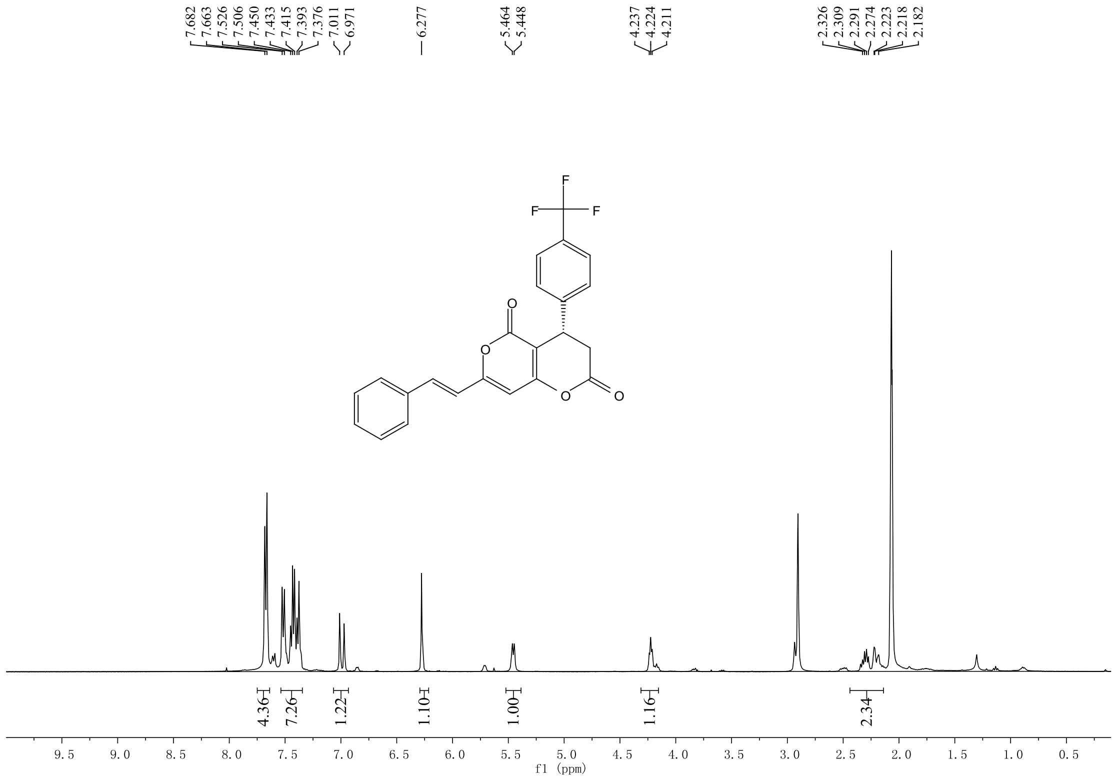

^{13}C NMR Spectrum for **9c** ($CDCl_3$, 101 MHz)

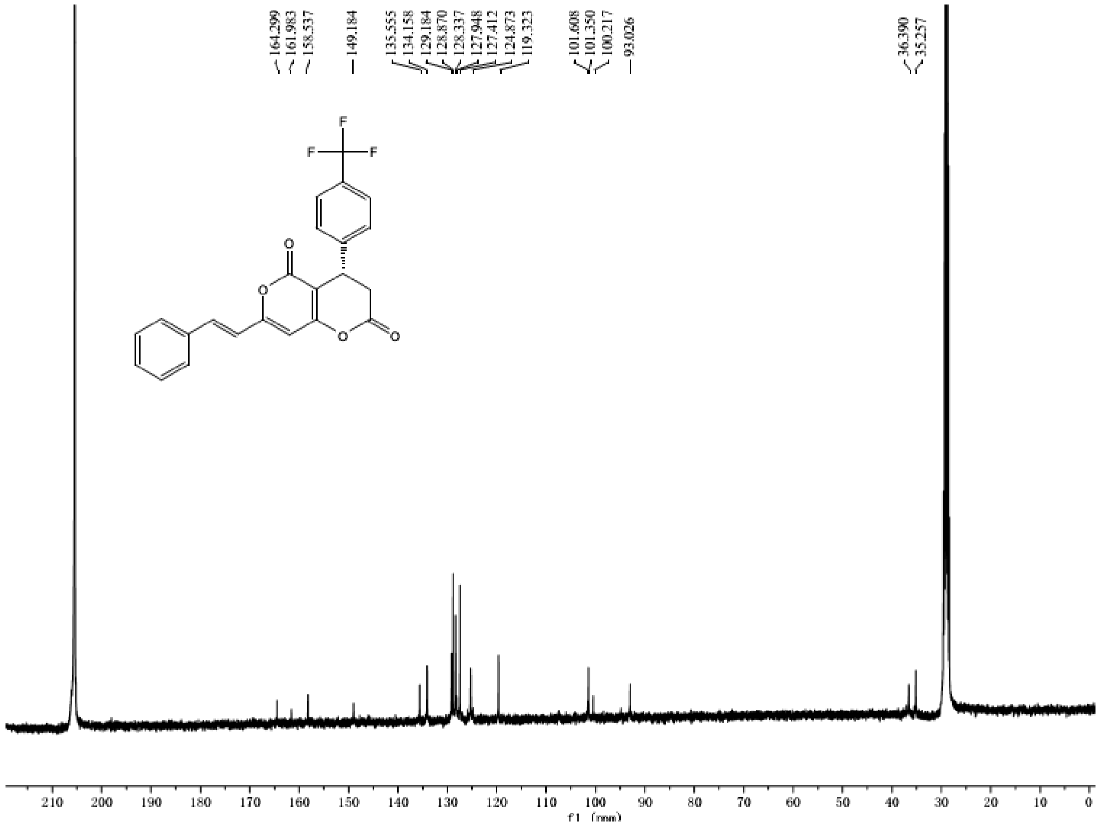

^{1}H NMR Spectrum for **9d** ($CDCl_3$, 400 MHz)

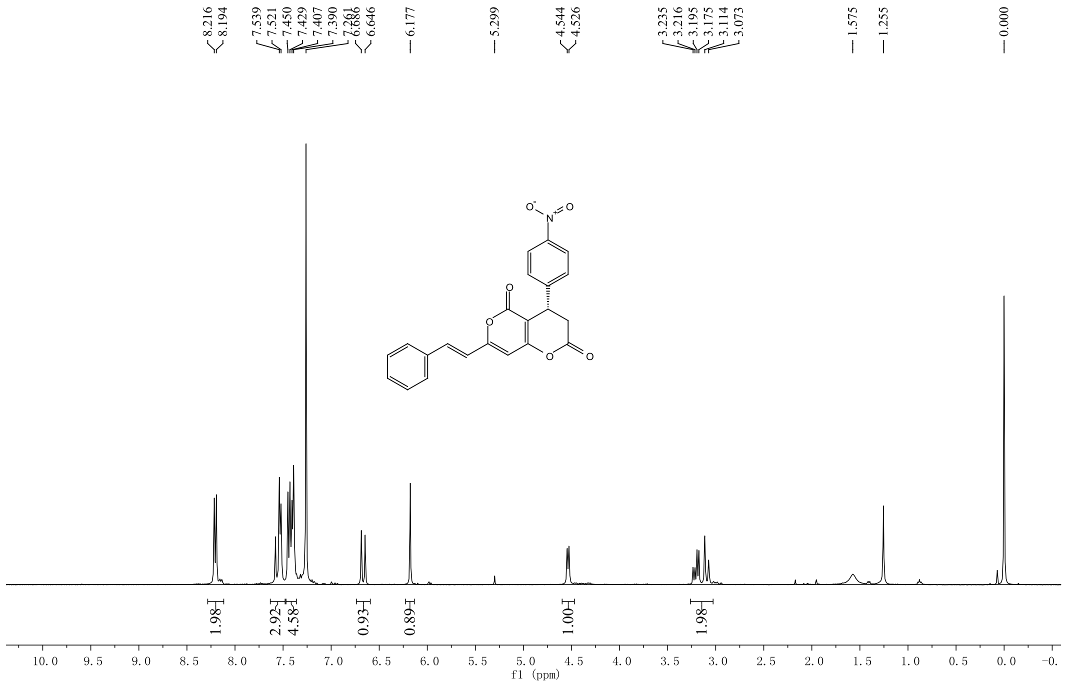

^{13}C NMR Spectrum for **9d** ($CDCl_3$, 101 MHz)

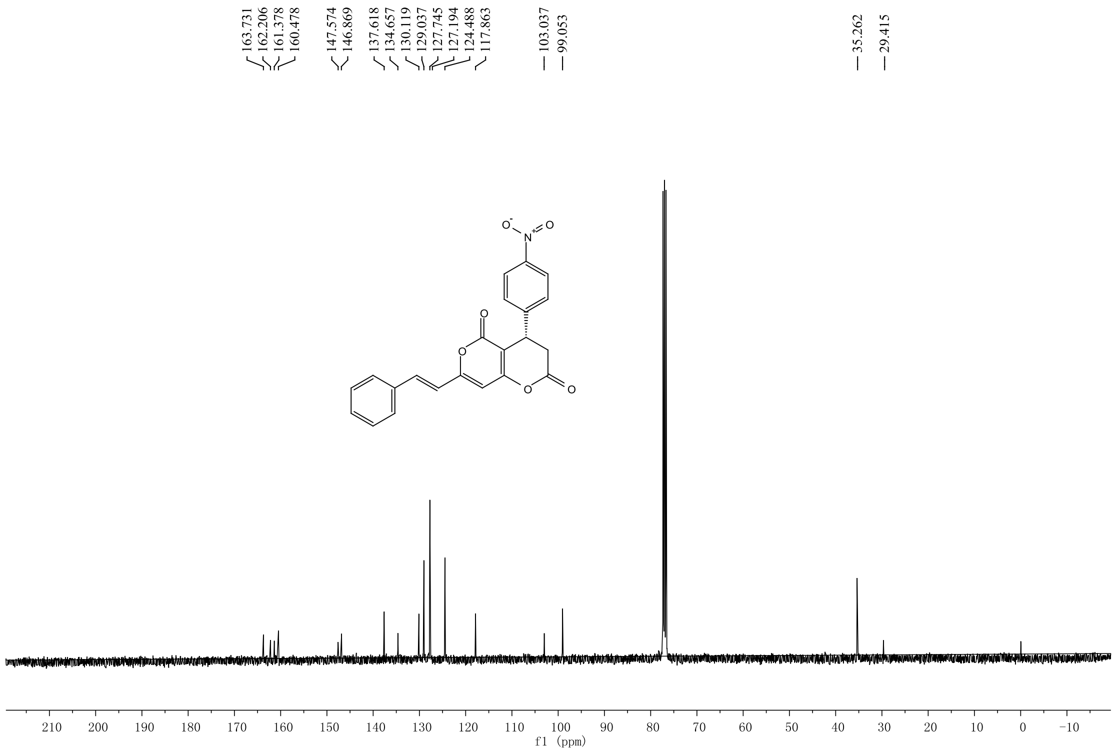

^{1}H NMR Spectrum for **9e** ($CDCl_3$, 400 MHz)

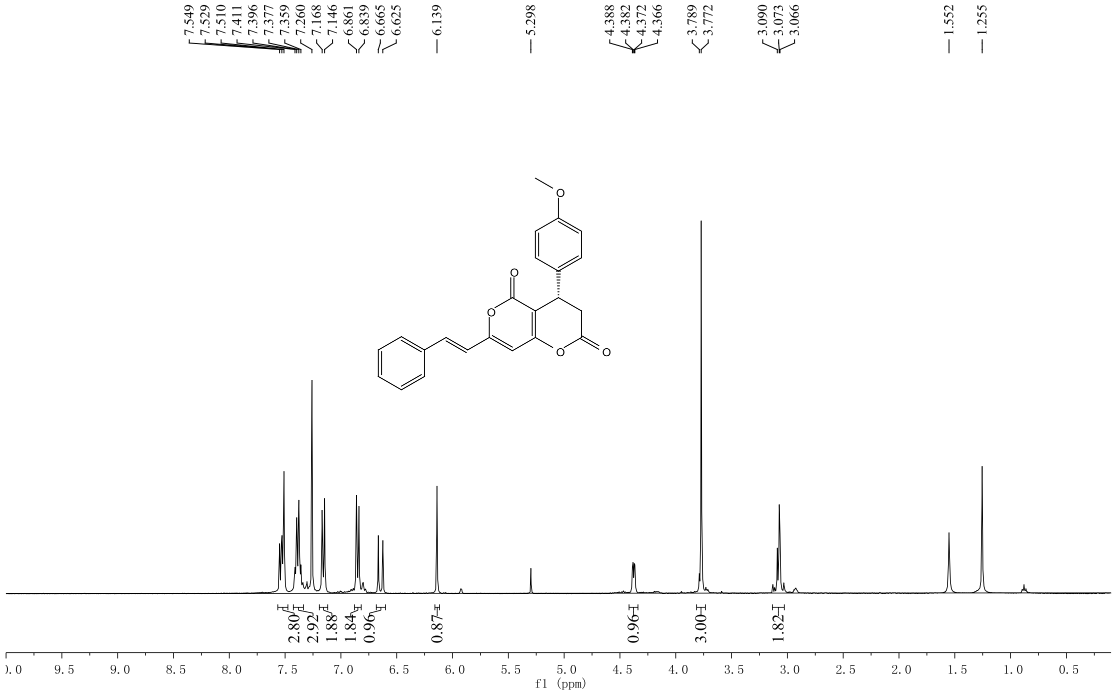

^{13}C NMR Spectrum for 9**e** ($CDCl_3$, 101 MHz)

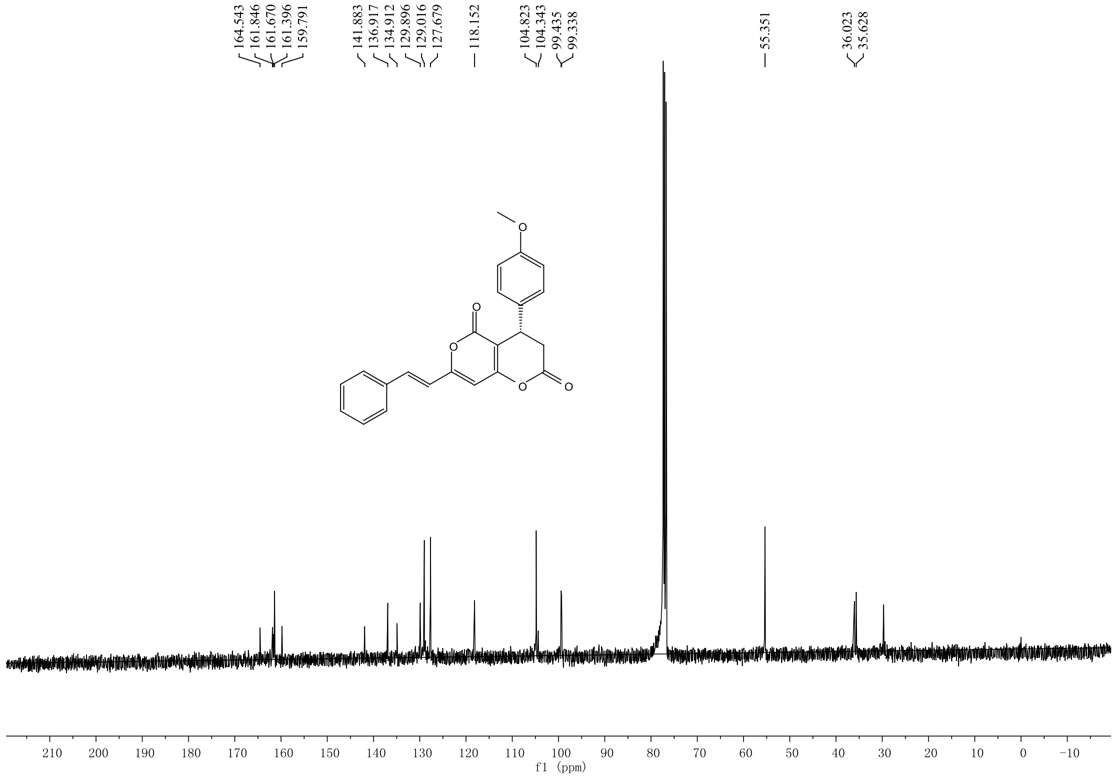

^{1}H NMR Spectrum for **9f** ($CDCl_3$, 400 MHz)

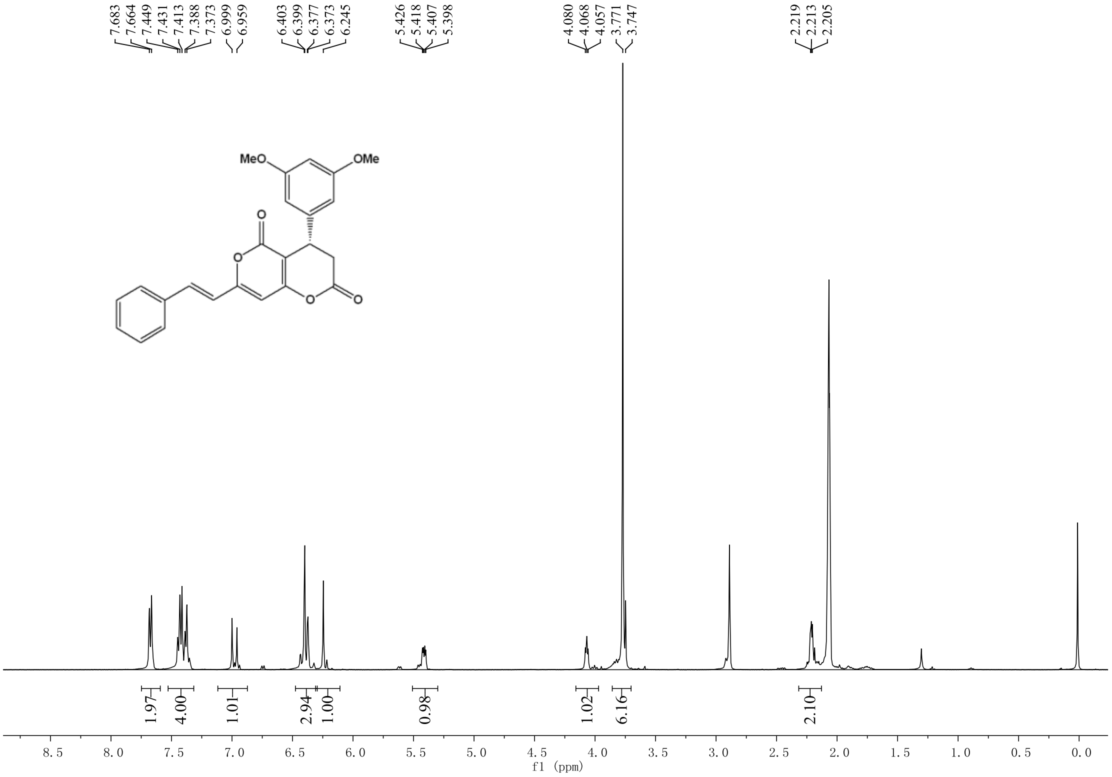

^{13}C NMR Spectrum for **9f** ($CDCl_3$, 101 MHz)

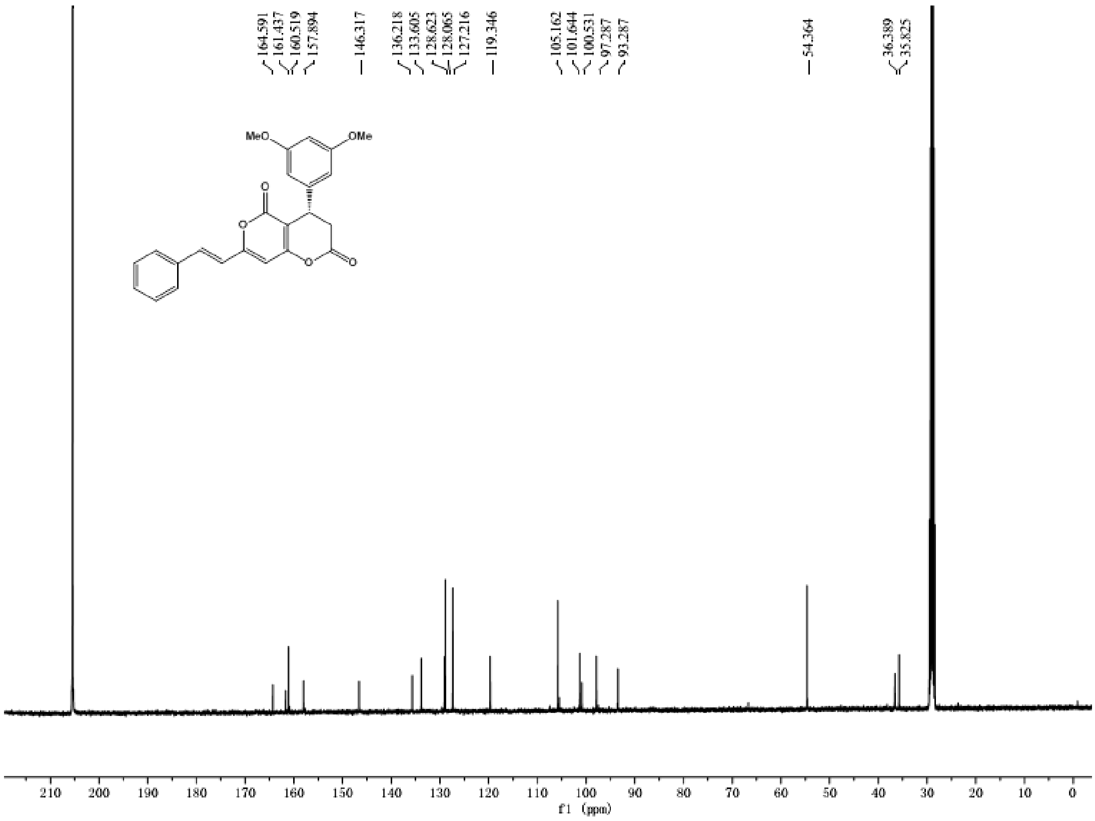

^{1}H NMR Spectrum for **9g** ($CDCl_3$, 400 MHz)

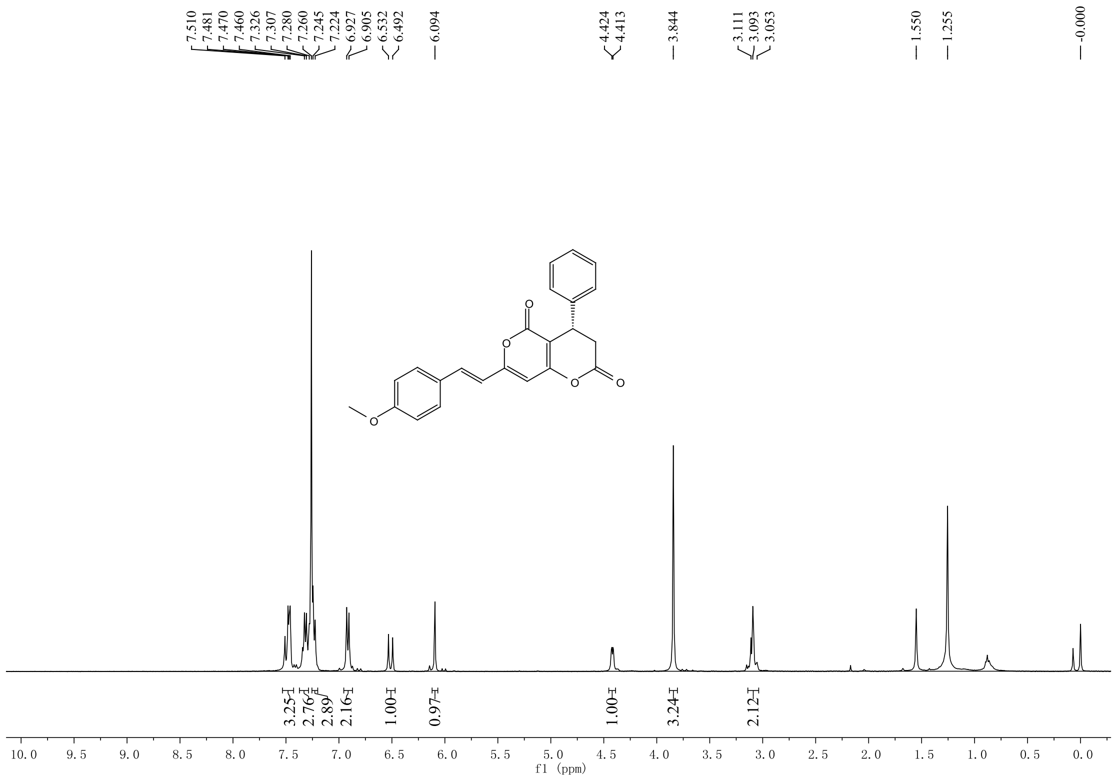

^{13}C NMR Spectrum for **9g** ($CDCl_3$, 101 MHz)

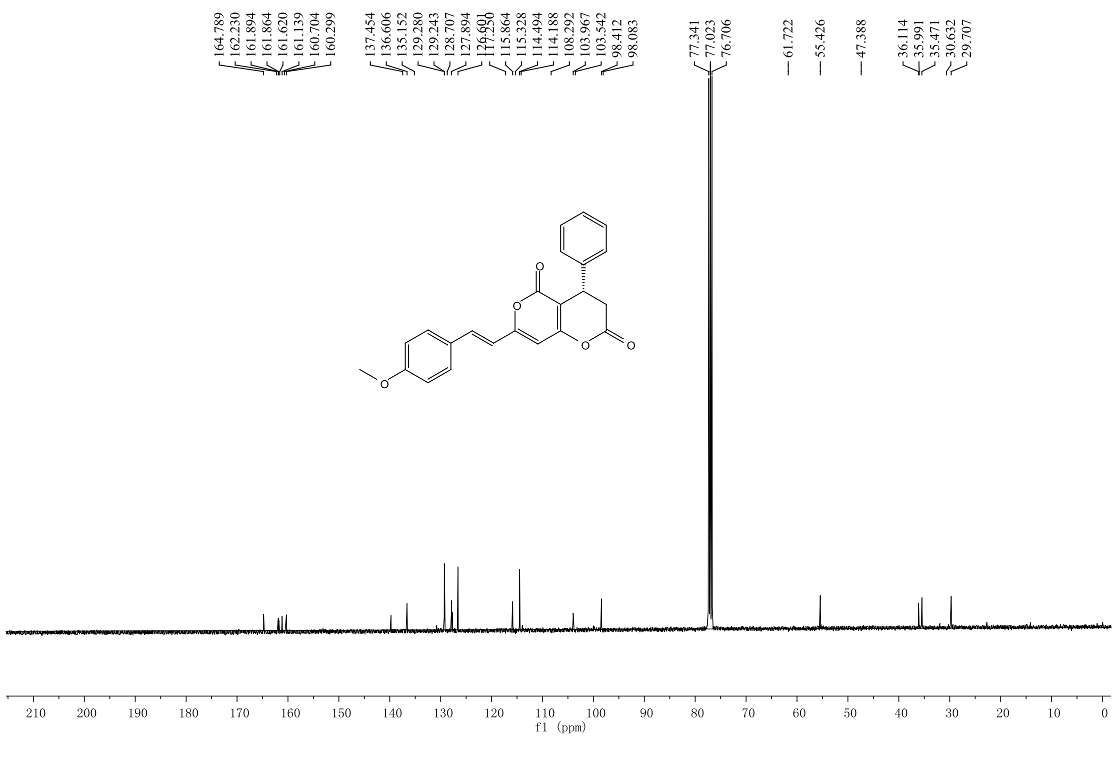

^{1}H NMR Spectrum for **9h** ($CDCl_3$, 400 MHz)

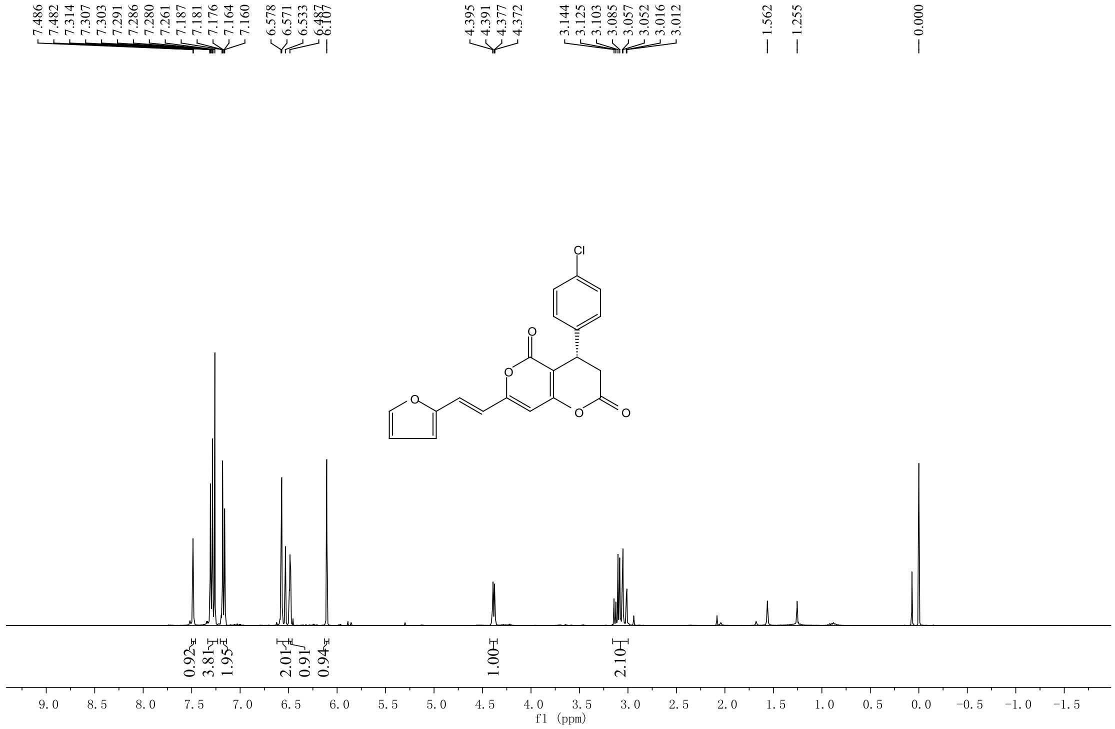

^{13}C NMR Spectrum for **9h** ($CDCl_3$, 101 MHz)

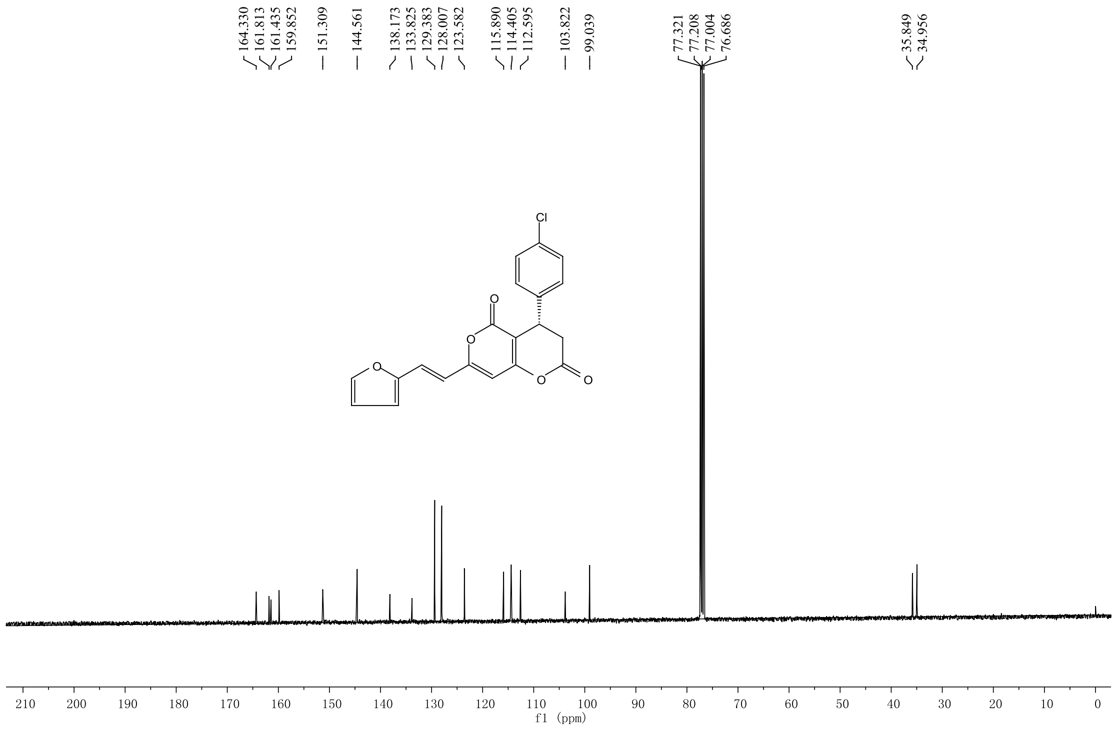

^{1}H NMR Spectrum for **9i** ($CDCl_3$, 400 MHz)

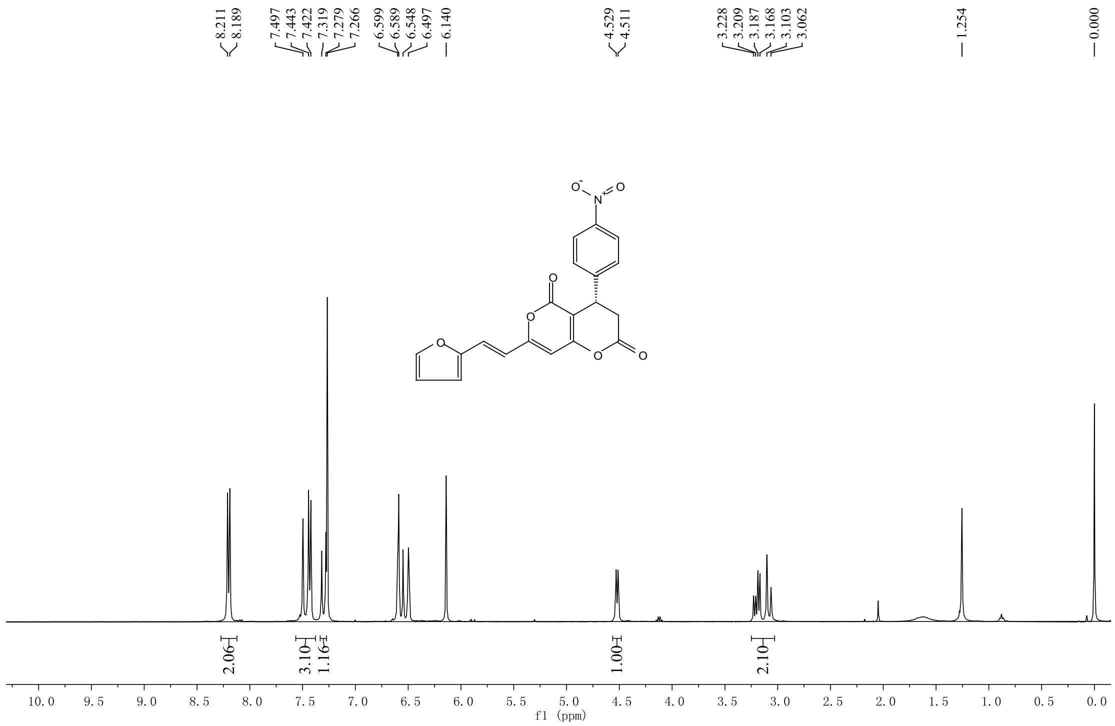

^{13}C NMR Spectrum for **9i** ($CDCl_3$, 101 MHz)

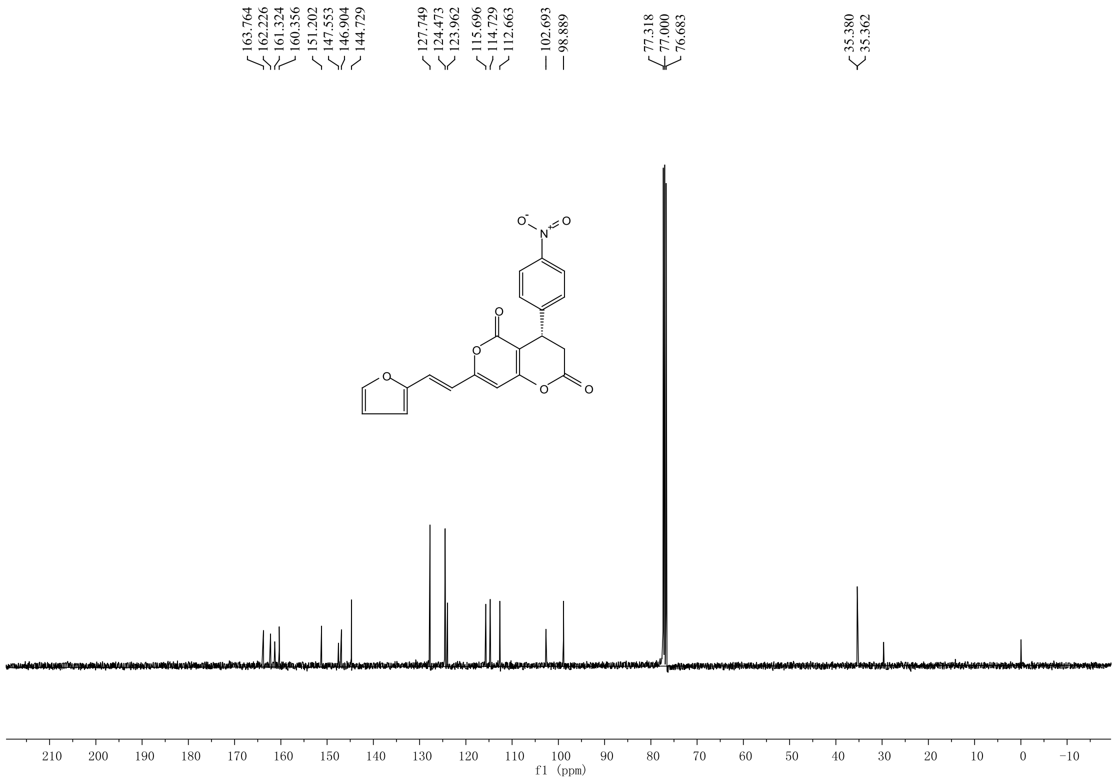

^{1}H NMR Spectrum for **9j** ($CDCl_3$, 400 MHz)

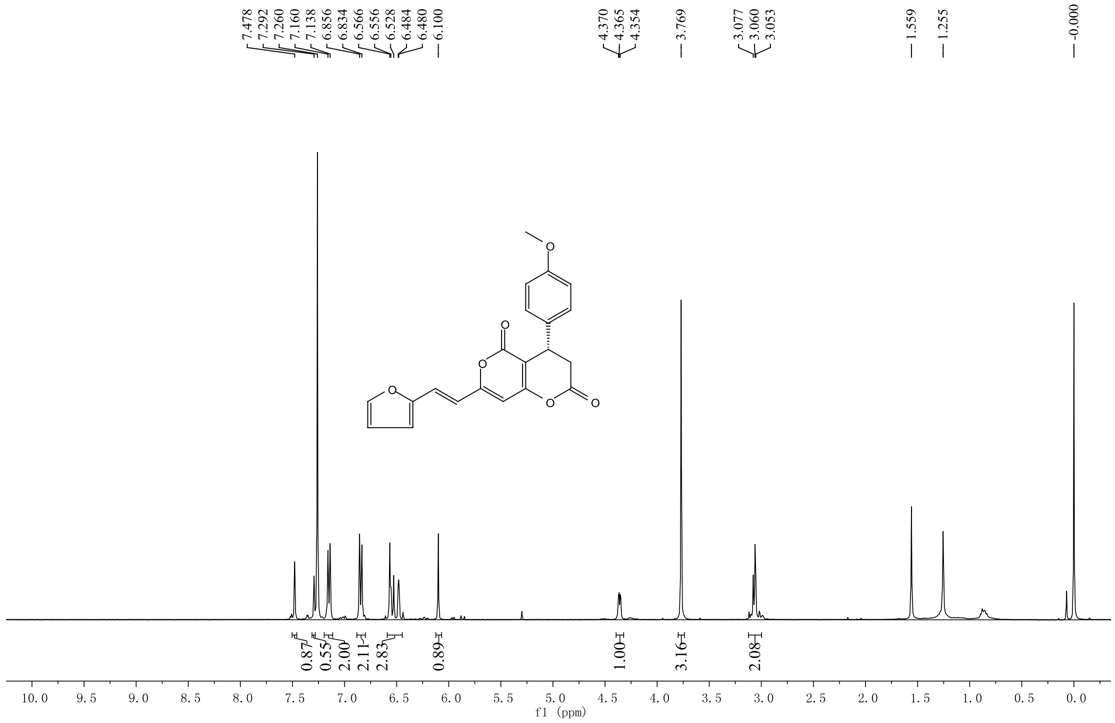

^{13}C NMR Spectrum for **9j** ($CDCl_3$, 101 MHz)

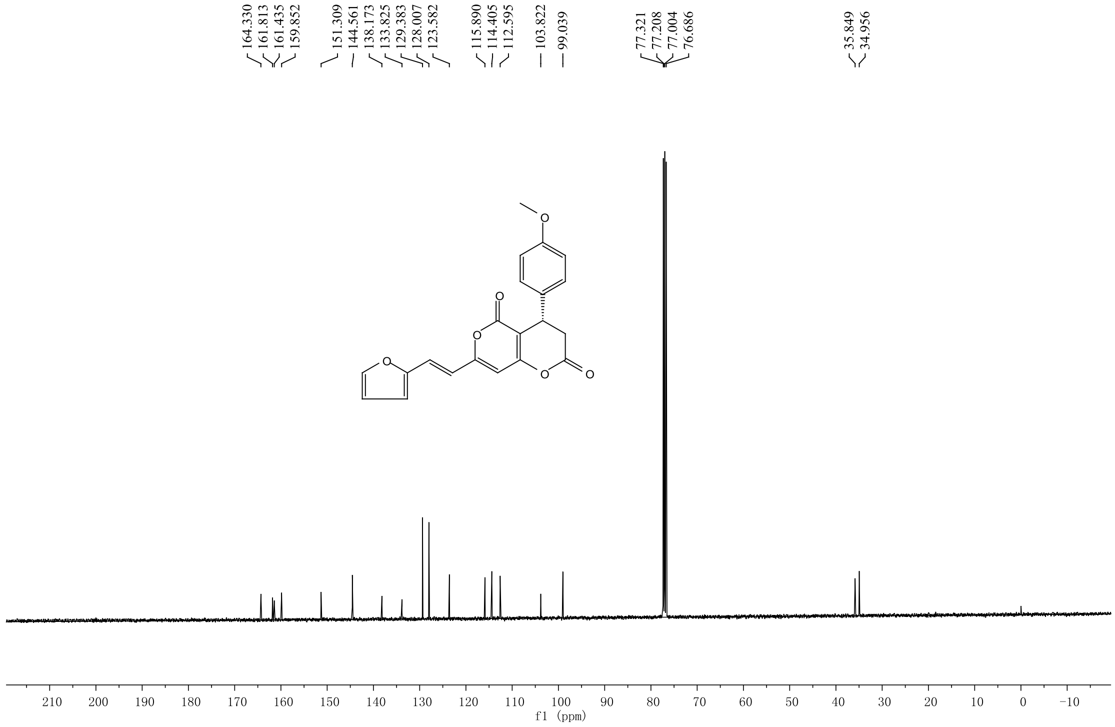

^{1}H NMR Spectrum for **9k** ($CDCl_3$, 400 MHz)

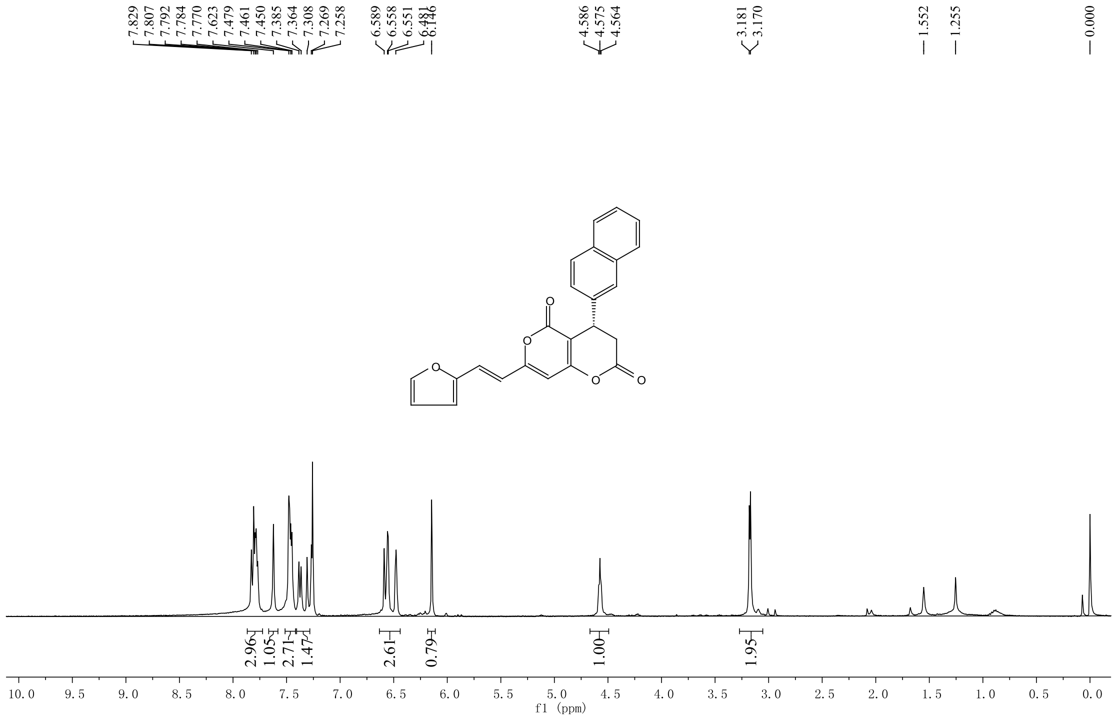

^{13}C NMR Spectrum for **9k** ($CDCl_3$, 101 MHz)

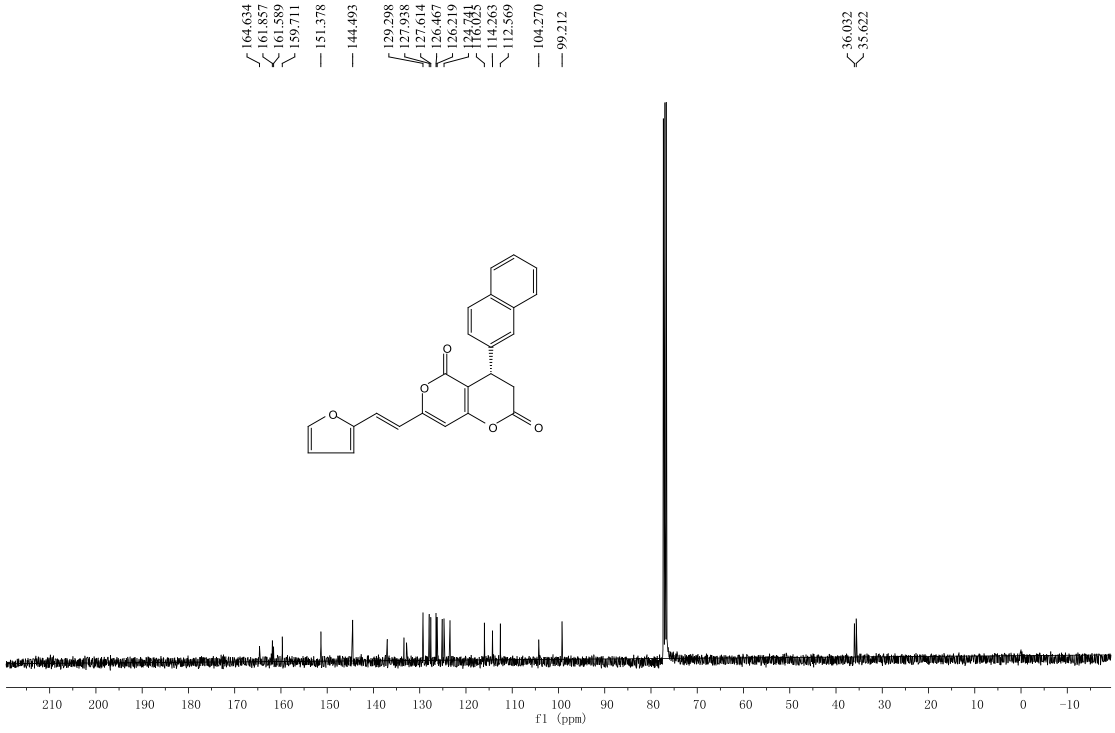

^{1}H NMR Spectrum for katsumadain A ($CDCl_3$, 400 MHz)

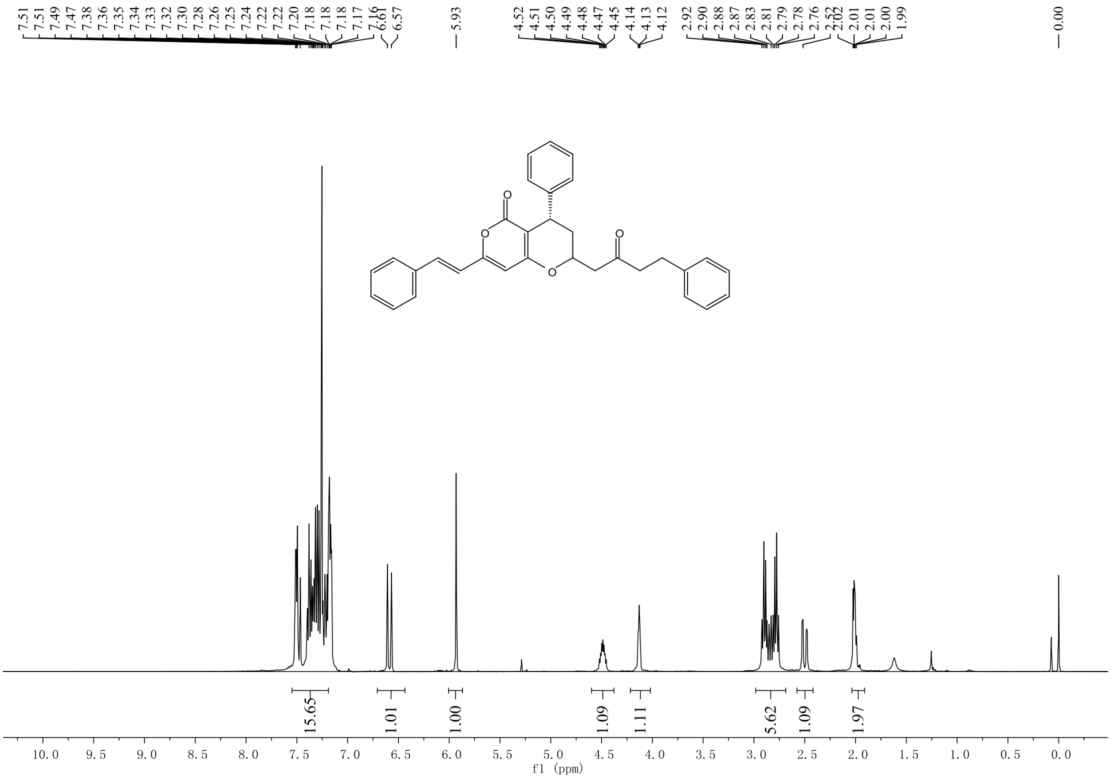

^{13}C NMR Spectrum for katsumadain A ($CDCl_3$, 101 MHz)

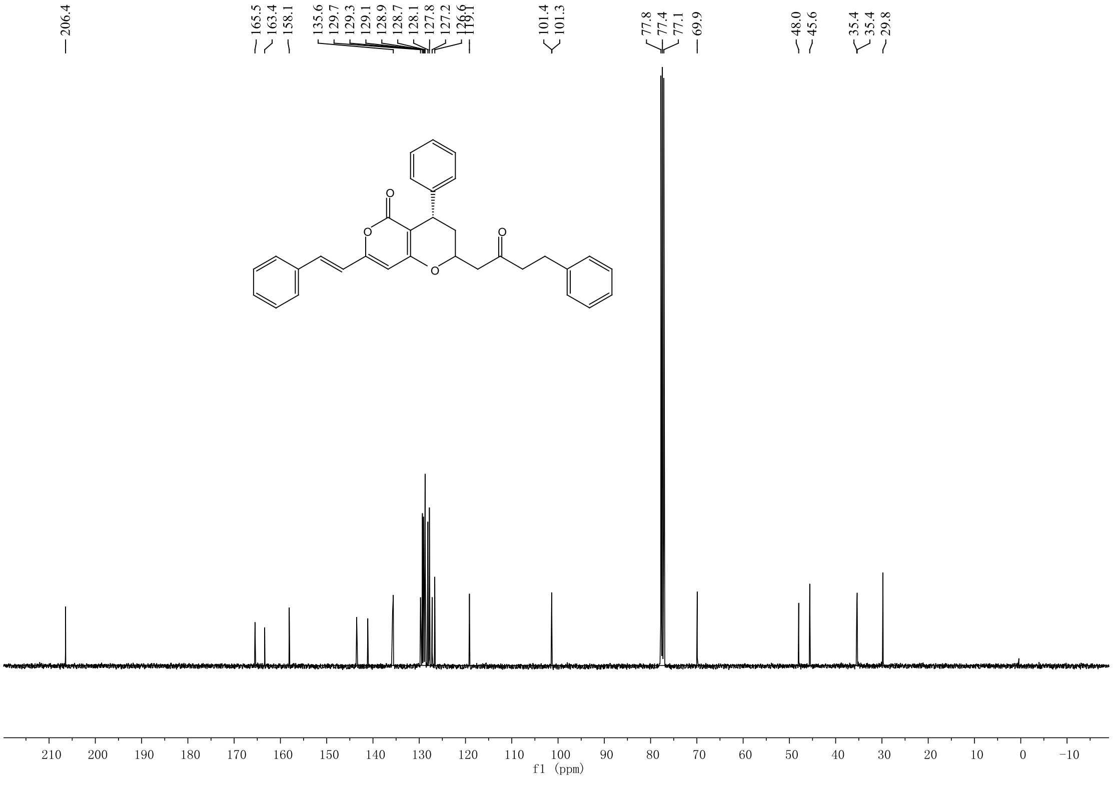

Comparison of NMR spectrum of synthetic Katsumadain A ($CDCl_3$, 400 MHz) with that of natural katsumadain A ($CDCl_3$, 400 MHz)

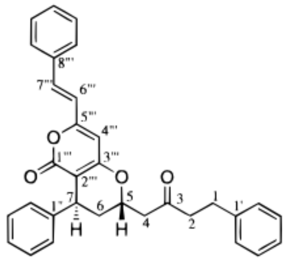

1

position	^{1}H NMR (δ in ppm, *J* in Hz)		^{13}C NMR (δ in ppm)	
	Natural	Synthetic	Natural	Synthetic
1	2.89 t (7.0)	2.90 t (7.0)	29.8	29.8
2	2.78 t (7.0)	2.78 t (7.0)	45.6	45.6
3			206.5	206.4
4	2.49 dd (16.0, 4.0)	2.50 dd (16.0, 4.0)	48.0	48.0
	2.83 dd (16.0, 8.0)	2.83 dd (16.0, 8.0)		
5	4.49 dddd (8.0, 8.0, 4.0, 4.0)	4.49 m	70.0	69.9
6	2.00 m	2.00 m	35.5	35.4
7	4.13 t (4)	4.13 t (4)	35.4	35.4
1'			141.1	141.1
1"			143.5	143.5
1"'			163.4	163.4
2"'			101.4	101.4
3"'			165.5	165.5
4"'	5.93 s	5.93 s	101.3	101.3
5"'			158.1	158.1
6"	6.58 d (16)	6.58 d (16)	119.2	119.1
7"'	7.47 d (16)	7.47 d (16)	135.6	135.6
9"'			135.8	135.8
three phenyl	7.50–7.16 m	7.51–7.16 m	126.2–129.3	126.6–129.7

Part 3: HPLC chart of 9b–k

The ee was determined by HPLC analysis using a CHIRALPAK-AD (Column NO. AD00CE-AJ123) column (CH_3CN/H_2O = 40/60, 1.0 mL/min, 254 nm).

HPLC for **9b**

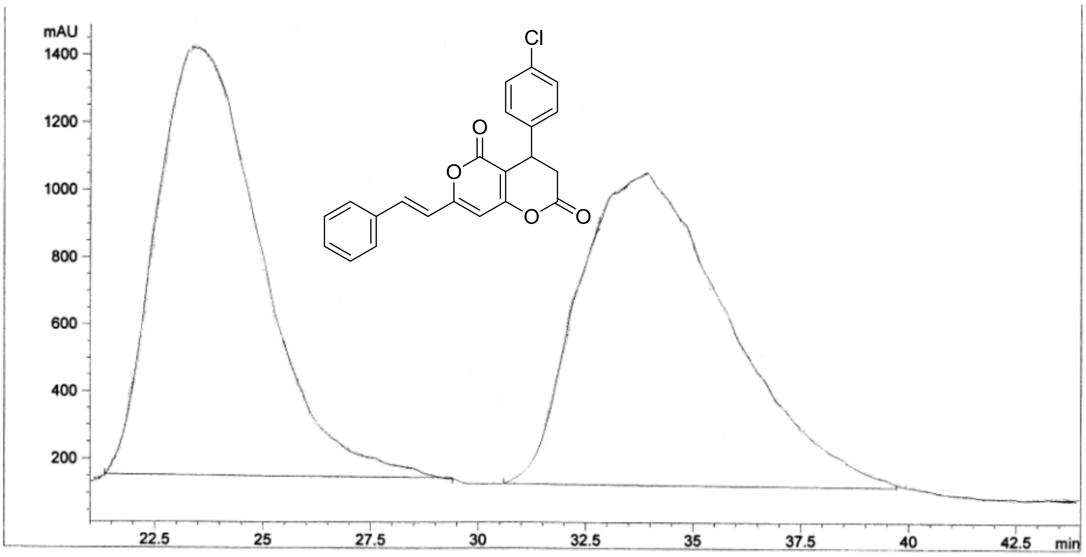

Peak #	RetTime [min]	Type	Width [min]	Area [mAU*s]	Area %	Name
1	23.377	MM	2.8046	2.14136e5	48.8356	?
2	33.905	MM	4.0390	2.24347e5	51.1644	?

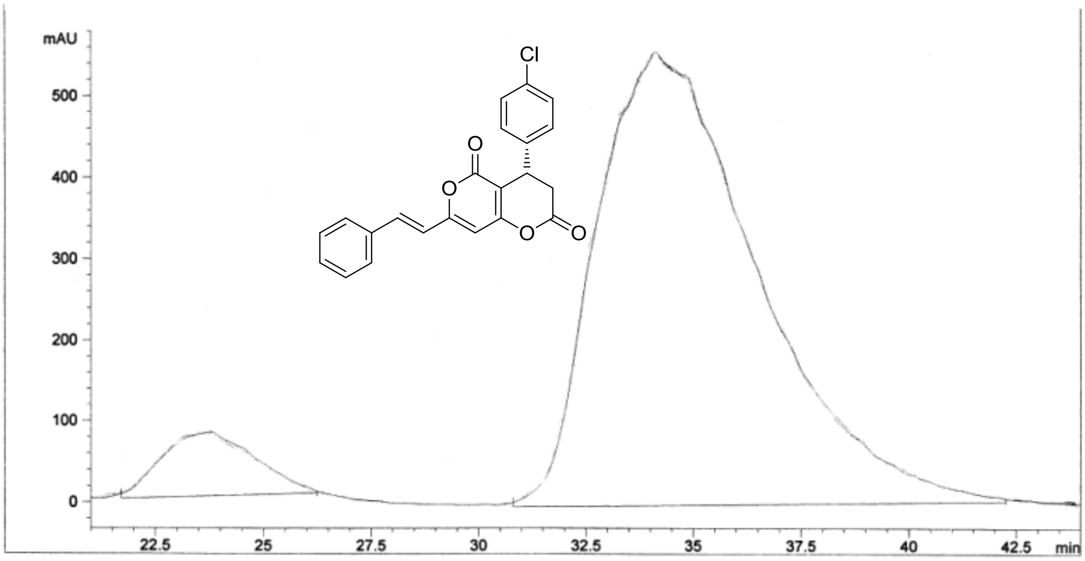

Peak #	RetTime [min]	Type	Width [min]	Area [mAU*s]	Area %	Name
1	23.784	MM	2.5019	1.17508e4	7.4845	?
2	34.110	MM	4.3404	1.45251e5	92.5155	?

HPLC for **9c**

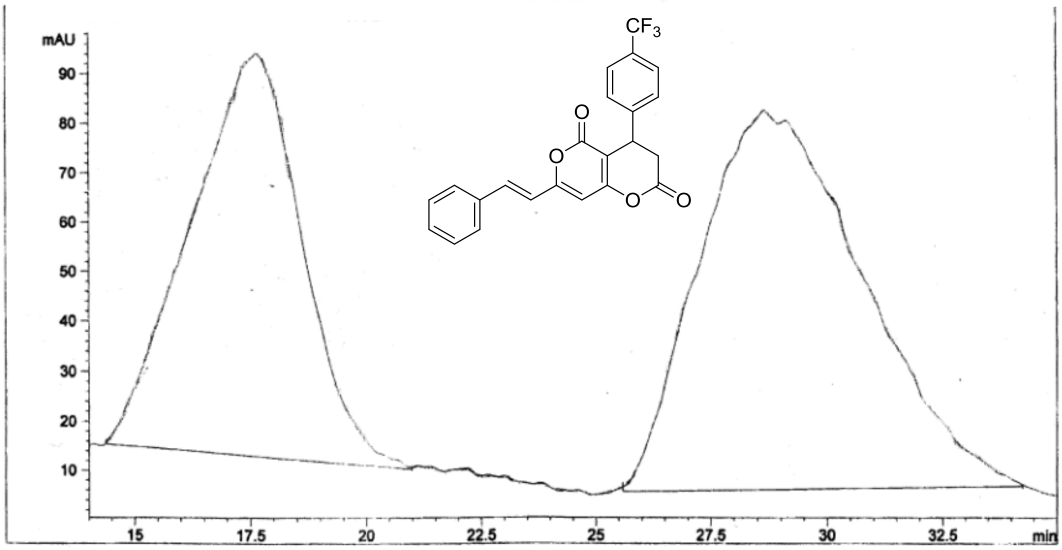

Peak #	RetTime [min]	Type	Width [min]	Area [mAU*s]	Area %	Name
1	17.658	MM	2.4262	1.18310e4	49.4897	?
2	28.668	MM	3.3367	1.20750e4	50.5103	?

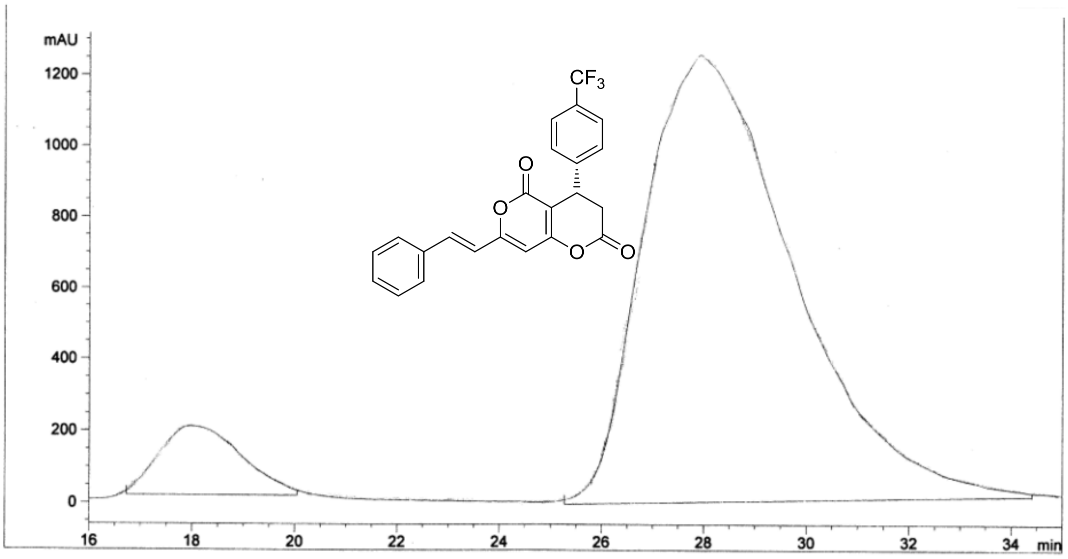

Peak #	RetTime [min]	Type	Width [min]	Area [mAU*s]	Area %	Name
1	17.957	MM	1.8602	2.12705e4	7.7383	?
2	27.923	MM	3.3774	2.53604e5	92.2617	?

HPLC for **9d**

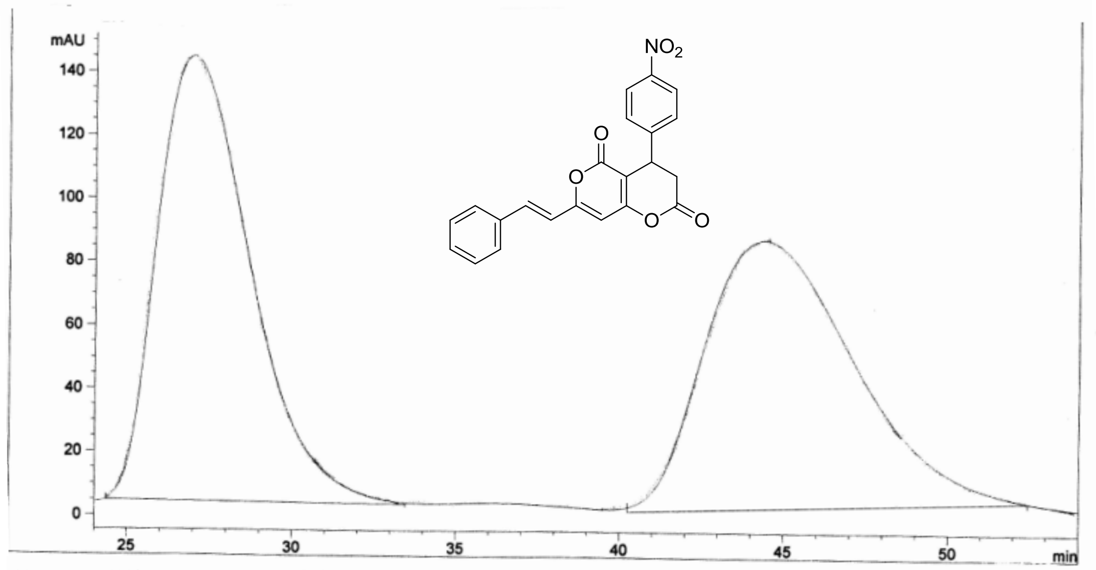

Peak #	RetTime [min]	Type	Width [min]	Area [mAU*s]	Area %	Name
1	27.000	MM	3.2648	2.75340e4	50.7563	?
2	44.413	MM	5.2694	2.67134e4	49.2437	?

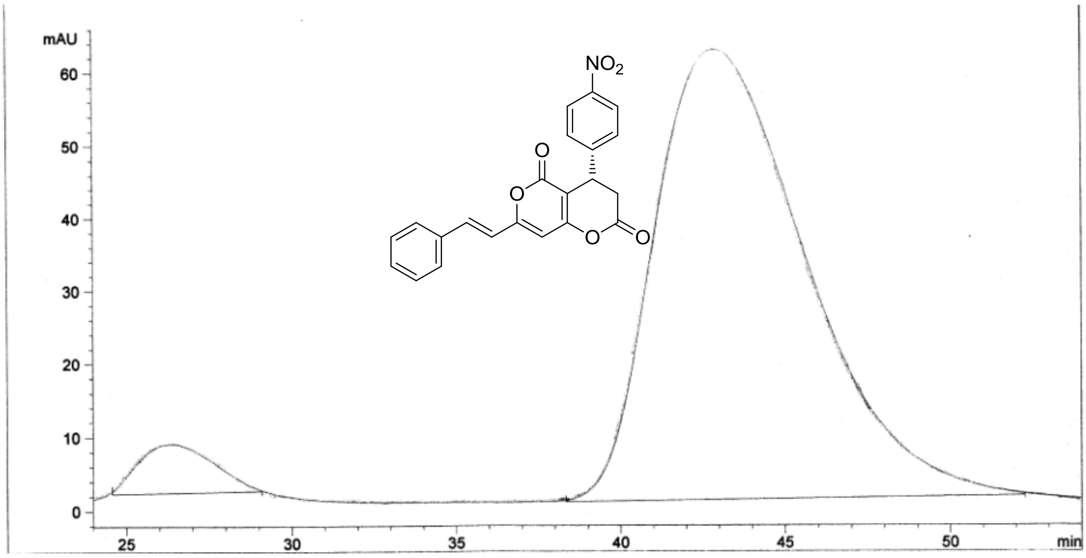

Peak #	RetTime [min]	Type	Width [min]	Area [mAU*s]	Area %	Name
1	26.356	MM	2.6390	1054.45447	5.1151	?
2	42.878	MM	5.2947	1.95602e4	94.8849	?

HPLC for **9e**

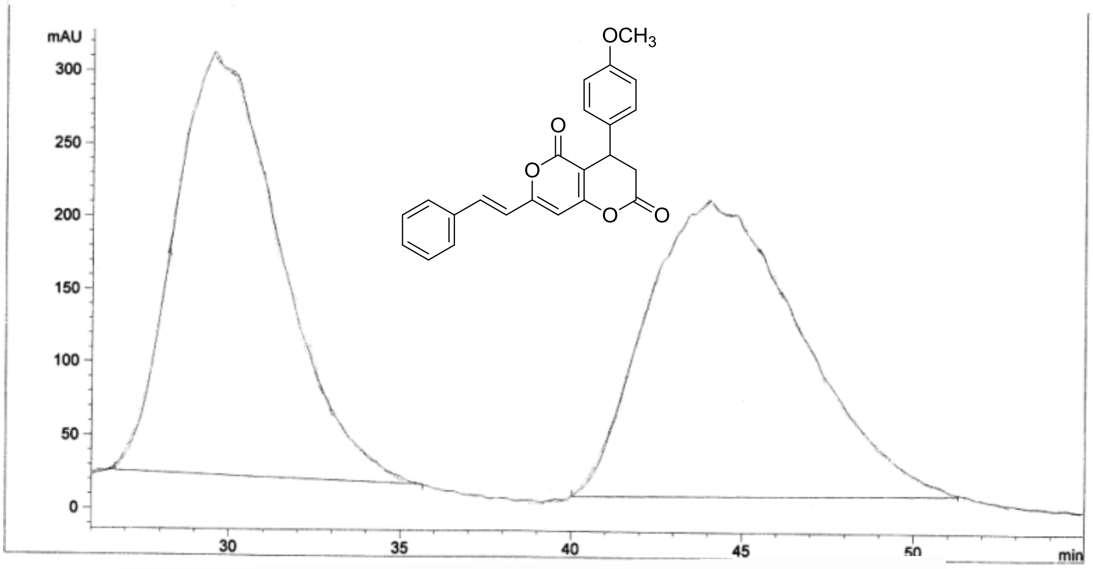

Peak #	RetTime [min]	Type	Width [min]	Area [mAU*s]	Area %	Name
1	29.539	MM	3.5828	6.20875e4	49.3287	?
2	44.027	MM	5.2404	6.37773e4	50.6713	?

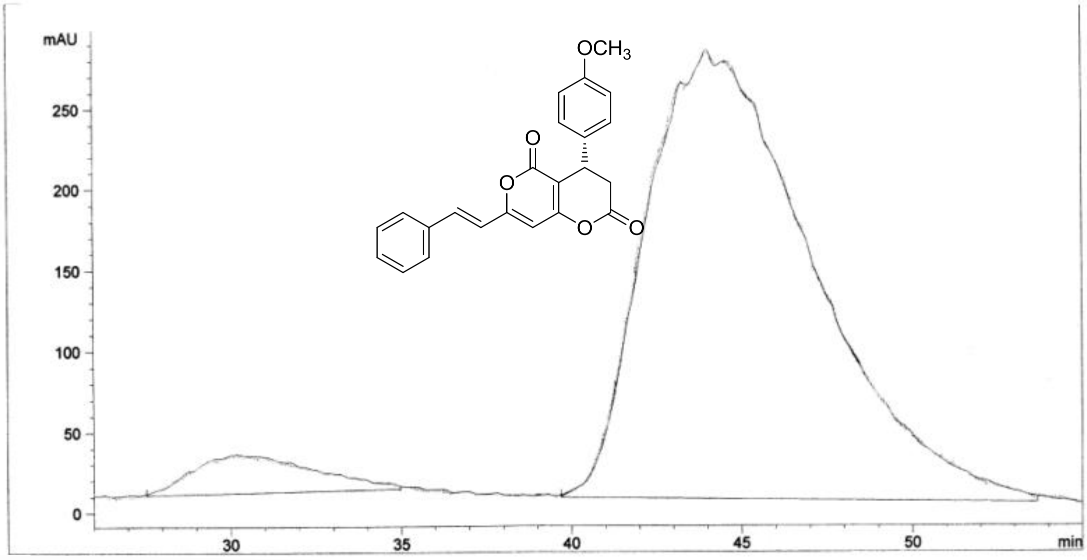

Peak #	RetTime [min]	Type	Width [min]	Area [mAU*s]	Area %	Name
1	30.150	MM	3.1373	3724.26514	3.8082	?
2	44.034	MM	5.6473	9.40709e4	96.1918	?

HPLC for **9f**

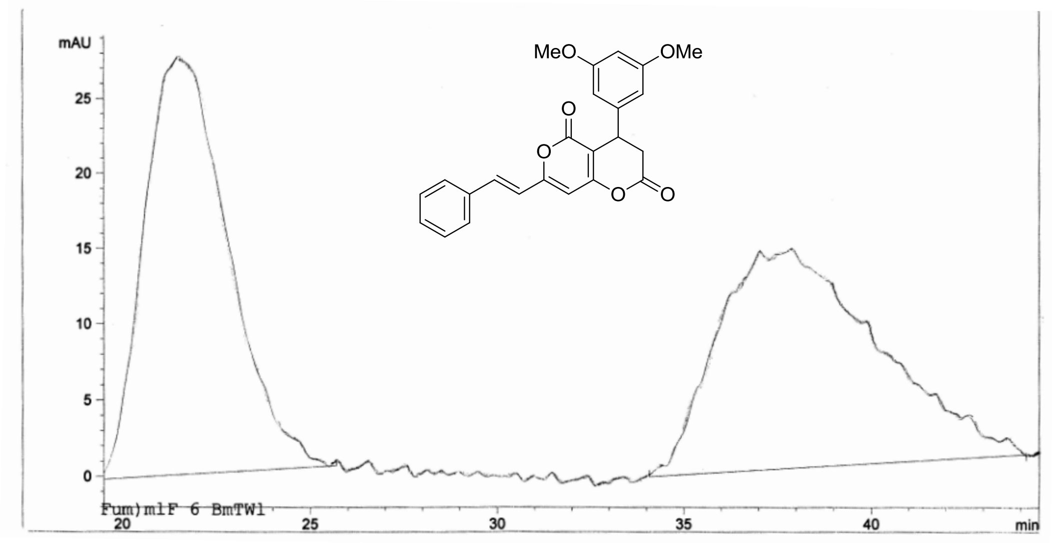

Peak #	RetTime [min]	Type	Width [min]	Area [mAU*s]	Area %	Name
1	21.479	BB	1.8063	4213.09131	49.6571	?
2	37.893	MM	4.9192	4271.28174	50.3429	?

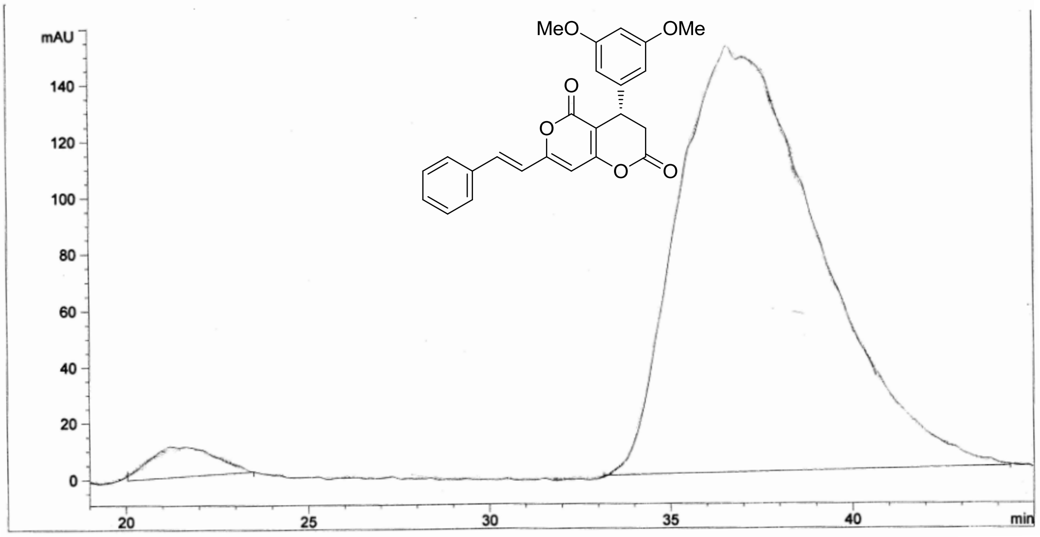

Peak #	RetTime [min]	Type	Width [min]	Area [mAU*s]	Area %	Name
1	21.211	MM	2.0652	1346.85791	3.1833	?
2	36.604	MM	4.5159	4.09639e4	96.8167	?

HPLC for **9g**

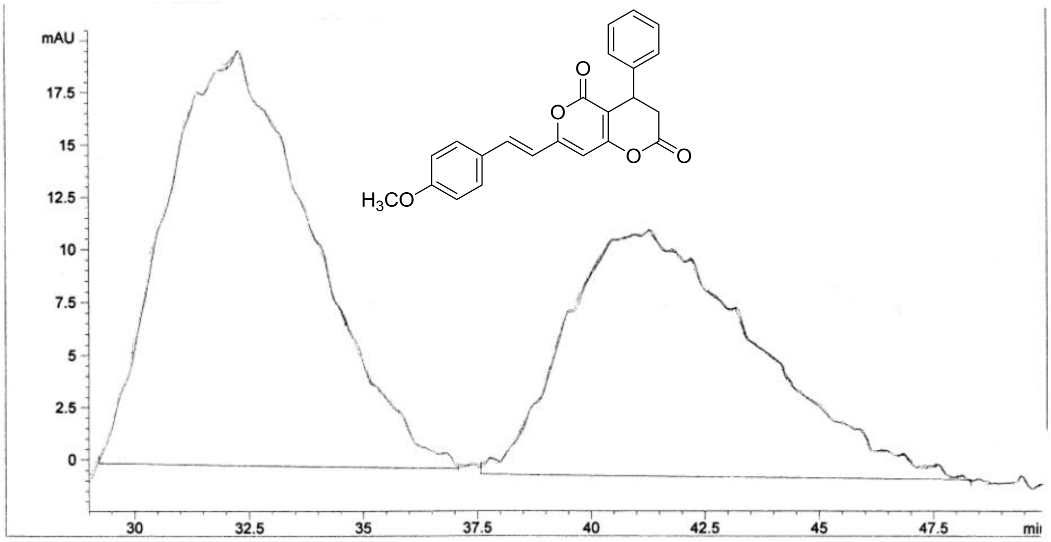

Peak #	RetTime [min]	Type	Width [min]	Area [mAU*s]	Area %	Name
1	32.279	MM	3.3829	3587.62964	51.3378	?
2	41.284	MM	4.8445	3400.64917	48.6622	?

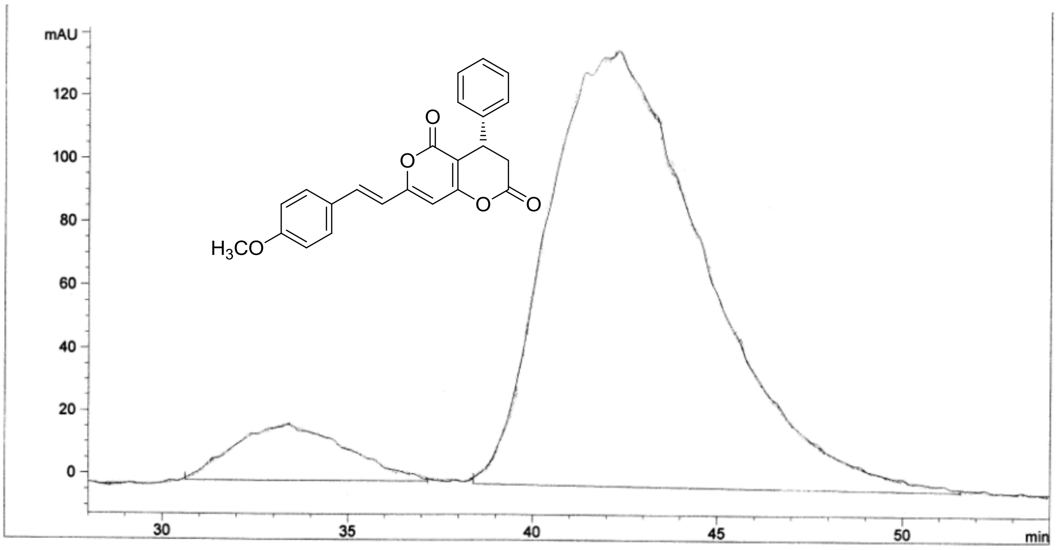

Peak #	RetTime [min]	Type	Width [min]	Area [mAU*s]	Area %	Name
1	33.372	MM	3.5021	3761.86475	8.3755	?
2	42.315	MM	4.9562	4.11532e4	91.6245	?

HPLC for **9h**

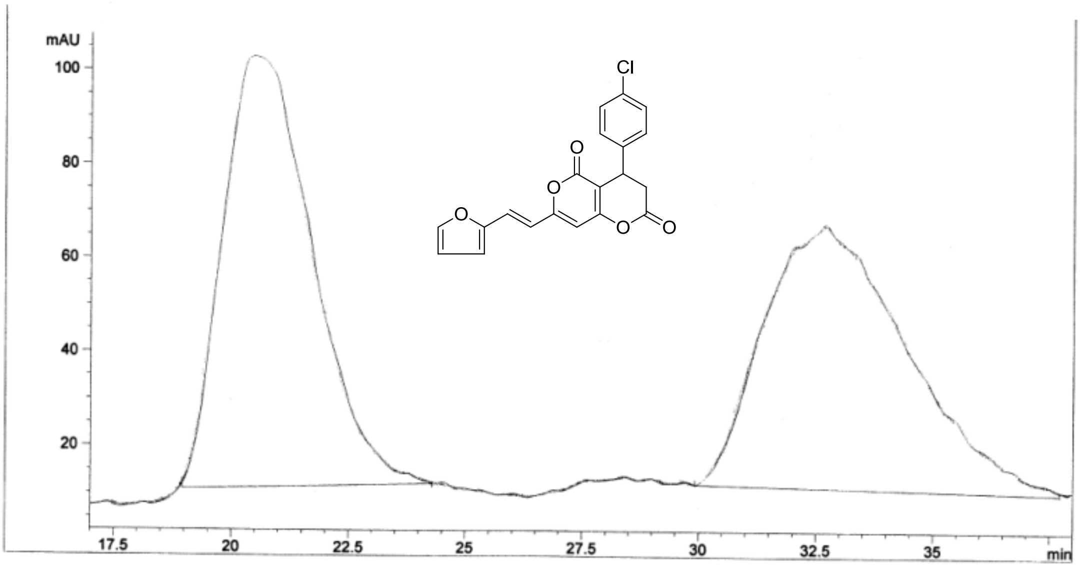

Peak #	RetTime [min]	Type	Width [min]	Area [mAU*s]	Area %	Name
1	20.472	MM	2.2095	1.21485e4	50.6544	?
2	32.694	MM	3.5151	1.18346e4	49.3456	?

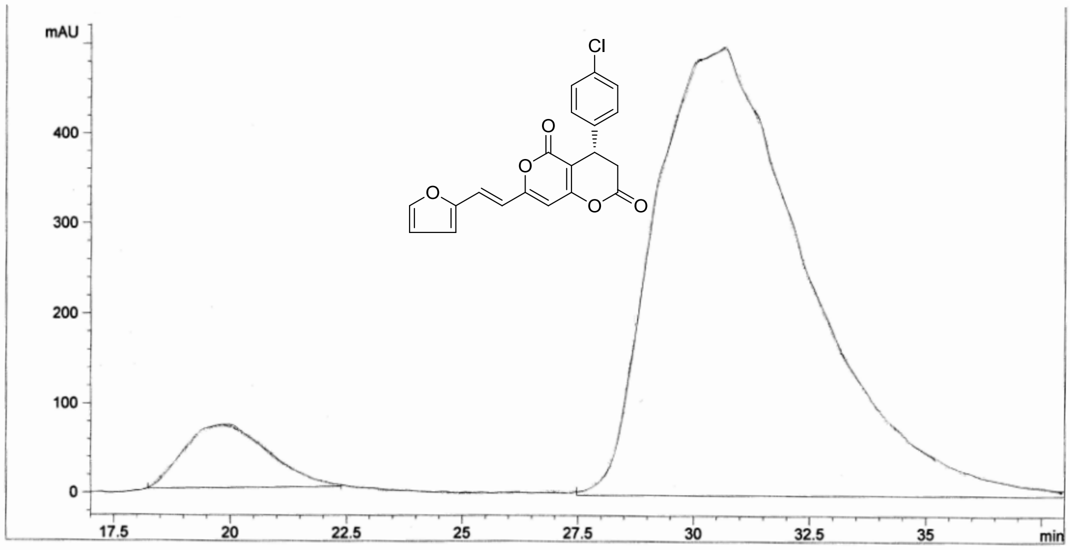

Peak #	RetTime [min]	Type	Width [min]	Area [mAU*s]	Area %	Name
1	19.895	MM	2.1017	8944.27637	7.2491	?
2	30.653	MM	3.8254	1.14441e5	92.7509	?

HPLC for **9i**

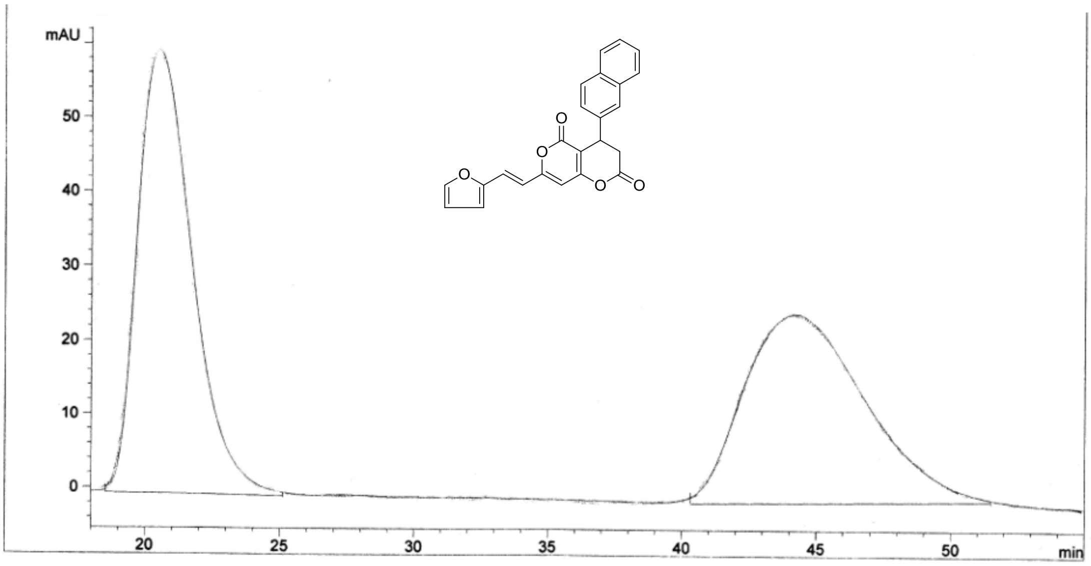

Peak #	RetTime [min]	Type	Width [min]	Area [mAU*s]	Area %	Name
1	20.500	MM	2.3389	8410.24707	51.2692	?
2	44.339	MM	5.2150	7993.85254	48.7308	?

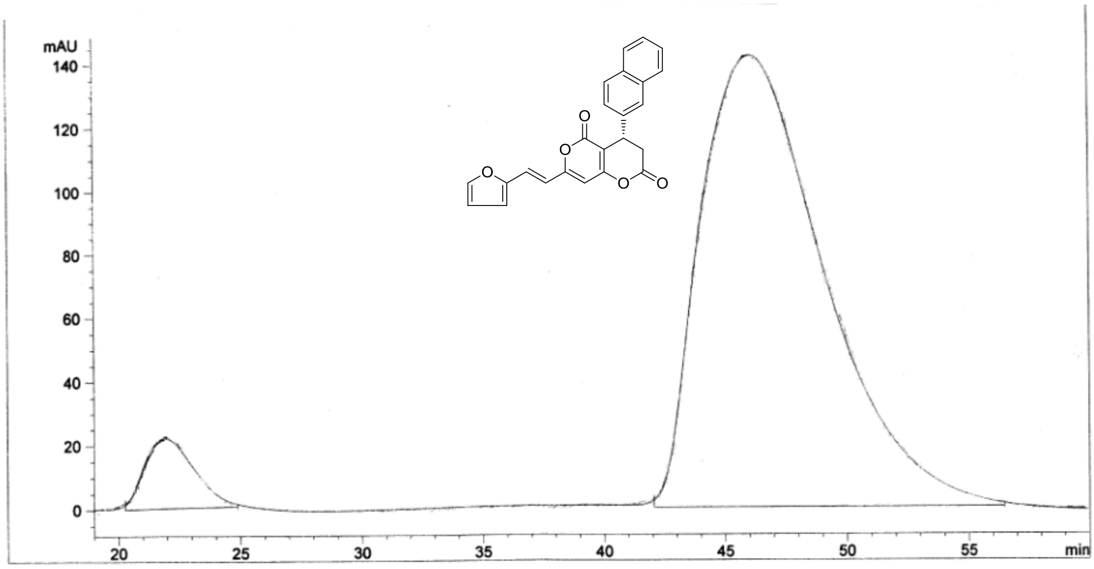

Peak #	RetTime [min]	Type	Width [min]	Area [mAU*s]	Area %	Name
1	21.929	MM	2.3948	3141.45801	5.9236	?
2	46.114	MM	5.8581	4.98910e4	94.0764	?

HPLC for **9j**

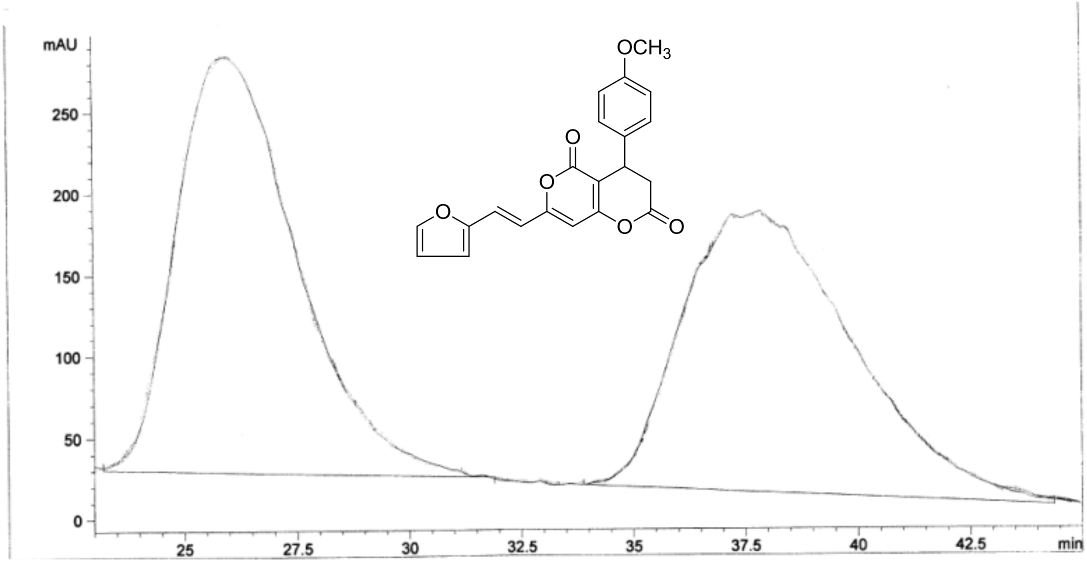

Peak #	RetTime [min]	Type	Width [min]	Area [mAU*s]	Area %	Name
1	25.991	MM	3.0869	4.73929e4	51.1729	?
2	37.865	MM	4.3859	4.52203e4	48.8271	?

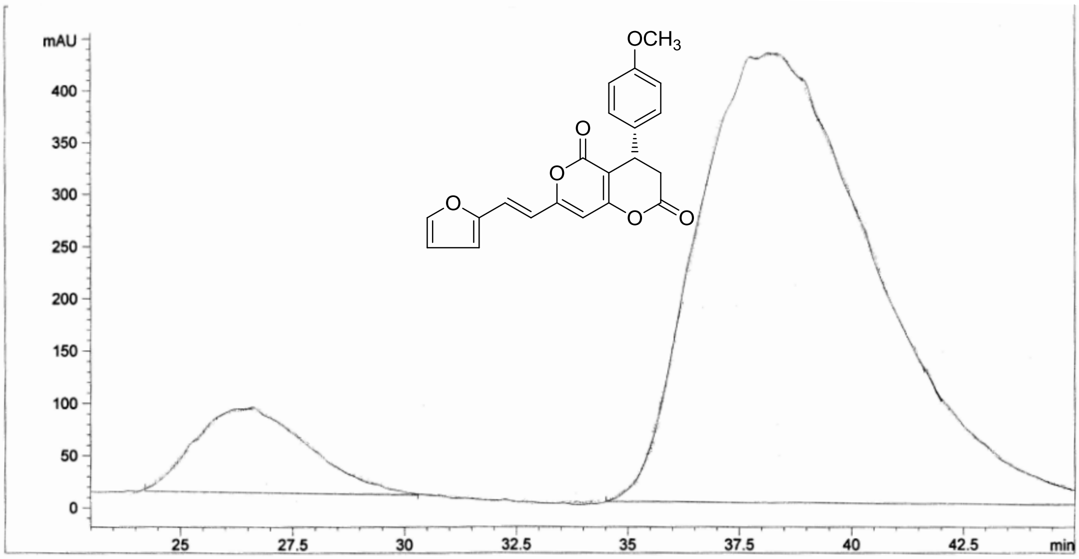

Peak #	RetTime [min]	Type	Width [min]	Area [mAU*s]	Area %	Name
1	26.613	MM	2.9118	1.43113e4	10.6662	?
2	38.151	MM	4.6476	1.19863e5	89.3338	?

HPLC for **9k**

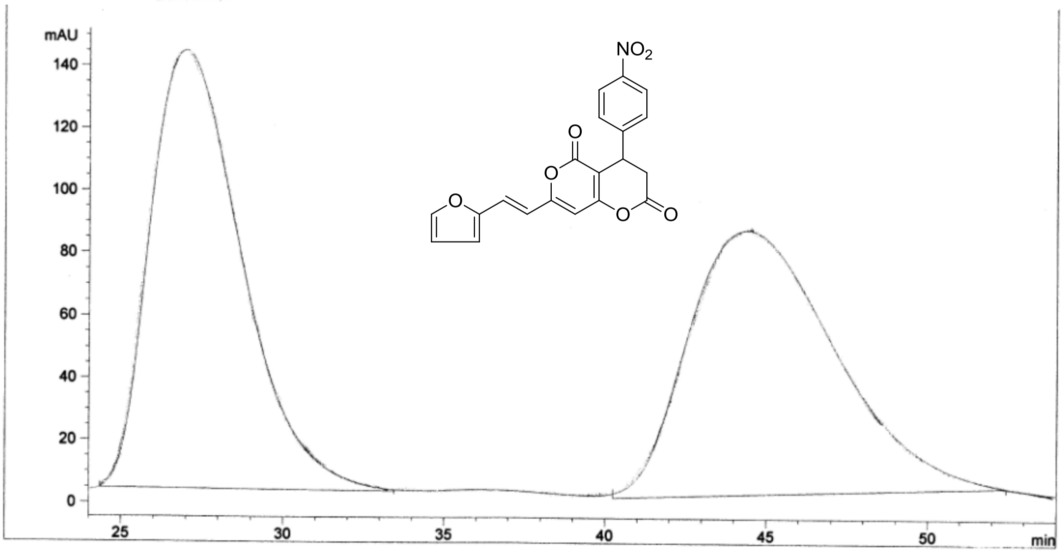

Peak #	RetTime [min]	Type	Width [min]	Area [mAU*s]	Area %	Name
1	22.707	MM	2.9017	1.12243e4	49.5824	?
2	43.397	MM	5.1713	1.14133e4	50.4176	?

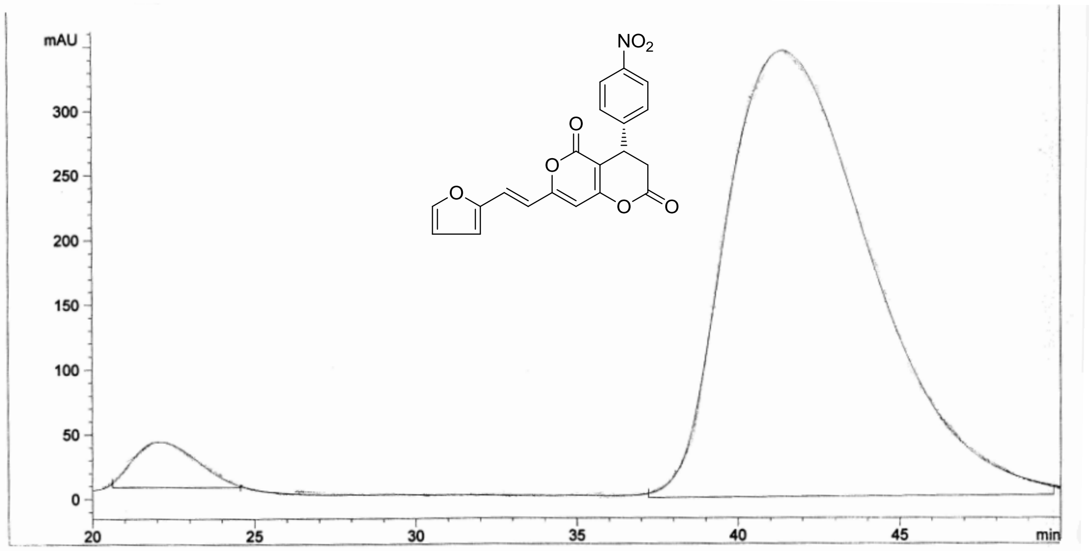

Peak #	RetTime [min]	Type	Width [min]	Area [mAU*s]	Area %	Name
1	22.081	MM	2.2733	4802.72510	4.2792	?
2	41.430	MM	5.1988	1.07430e5	95.7208	?
